# Benefits of cardiac rehabilitation following acute coronary syndrome for patients with and without diabetes: a systematic review and meta-analysis

**DOI:** 10.1186/s12872-022-02723-5

**Published:** 2022-06-27

**Authors:** Birgitte Bitsch Gadager, Lars Hermann Tang, Maiken Bay Ravn, Patrick Doherty, Alexander Harrison, Jan Christensen, Rod S. Taylor, Ann-Dorthe Zwisler, Thomas Maribo

**Affiliations:** 1 Department of Public Health, Centre for Rehabilitation Research, Aarhus University, P.P. Oerumsgade 11, building 1 b, 8000 Aarhus C, Aarhus, Denmark; 2 DEFACTUM, Central Denmark Region, Aarhus, Denmark; 3 The Research Unit PROgrez, Department of Physiotherapy and Occupational Therapy, Naestved-Slagelse-Ringsted Hospitals and The Department of Regional Health Research, University of Southern Denmark, Odense, Denmark; 4 Department of Health Sciences, University of York, York, England; 5 Department of Occupational Therapy and Physiotherapy, Copenhagen University Hospital, Rigshospitalet, Copenhagen, Denmark; 6 MRC/CSO Social and Public Health Sciences Unit and Robertson Centre for Biostatistics, Institute of Health and Well Being, University of Glasgow, Glasgow, Scotland; 7 National Institute of Public Health, University of Suthern Denmark, Odense, Denmark; 8 Department of Cardiology, Odense University Hospital, Odense, Denmark; 9 Department of Clinical Research, University of Southern Denmark, Odense, Denmark; 10 REHPA, The Danish Knowledge Centre for Rehabilitation and Palliative Care, Odense University Hospital, Nyborg, Denmark

**Keywords:** Acute coronary syndrome, Diabetes, Secondary prevention, Cardiac rehabilitation, Multimorbidity

## Abstract

**Aim:**

The benefits of cardiac rehabilitation (CR) after acute coronary syndrome (ACS) are well established. However, the relative benefit of CR in those with comorbidities, including diabetes, is not well understood. This systematic review and meta-analysis examined the benefit of CR on exercise capacity and secondary outcomes in ACS patients with a co-diagnosis of diabetes compared to those without.

**Methods:**

Five databases were searched in May 2021 for randomised controlled trials (RCTs) and observational studies reporting CR outcomes in ACS patients with and without diabetes. The primary outcome of this study was exercise capacity expressed as metabolic equivalents (METs) at the end of CR and ≥ 12-month follow-up. Secondary outcomes included health-related quality of life, cardiovascular- and diabetes-related outcomes, lifestyle-related outcomes, psychological wellbeing, and return to work. If relevant/possible, studies were pooled using random-effects meta-analysis.

**Results:**

A total of 28 studies were included, of which 20 reported exercise capacity and 18 reported secondary outcomes. Overall, the studies were judged to have a high risk of bias. Meta-analysis of exercise capacity was undertaken based on 18 studies (no RCTs) including 15,288 patients, of whom 3369 had diabetes. This analysis showed a statistically significant smaller difference in the change in METs in ACS patients with diabetes (standardised mean difference (SMD) from baseline to end of CR: − 0.15 (95% CI: − 0.24 to − 0.06); SMD at the ≥ 12-month follow-up: − 0.16 (95% CI: − 0.23 to − 0.10, four studies)).

**Conclusion:**

The benefit of CR on exercise capacity in ACS patients was lower in those with diabetes than in those without diabetes. Given the small magnitude of this difference and the substantial heterogeneity in the results of the study caused by diverse study designs and methodologies, further research is needed to confirm our findings. Future work should seek to eliminate bias in observational studies and evaluate CR based on comprehensive outcomes.

**Supplementary Information:**

The online version contains supplementary material available at 10.1186/s12872-022-02723-5.

## Introduction

Cardiac rehabilitation (CR) is highly recommended after acute coronary syndrome (ACS) due to its beneficial effects on cardiac mortality, hospitalisation, and health-related quality of life (HRQoL) [1]. However, ACS patients with multimorbidity are underrepresented in studies evaluating CR [1]. Thus, less is known about the effectiveness of CR and the management of ACS patients living with multiple diseases.

Diabetes is a known risk factor for ACS and more than doubles the risk for cardiovascular disease [2]. The conditions have similar risk factors and are closely related in aetiology [3]. Hence, diabetes is one of the most prevalent comorbidities in CR patients; notably, up to one-third of CR patients have been estimated to have diabetes [4, 5]. Compared to ACS patients without diabetes, those with a combination of ACS and diabetes exhibit a higher mortality, accelerated loss of physical function, and a poorer HRQoL [6–8]. The adverse prognoses for ACS patients with diabetes call for CR interventions adapted to the needs of this high-risk group to ensure effective CR irrespective of having a co-diagnosis [9, 10].

Evidence suggests that intensified, multidisciplinary CR targeting lifestyle and medication is achievable for ACS patients with diabetes and improves their prognosis [11, 12]. Patients with a comorbidity of diabetes should be able to attend CR safely and the fundamental CR recommendations for exercise and healthy lifestyle are considered compatible with diabetic treatment irrespectively of type of diabetes [3, 12, 13]. However, safety precautions as frequent self-monitoring of blood glucose concentration before, during and after exercise are recommended [13]. Despite concordant treatment recommendations, management of patients with diabetes remains suboptimal in CR, and generally, the growing literature on multimorbidity suggests that traditional disease-specific rehabilitation potentially overlooks interactions of multiple diseases and their management [4, 10]. The insufficient management in relation to a co-diagnosis of diabetes could be explained by inherent precautions that might cause differential needs at entry to CR such as diabetes-related comorbidities, glucose-lowering medication use, dietary patterns, self-management and psychosocial wellbeing [12]. These factors might prevent the realisation of the recommended treatment and require a person-centred and multidisciplinary approach [12]. It is therefore important to examine whether these needs are adequately addressed in CR traditionally developed from a disease-specific model and how outcomes are affected [10, 14]. Knowledge in this field may contribute to evolving CR to best address the comprehensive needs of patients with co-diagnoses.

Exercise capacity is a key outcome in CR due to its ability to reduce mortality and morbidity in the general CR population as well as in patients with diabetes [1, 15]. The primary objective of the current review was therefore to examine the benefit of CR on exercise capacity in ACS patients with a co-diagnosis of diabetes compared to those without. Second, the review aimed to examine the benefit of CR on HRQoL, cardiovascular- and diabetes-related outcomes, lifestyle-related outcomes, psychological wellbeing, and return to work in ACS patients with a co-diagnosis of diabetes compared to those without.

## Methods

This systematic review was reported according to the PRISMA statement [16]. The study protocol has been registered in the PROSPERO database (CRD42019151055).

### Study eligibility criteria

Studies published in 2000 or later were included to reflect the current guideline-recommended management of ACS (e.g., up-to-date surgical and medical procedures and secondary prevention) [17]. The study eligibility criteria are presented in Table 1.

**Table 1:** Study selection criteria

Population	Adult patients participating in CR following ACS with and without type 1 or type 2 diabetes ACS includes: Acute myocardial infarction (including ST-elevation myocardial infarction (STEMI)), Non-ST-elevation myocardial infarction (NSTEMI), Stable and unstable angina pectoris. And/or patients who have undergone following revascularisation procedures: Coronary artery bypass grafting (CABG), Percutaneous coronary intervention (PCI)
Intervention	Cardiac rehabilitation interventions *must* include: Supervised or facilitated sessions and structured exercise based training. Sessions can be supervised by a health professional or a structured home programme facilitated in regular follow-up consultations Interventions *can* include: (1) physical activity promotion, (2) patient education, (3) psychological- and psychosocial support, in addition to other related health behaviour change interventions
Comparison	ACS patients undergoing cardiac rehabilitation following acute coronary syndrome *with* a co-diagnosis of diabetes is compared to ACS patients *without* a co-diagnosis of diabetes
Outcomes	Primary: Exercise capacity Secondary: 1) Health-related Quality of Life (HRQoL) 2) Cardiovascular related: Mortality (all-cause or cardiac), Fatal or nonfatal myocardial infarction, Revascularisations (CABG or PCI), Hospital readmission 3) Diabetes related: Blood glucose level, Weight, Body mass index (BMI) 4) Lifestyle related: Smoking status, Physical activity 5) Psychological well-being (patient reported outcomes (PRO) measuring psychological constructs as anxiety, depression, distress) 6) Return to work
Follow-up	1. From start to end of intervention; 2. Long-term: ≥ 12 months post intervention
Study designs	Randomised controlled trials: Randomised controlled crossover trials, Randomised controlled pilot studies. Data reported in RCT studies was allowed for extraction for observational comparison Observational studies: Prospective cohort studies, retrospective cohort studies
Publication year	Studies published in 2000 or later
Language restriction	English, Danish, Swedish, Norwegian

The population comprised two groups: ACS patients with a co-diagnosis of diabetes (exposure) compared to those without (comparison group). Structured exercise training (Table 1) was an inclusion criterion, and other core components for CR could be included in accordance with the British Association for Cardiovascular Prevention and Rehabilitation (BACPR) [18]. Only studies published in 2000 or later were included to reflect the current guideline-recommended management of ACS (e.g., up-to-date surgical and medical procedures and secondary prevention) [17].

### Outcomes

The primary outcome, cardiorespiratory fitness (CRF), referred to as exercise capacity in this paper, was measured directly using a physical test with four possible end points (i.e., VO_2_ max, VO_2_ Peak, sub maximum or symptom-limited). All exercise test results were unified through the use of metabolic equivalents (METs), which were assessed directly by a maximal test (using facial mask monitoring gas exchange) or estimated based on the workload associated with a submaximal test. All MET values were extracted as reported, and VO_2_ reported values were converted into METs assuming 1 MET equals 3.5 ml/kg VO_2_ [19]. Secondary outcomes are outlined in Table 1.

### Search strategy

The search strategy was developed with support from a specialist librarian. Searches in the databases PubMed (U.S. National Library of Medicine, NCBI), EMBASE by Elsevier, Cochrane Central Register of Controlled Trials (CENTRAL), Web of Science (WoS), and CINAHL (via EBSCO-HOST) were conducted on May 24, 2021, using a strategy combining selected MeSH terms or descriptors and free text terms relating to four blocks: (1) ACS, (2) diabetes, (3) CR and (4) study design. Search strategies and search terms are documented in the additional file 1. In addition to the structured search, Cochrane reviews matching the topic "Myocardial ischaemia/coronary disease" in the Cochrane Database of Systematic Reviews were hand searched for eligible studies. The included randomised controlled trials (RCTs) from the most recent Cochrane Review on exercise-based CR were examined, and an updated search was performed in CENTRAL from 2014 to2020 for eligible studies [1]. Furthermore, reference lists of key literature [1, 12, 14, 15] were examined, and ClinicalTrials.gov was searched to identify ongoing studies (see search terms in additional file 1).

### Study selection

The study selection process was conducted using Covidence software (www.covidence.org) [20]. The titles and abstracts were screened independently by at least two of three reviewers (KKWP, MBR, BBG). Next, all full-text articles marked with “yes” or “maybe” were retrieved, and the eligibility of each study was assessed by at least two of three reviewers (BBG, MBR, TM). The primary reason for exclusion of each study was recorded. Any conflicts between the two reviewers were discussed with the third reviewer until consensus was reached.

### Data extraction

A predefined data extraction form was designed and used. Details are outlined in Table 2. Data extraction was performed by the first author consulted by PD, AH or JC. CR interventions in the selected studies were quality checked according to the six core components for cardiovascular disease prevention and rehabilitation outlined by BACPR (see Additional file 2) [18]. For the primary outcome, exercise capacity (METs) at baseline, end of CR and ≥ 12-month follow-up was extracted along with number of patients (n) and standard deviations (SDs) for the two groups, namely, ACS patients with a co-diagnosis of diabetes versus those without.

**Table 2:** Table of characteristics

First author (year) country	Inclusion and description of intervention (a) Enrolled patients in total study population (n) (b) Inclusion period (c) Index event and revascularisation procedure (d) Providing sector (e) Duration and frequency of CR (f) Components of CR (g) Diabetes specific CR components (h)BACPR score		ACS patients with diabetes (a) % of overall enrolled patients (b) Age (years, mean ± SD) (c) sex (% female) Baseline exercise capacity (e) Completion or adherence to CR (f) Proportion of type 1 and type 2 diabetes (g) Duration of diabetes (years)			ACS patients without diabetes (a) % of overall enrolled patients (b) Age (years, mean ± SD) (c) Gender (% female) (d) Baseline exercise capacity (e) Completion or adherence of CR			Results (a) Time point of follow-up (b) Results reported on exercise capacity		Remarks
Banzer 28] (2003) USA	(a) 952 (b) 1993–2001 (c) MI (d) Medical centre, outpatient (e) 10-week program, 30–40 min x three/week. Home-based exercise was recommended (f) Exercise, nutritional counselling, pharmacologic treatment, smoking cessation (g) Not specified (h) 5		(a) 26.2% (b) 62 ± 10 (c) 46% (d) 5.7 ± 2.3 METs (e) 38% attended > 70% of scheduled sessions (f) not reported (g) not reported			(a) 73.7% (b) 61 ± 11 (c) 36% (d) 7.0 ± 2.6 METs (e) 48% attended > 70% of scheduled sessions			(a) Exercise capacity at 10-week follow-up (b) ACS patients with diabetes 7.2 METs (26% change) ACS patients without diabetes 8.9 METs (27% change)		
Vergès [34] (2003) France	(a) 95 (b) not reported (c) MI, unstable angina (d) Outpatient (e) Eight-week program. 70 min, x three /week (f) Exercise, educational sessions (coronary risk factors, smoking, dietary counselling) provided individually and as group discussions (g) not reported (h) 4		(a) 62.1% (b) 57.4 ± 8.8 (c) 13.6% (d) 20.2 ± 5.8 Peak VO2 (ml/kg per min) (e) All patients adhered to at least 92% of all sessions (f) Type 2 DM only (g) 5 years (min–max: 0.2–11.7)			(a) 37.9% (b) 56.7 ± 11.3 (c) 8.3% (d) 22.4 ± 6.3 Peak VO2 (ml/kg per min) (e) All patients adhered to at least 92% of all sessions			(a) Exercise capacity at 8-week follow-up (b) ACS patients with diabetes 22.6 ± 6.7 Peak VO2 (ml/kg per min) (13 ± 24% change) ACS patients without diabetes 28.8 ± 8.6 Peak VO2 (ml/kg per min) (30 ± 25% change)		Peak VO2 converted into METs
Hindman [33](2005) USA	(a) 1505 (b) September 1999 – April 2004 (c) CABG, CAD, MI, and PCI (d) Free-standing community hospital-based (e) 12-week program. 40–50 min x three /week (f) Structured and supervised exercise, individual counselling and group classes on nutrition, heart health, risk factors, stress management, and lifestyle modification (g) Triaging of patients to individual nutrition counselling based e.g. diabetes. Using 24-h food log and guidelines for carbohydrate intake for optimal glucose control (h) 5		(a) 19.4% (b) 63.2 ± 10.7 (c) 27% (e) Overall 5.7 ± 2.3 METs Males: METs 6.2 ± 2.2 Females: METs 4.5 ± 2.0 (f) Patients completing a minimum of 7 weeks of a 12-weeks CR program included (g) Not reported			(a) 80.6% (b) n = 62.1 ± 11.4 (c) 26% (d) METs 7.1 ± 2.6 Men: METs 7.6 ± 2.6 Women: METs 5.6 ± 2.0 (e) Patients completing a minimum of 7 weeks of a 12-weeks CR program included			(a) Exercise capacity at 12-week follow-up (b) ACS patients with diabetes Overall: 7.3 ± 2.4 METs (26.3% change) ACS patients without diabetes Overall: 8.9 ± 2.7 METs (25.5% change)		
Pischke [32] (2006) (USA)	(a) 434 (b) Not reported (c) CAD and CABG/PTCA (d) Hospital based, outpatient (e) 12-h initial seminar + Group sessions × 3/week for. The next 12 weeks: Exercise and lecturers 60 min x two/week Group meeting for the next 40 weeks (f) Aerobic exercise, lectures and demonstrations (e.g., cooking, instructions in stress management) (g) Not reported (h) 5		(a) 21.0% (b) Male: 59 ± 10 Female: 58 ± 11 (c) 40.0% (d) METS (ml O^2^(m/kg) Male: 8.8 ± 2.8 Female: 6.9 ± 2.1 (e) Attended an average of 91% of the group support (first three months) At 1 year, 76% attended group sessions (f) 9.8% with type 1 diabetes (g) Not reported			(a) 79% (b) Male: 58 ± 11, Female: 60 ± 10 (c) 16.6% (d) METS (ml O^2^(m/kg) Male: 10.4 ± 2.9 Female: 8.3 ± 2.8 (e) Attended an average of 92% of the group support meetings (first three months) At 1 year, 78% attended group sessions			(a) Exercise capacity at 12-week; 12-month follow-up (b) 12-week follow-up: Male ACS patients with diabetes 10.8 ± 2.7 METs Male ACS patients without diabetes 11.9 ± 2.6 METs Female ACS patients with diabetes 8.4 ± 2.6 METs Female ACS patients without diabetes 9.0 ± 2.9 METs 12-month follow-up Male ACS patients with diabetes 10.8 2.4 METs Male ACS patients without diabetes (continued) 12.5 ± 2.8 METs Female ACS patients with diabetes 8.5 ± 2.8 METs Female ACS patients without diabetes 10.0 ± 3.0 METs		Results provided stratified by gender and therefore treated separately in meta-analysis
Svacinová [31] (2008) Czech Republic	(a) 77 (b) not reported (c) MI, unstable angina, PCI (d) Outpatient (e) 12-week programme, 50 min × 3/week (f) Aerob training, resistance training (g) Not reported (h) 2		(a) 41.6% (b) 64.3 ± 6.2* (c) 21.9% (d) 17.0 ± 4.6 VO_2peak_kg(ml/kg) (e) All analysed patients completed the program (f) Type 2 only (g) Not reported			(a) 58.4% (b) 60.9 ± 8.2 (c) 33.3% (d) 19.1 ± 4.9: VO_2peak_kg(ml/kg) (e) All analysed patients completed the program			(a) Exercise capacity at 12-week follow-up (b) ACS patients with diabetes 19.3 ± 6.0 VO_2peak_kg(ml/kg) ACS patients without diabetes 21.1 ± 5.3 VO_2peak_kg(ml/kg)		Converted into METs
Mourot [35] (2010) France	(a) 1027 (b) not reported (c) CHD: MI event, PTCA or CABG (d) Rehabilitation centre (e) 6-week program, × 5 times/week (total of 13 h per week) (f) Exercise. Education regarding CHD, risk factors, physical practise (g) DM patients also received education regarding use of devices for self-monitoring glycaemia, injections, and adjusting insulin doses (h) 4		(a) 40.2% (b)56.9 ± 7.9 (c) 18.6% (d) 14 ± 4.3 mLxkg^−1^xmin^−1^ (e) All analysed patients completed CR (f) Type 2 DM only (g) Not reported			(a) 60.0% (b) 56.8 ± 10.3 (c) 15.3% (d) 16.6 ± 5.4 mLxkg^−1^xmin^−1^ (e) All analysed patients completed CR			(a) Exercise capacity at six-week follow-up (b) ACS patients with diabetes *17.7 ± 5.2 VO_2peak_kg(ml/kg) ACS patients without diabetes *22.0 ± 6.4 VO_2peak_kg(ml/kg)		Results on METs were originally provided stratified on interventional procedure (CAGB/PTCA). *Unified data were kindly provided by corresponding author
Karjalainen [36]2012 Finland	(a) 83 (b) not reported (c) CAD (d) Home based, exercise prescription. Daily diary and follow-up at specialists of sports medicine (e) 12-week programme: 60 min × 4/week. Followed by prescription of × 5/week for 12 weeks for an unknown number of weeks (f) Homebased heart rate controlled exercise, daily diary, contacted by specialist of sports medicine at 1 and 3 months (g) Not reported (h) 2		(a) 47% (b) 62 ± 5 (c) 18% (d) 6.5 ± 1.6 METS_MAX_ (e) Training realization did not differ between the patients with DM and No DM group (f) Type 2 DM only (g) Not reported			(a) 53% (b) 62 ± 5 (c) 27% (d) 8.1 ± 2.0 (e) Training realization did not differ between the patients with DM and No DM group			(a) Exercise capacity at six-months follow-up (b) ACS patients with diabetes 6.9 ± 1.7 METs; 23.2 ± 6.6 VO2 peak ACS patients without diabetes 8.4 ± 1.9 METs; 28.1 ± 6.8 VO2 peak		VO2peak converted into METs
Nishitani [37] (2013) Japan	(a) 78 (b) July 2002- February 2005 (c) CABG (d) Hospital based, outpatient (e) 6-months programme, 60 min × 1–2 sessions/week. Patients were encouraged to home-based aerobic exercise (f) Exercise, all participants were instructed to follow diet according to American Heart Association. Educational program regarding CAD and its risk factors was provided by nurses, physicians and dietitians (g) Not described (h) 3		(a) 47% (b) 63.3 ± 10 (d) 22% (e) Peak VO2 (ml kg ^−1^ min^−1^): 13.7 ± 4.0 (f) Mean exercise sessions: 16 ± 14 (g) Type 2 only (h) Not reported			(a) 53% (b) xx (c) 64.1 ± 9 (d) 5% (e) Peak VO2 (ml kg ^−1^ min^−1^): 16.0 ± 4.7 (f) Mean exercise sessions: 18 ± 14			(a) Exercise capacity at six-month follow-up (b) ACS patients with diabetes 19.4 ± 3.8 VO2 peak ACS patients without diabetes 22.9 ± 5.4 VO2 peak		VO2 peak converted into METs
Toste [38] (2014) Portugal	(a) 682 (b) January 2009-June 2013 (c) IHD (d) Hospital based (e) 8–12-week program. 60–90 min × 2 /week (f) Exercise, health education: CAD, nutrition, stress and exercise. Individual counselling (g) Not reported (h) 4		(a) 37.0% (b) 61.6 ± 9.1 (c) 24.5% (d) 7.9 ± 2.1 METs (e) Not reported (f) Type 2 only (g) Not reported			(a) 62.9% (b) 58.6 ± 11.0 (c) 21.2% (d) 9.1 ± 2.4 METs (e) Not reported			(a) Exercise capacity at 8 to 12-week follow-up (b) ACS patients with diabetes Mean change in METs: 1.3 ± 1.2 ACS patients without diabetes Mean change in METs: 1.5 ± 1.2		
Kenttä [39] (2014) Finland	(a) 65 (b) Initiated in 2007 (c) CAD (d) Hospital based (e) First three months: 60 min of homebased training, 4 heart rate-controlled exercise sessions per week Progressively increasing so that the last 6 months = 6 exercise sessions per week (f) Exercise, homebased (g) not reported (h) 2		(a) 46.2% (b) 61.7, standard error of mean (SEM) 1.0 (c) not reported (d) 5.3 (SEM: 0.3) METs (e) not reported (f) not reported (g) not reported			(a) 53.8% (b) 61.3, SEM: 0.9 (c)not reported (d) 6.8 (SEM: 0.3) METs (e) not reported			(a) Exercise capacity at two-year follow-up (b) ACS patients with diabetes 5.7 (SEM 0.3) ACS patients without diabetes 7.3 (SEM 0.3) METs		
Armstrong [40] (2014) Canada	(a) 8582 (b) 1996–2010 (c) CAD, PCI, CABG (d) A centralised CR centre, outpatient (e) 12-week program, 60 min × 2/week. Home-based exercise was recommended (f) Exercise: aerobic training, stretching and/or resistance training. Offered sessions of nutrition and stress management, referral to dietician or social worker if needed (g) Not reported (h) 5				(a) 22% (b) 60.1 (no SD)* (c) 28.3% (d) Men: 7.4 METs Women:6.6 METs (e) Completion of CR (completers of baseline test and 12- week test): 1230 (79.6%) (f) Not reported (g) Not reported		(a) 78% (b) 58.9 (no SD) (c) 26.5% (d) Men: 8.4 METs Women: 7.1 METs (e) Completion of CR (completers of baseline test and 12- week test) 5973 (84.9%)	(a) Exercise capacity at 12 weeks; 12-month (b) 12-week follow-up Male ACS patients with diabetes 8.3 METs Male ACS patients without diabetes 9.4 METs Female ACS patients with diabetes 7.3 METs Female cardiac patients without diabetes 8.0 METs 12-month follow-up Male ACS patients with diabetes 8.0 METs Male ACS patients without diabetes 9.3 METs Female ACS patients with diabetes 7.1 METs Female ACS patients without diabetes 8.0 METs		Results provided stratified by gender and therefore treated separately in meta-analysis Missing SD imputed from median observed SD	
Boukhris [41] (2015) Italy	(a) 122 (b) January 2012- August 2013 (c) CAD, PCI and CABG (d) Out-patient (e) 5-week program, 70 min × 4/ week (f) Exercise, psychological and dietary counseling. Patients were encouraged for 1–3 homebased exercise/week (g) Not reported (h) 3				(a) 48% (b) 59.4 ± 8.7 (c) 11.9% (d) 7.3 ± 2.8 METs (e) Not reported (f) Type 2 diabetes only (g) 4.3 ± 2.6 years		(a) 52% (b) 61.6 ± 10.1 (c) 11.1% (d) 7.3 ± 3.3 METs (e) Not reported	(a) Mean change in exercise capacity ± SD at five-week follow-up (b) ACS patients with diabetes + 2.9 ± 2.1* (39.7% improvement) ACS patients without diabetes + 3.3 ± 2.4* (45.2% improvement)			
Kim [42] (2015) Korea	(a) 37 (b) February 2012-January 2014 (c) PCI following MI (d) Hospital-based, outpatient. Continued follow-up at an outpatient clinic every three month (e) 8-week programme, 60 min at least 4–8 sessions (f) Exercise training, information concerning MI, pharmacology, risk factors, nutritional counselling, anti-smoking education (g) Specific recommendations were provided to patients with diabetes (h) 5				(a) 32% (b) 57.0 ± 9.0 (c) 17% (d) 6.5 ± 0.9 METs 22.7 ± 3.0 VO_2peak_ (e) Not reported (f) Type 2 only (g) 50% had newly diagnosed diabetes at the time of MI Average morbidity period was 5.33 ± 3.64 years among those with known diabetes		(a) 68% (b) 55.7 ± 8.4 (c) 4% (d) 7.2 ± 1.1 METs 25.2 ± 3.7 VO_2peak_ (e) Not reported	(a) Exercise capacity at 8-week; 12-month follow-up (b) 8-week follow-up ACS patients with diabetes 7.2 ± 0.8 METs; 25.3 ± 2.7 VO2peak ACS patients without diabetes 8.2 ± 1.5 METs; 28.6 ± 5.1 VO2peak 12-month follow-up ACS patients with diabetes 7.2 ± 1.2 METs; 25.2 ± 4.1 VO2peak ACS patients without diabetes 8.1 ± 1.7 METs; 28.7 ± 5.3 VO2peak		Provided METs used for meta-analysis	
Szalewska [43] (2015) Poland	(a) 125 (b) January 2010-December 2013 (c) CAD (d) Outpatient rehabilitation centre and homebased tele rehabilitation (e) Outpatient phase: 8–10 days Homebased phase: 11–12 days, 30 min × 5/week (f) Outpatient phase: exercise, education, relaxation, secondary prevention strategies. Home-based phase: Endurance training, supervised exercise training, daily mobile phone communication (g) In patients with DM blood glucose levels were initially obtained before and (continued) after exercise to provide an assessment of the individual’s response to exercise (h) 3				(a) 29.6% (b) 59.1 ± 3.91 (c) 8.1% (d) 6.81 ± 1.91 METs (e) Mean number of days of absence in CR 1.22 ± 2.76 (f) Type 2 only (g) Not reported		(a) n = 88 (70.4%) (b) 57.86 ± 4.66 (c) 11.4% (d) 8.31 ± 2.71 METs (e) Mean number of days of absence in CR 1.61 ± 4.51	(a) Exercise capacity ± SD; mean change ± SD at mean follow-up 22 days (b) ACS patients with diabetes 8.30 ± 2.04 METs; + 1.49 ± 2.08 ACS patients without diabetes 9.13 ± 2.87 METs; + 0.81 ± 1.91			
Khadanga [44] (2016) USA	(a) 898 (b) Not reported (c) CAD: MI, CABG, PCI, chronic stable angina, systolic congestive heart failure (d) Medical center, outpatient (e) 3–4 months-programme. 45–60 min × 3/week. Encouraged to exercise on non CR days (f) Exercise, two class room teachings on heart healthy diet. Behavioral weight loss sessions advised for patients being overweight (g) Not reported (h) 5				(a) 22.6% (b) 64.1 ± 10.9* (c) 32.6%* (d) METs: 6.6 ± 2.4 Peak VO_2_ mLO/kg/min: 17.3 ± 5.8* (e) 67.0% completed the program (f) Type 2 only (g) Not reported		(a) 33.7%, (no insulin resistance group formed the comparison group) (b) 62.5 ± 10.8 (c) 21.5% (d) METs: 7.9 ± 2.9 Peak VO_2_ mLO/kg/min: 21.8 ± 6.8 (e) 60.8% completed the program	(a) Exercise capacity ± SD; mean change ± SD at three to four-month follow-up (b) ACS patients with diabetes 7.5 ± 2.7 METs; 20.2 ± 5.5 Peak Vo2; + 1.3 ± 2.3 ACS patients without diabetes 10.2 ± 3.4 METs; 25.5 ± 7.8 Peak Vo2; + 2.2 ± 2.5		*No insulin resistance group formed the comparison group (without diabetes) VO2 peak converted into METs	
Kasperowicz [45] (2019) Poland	(a) 100 (b) 2005–2015 (c) MI treated with invasive procedures (d) Hospital based (e) 12 week programme, dose not reported (f) Exercise (g) Not reported (h) 2				(a) 40% (b) 59.3 ± 7.7 (c) 35% (d) 7.2 ± 2.0 (e) not reported (f) not reported (g) not reported		(a) 60% (b) 57.6 ± 7.8 (c) 40% (d) 7.2 ± 2.0 (e) not reported	(a) Exercise capacity ± SD; mean change at three-week follow-up (b) ACS patients with diabetes 7.7 ± 2.2; + 0.5 ACS patients without diabetes 8.4 ± 1.7; + 1.2			
Laddu (2020) [46] Canada	(a) n = 3953 (analysed patients were propensity matched from entire population) (b) January 1996- March 2016 (c) Cardiac catheterization and/or revascularization (d) Hospital based (e) 12-week programme, 60 min × 2 /week (f) Exercise and individualized education. Support with risk factor management, and access to a multidisciplinary team of healthcare providers (g) Measurement of blood glucose at exercise sessions for DM patients (h) 4				(a) 18.7% (b) 62.6 ± 9.4 (c) 19% (d) 6.7 ± 1.9 METs (e) not reported (f) Type 2 only (g) not reported		(a) 81.3% (b) 62.7 ± 10.7 (c) 20% (d) 7.2 ± 2.1 METs (e) not reported	(a) Exercise capacity mean change ± SD at 12-week follow-up (b) ACS patients with diabetes 0.9 ± 0.9 (13.0%) ACS patients without diabetes 1.0 ± 1.0 (13.2%)			
Eser (2020) [47] Eight European countries	(a) n = 1633 (b) September 2015 to January 2018 (c) Acute and chronic coronary artery disease (CAD) patients and patients after valve intervention (VHD) with an age 65 or above (d) Rehabilitation centres in eight european centres: Bern, Copenhagen, Ludwigshafen, Paris, Parma, Nijmegen, Santiago de Compostela and Zwolle (e) 3-weeks to 3-months programme, 10–36 sessions depending on centre (f) Endurance training, Four of the eight centres also added between 15 and 24 sessions of resistance training, dietary counselling in all centres (continued) (g) not reported (h) 3				(a) n, end of CR = 354, n 1 year = 311* (b) 72.6 ± 5.5 (c) 19.1% (c) 14.51 (4.01) VO2 peak VO2 peak was significantly reduced by 1.46 ml/kg/min at baseline (adjusted for index intervention, sex, age, BMI, comorbidity and cardiovascular risk factors) (d) 94%, (interquartil range 83–100%) (e) Type 1 and type 2 (f) previous diagnosis with DM, intake of insulin or oral antidiabetics at start of CR, HbA1c at baseline of ≥ 48 mmol/mol (g) not reported		(a) n, end of CR = 976, n, 1 year = 891* (b) 73.0 ± 5.4 (c) 24.3% (d) 16.86 (4.89) VO2 peak (e) 100%, (interquartile range 87–100%)	(a) Exercise capacity ± SD at end of CR (T0-T1), and 1-year follow-up (T0-T2) (b) *By end of CR: ACS patients with diabetes (n = 354): 16.47 (4.41), ACS patients without diabetes (n = 976): 18.87 (5.23) *12-months follow-up ACS patients with diabetes (n = 311): 16.79 (4.47) VO2 peak ACS patients without diabetes (n = 891): 19.68 (5.45) VO2 peak		* Upon request: VHD group has been excluded in data for the meta-analysis kindly provided by first author From mixed model adjusted VO2 peak improved in both groups, but with a significantly smaller change in patients with DM (from T0-T2) (-0.6 ml/kg/min) (from additional file 1: S1)	
Studies excluded for meta-analysis											
First author (year) country		Inclusion and description of intervention (a) Enrolled patients in total study population (n) (b) Inclusion period Index event and revascularisation procedure (d) Providing sector (e) Duration and frequency of CR (f) Components of CR (g) Diabetes specific CR components		ACS patients with diabetes (a) % of overall enrolled patients (b) Age (years, mean ± SD) (c) sex (% female) (d) Baseline exercise capacity (e) Completion or adherence to CR (f) Proportion of type 1 and type 2 diabetes (g) Duration of diabetes (years)		ACS patients without diabetes (a) % of overall enrolled patients (b) Age (years, mean ± SD) (c) Gender (% female) (d) Baseline exercise capacity (e) Completion or adherence of CR			Results Time point of follow-up Results reported on exercise capacity		Remarks
Wu [] (2012) Taiwan		(a) 61 (b) not reported (c) CABG (d) Outpatient facility (e) 12 weeks. Three sessions/week each lasting 30 min (f) Exercise only (g) Not reported		(a) 36.0% (b) not reported (c) not reported (d) not reported (e) not reported (f) not reported (g) not reported (h) not reported		(a) 63.9% (b) not reported (c) not reported (d) not reported (e) not reported			(a) Exercise capacity at 12-week follow-up (b) Results only provided in graph		Results only provided in graph
St. Clair [] (2013) USA		(a) 1312 (b) 2004–2012 (c) CAD, CABG and or valvular disease (d) Medical centre, outpatient (e) 12-week programme, 3 sessions/week (f) Exercise. Health and nutrition education sessions (g) Not reported		(a) 28% (b) xx (c) 62 ± 10 (d) 32% (e) 2.4 ± 0.6 METs (f) Not reported (g) Not reported (h) Not reported		(a) 72% (b) xx (c) 63 ± 12 (d) 28% (e) 2.7 ± 0.9 METs (f) Not reported			(a) Exercise capacity mean change (95% CI) at 12-weeks (b) ACS patients with diabetes + 1.7 (1.5–1.9) METs ACS patients without diabetes + 2.5 (2.4–2.7) METs		Low baseline METs

*ACS* Acute coronary syndrome, *AMI* acute myocardial infarction, *MI* Myocardial infarction, *CAD* coronary artery disease, *CHD* Coronary heart disease, *PCI* Percutaneous coronary intervention, *CABG* coronary artery bypass grafting, *PTCA* coronary angioplasty, *DM* diabetes mellitus, *BACPR* British Association for Cardiovascular Prevention and Rehabilitation

### Risk of bias assessment

The risk of bias judgements were assessed independently by two authors (BBG and MBR). Individual assessments were compared, and consensus was reached in discussion with a third author (TM). The Cochrane risk-of-bias tool for randomised trials, version 2 (RoB 2.0), was used to assess the risk of bias in the RCTs [21]. A modified version of the Risk Of Bias In Non-randomized Studies of Exposures (The ROBINS-E) was used to assess the risk of bias in the observational studies [22]. The modification of the ROBINS-E included leaving out domain 2 (selection of participants into the study) and domain 4 (departures from intended exposures) from the assessment. Domain 2 seemed irrelevant, as the exposure (diabetes) is a chronic condition. Instead, the definition of diabetes was extracted for all studies (Additional file 4). Signalling questions for domain 4 were found to be non-applicable for the aim of this study, e.g., "Was selection of participants into the study (or into the analysis) based on variables measured after the start of the exposure?". Instead, loss to follow-up from the study populations was noted. The studies were assessed individually in the remaining domains. Each domain was judged as *low, moderate, serious, or critical*. Finally, an overall risk of bias judgement was made for each study. The ROBINS-E assessment was visualised by a traffic light plot adapted from the visualisation tool robvis provided in the web app [23].

### Statistical analysis

For the primary outcome, the MET change scores for each group were extracted or generated by subtracting the end of CR and 12-month METs from the baseline METs. The baseline and 12-month MET SDs were obtained from the standard error of the mean (SEM) when missing [25]. Regarding the change score SDs, imputation of these SDs was calculated in case of incomplete statistical information using a correlation coefficient or by using summary statistic level imputation [24, 25]. To evaluate the impact of the imputation strategy, a sensitivity analysis was applied based on the median observed SD from studies using an estimated cardiopulmonary exercise test (serving as the worst-case scenario) and studies using a direct cardiopulmonary exercise test (serving as the best-case scenario). The difference in change scores between the groups was calculated by a random-effects model adjusting to Hedges’ g, using change scores and change score SDs, and reported as the standardised mean difference (SMD) with 95% confidence intervals (CI) [25]. The SMD was interpreted according to the Cochrane Handbook guiding rules for interpreting SMDs [26]. Statistical heterogeneity was examined using the Cochrane Q test, quantified with the I^2^ statistic and interpreted according to the thresholds for the interpretation of the I^2^ statistic in the Cochrane Handbook [27]. Publication bias was assessed by Egger’s test and visually by a funnel plot [25]. A number of subgroup analyses were planned, and a detailed description can be found in the PROSPERO protocol (CRD42019151055). Subgroup analyses were performed by random-effects models as described above using meta-regression analyses. If planned subgroup analyses were not possible, reasons for this were addressed.

## Results

**Fig. 1:**
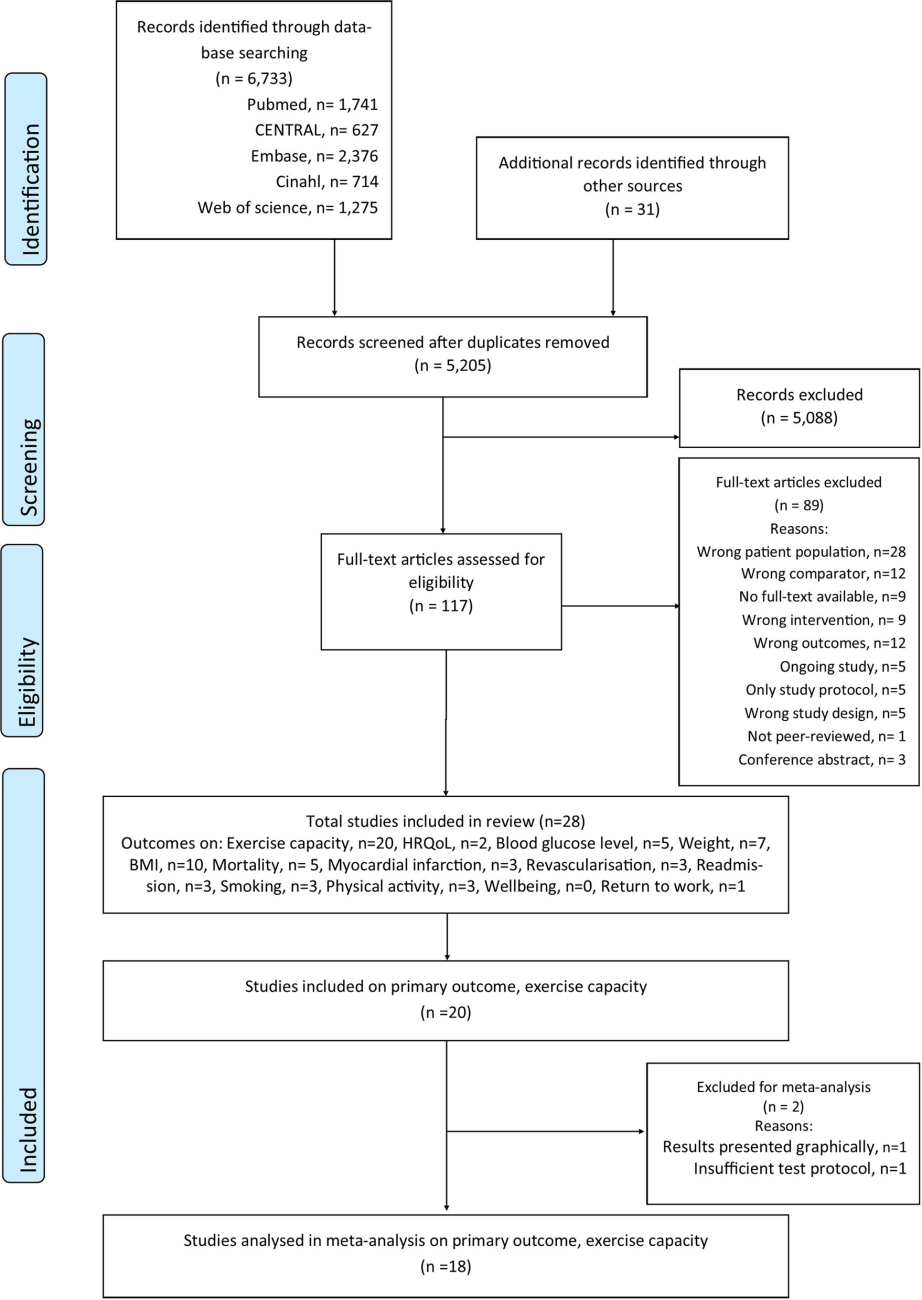
PRISMA flow diagram

The search yielded a total of 5,205 unique studies. The full text of 117 of these studies was assessed for eligibility, with 28 studies eligible for inclusion (Fig. 1). In total, 20 studies reported on the primary outcome, exercise capacity [28–47]. Of these, one RCT was eligible for inclusion [29]; however, only observational data were extracted for the purpose of this review. Ten of the studies reporting on exercise capacity also included reporting on one or more of the secondary outcomes used in this systematic review, and an additional eight studies from the literature search were identified reporting on secondary outcomes; thus, in total, 18 studies were used to assessed secondary outcomes.Additional file 3 contains references and results on secondary outcomes. Hence, in total, 28 studies were included in the current review.

### Study characteristics

Additional file 3 presents the study characteristics and reporting on secondary outcomes. A total of 16,661 ACS patients were included from the 20 studies reporting on exercise capacity. For the meta-analysis, two studies were subsequently excluded due to insufficient reporting of the test protocol [30] and results only being presented graphically [29]. Thus, n = 15,288 patients were analysed at the end of CR in the 18 studies included in the meta-analysis evaluating exercise capacity [28, 31–47]. Table 2 presents detailed information on the included studies.

Across the studies reporting on exercise capacity, 19–48% of the patients were diagnosed with diabetes. The total number of ACS patients with a co-diagnosis of diabetes was 3,369 (22.0%]. ACS patients with type 2 diabetes were exclusively included in 11 studies [31, 34–36, 38, 39, 41–44, 46]. Four studies included ACS patients with type 1 or type 2 diabetes [29, 32, 40, 47], and five studies did not account for the type of diabetes [28, 30, 33, 37, 45]. A diagnosis of diabetes was classified from a fasting blood glucose test or from hospital records in 11 of the studies [34–38, 40–42, 44, 46, 47]. In seven studies, diabetes was classified from a self-reported history, taking diabetes medication, or a lack of information on classification [28, 31–33, 39, 43, 45]. Additional file 4 presents specific classification procedures.

The CR programmes described in the studies reporting the primary outcome were provided as outpatient services lasting from 22 days to two years and were provided in a hospital, medical centre or community-based centre. Home-based interventions with outpatient consultations were reported in three studies [36, 39, 43]. The number of weekly sessions was 1–5, and each session lasted from 30–90 min. In addition to exercise sessions, CR components compromised educational sessions (risk factor management, psychological management and nutritional counselling). In four studies, the intervention was only reported as exercise [29, 31, 36, 39]. However, when providing a quality check of all the interventions according to the BACPR core components (Additional file 2), all of the studies were assessed as comprising elements of "lifestyle risk factor" and "audit and evaluation". Thirteen studies reported elements related to "health behaviour change and education" [28, 32–35, 37, 38, 40, 42–44, 46, 47]. However, less reported were the elements of "psychosocial health" (seven studies) [28, 32, 33, 38, 40, 41, 44], "medical risk management" (seven studies) [28, 33–35, 38, 42, 46], and "long-term strategies" (three studies) [32, 42, 44].

Adherence or compliance to the CR intervention was missing or inconsistently addressed in the majority of the studies. Four studies [28, 32, 40, 47] reported lower measures of adherence or compliance among ACS patients with a co-diagnosis of diabetes, whereas one study oppositely reported higher adherence [44].

### Risk of bias

**Fig. 2:**
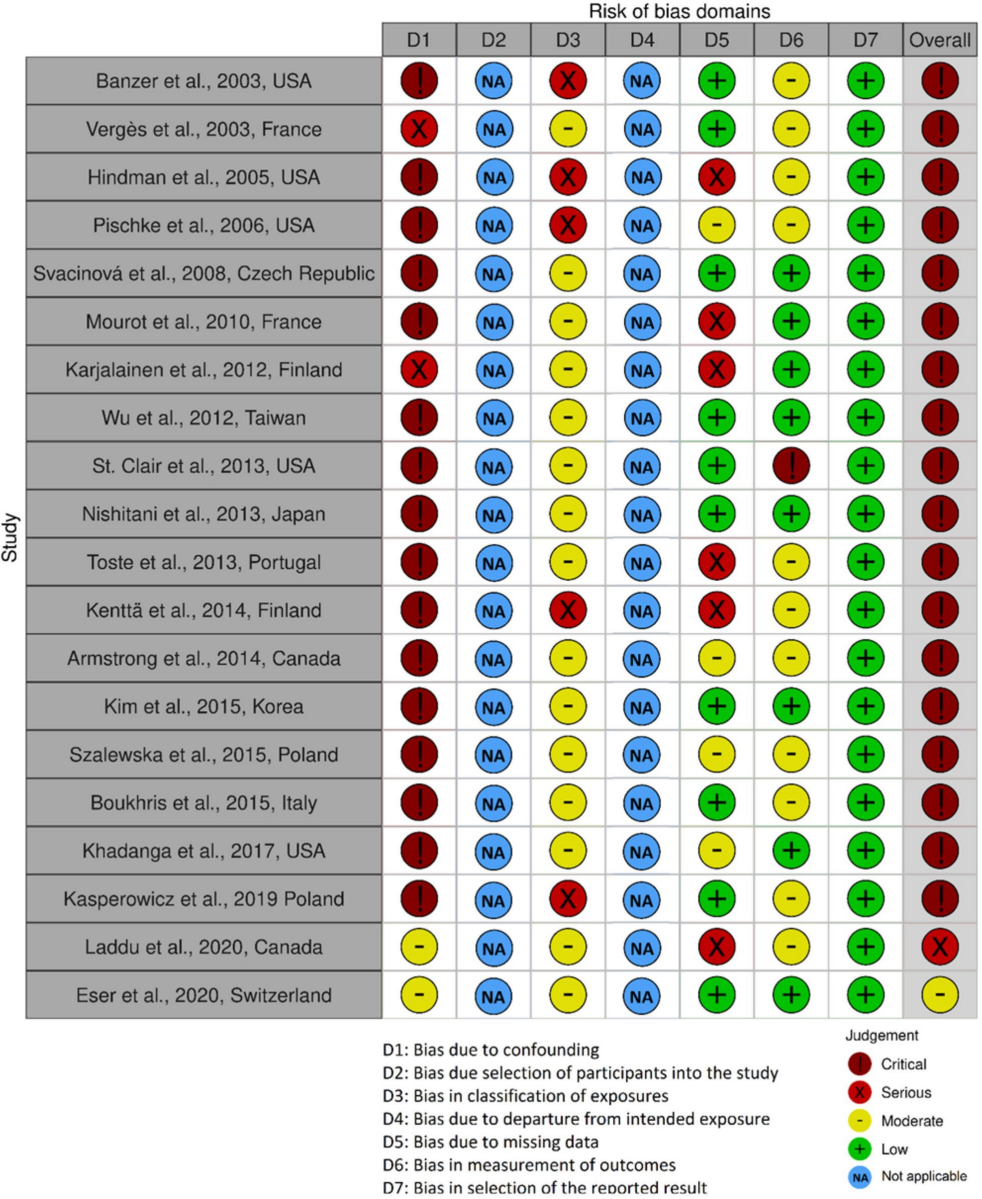
Risk of bias judgement of the included studies

Risk of bias assessments were performed on all 20 studies reporting on exercise capacity, and the assessments are summarised in Fig. 2. For the studies reporting on exercise capacity, two were assessed as having a serious or moderate bias [46, 47], and the rest were assessed as having a critical risk of bias. Limitations were mainly related to bias due to confounding, classification of exposure and outcome as well as risk of bias due to missing data.

### Test procedures for measuring exercise capacity

All 20 studies measuring exercise capacity applied the same cardiopulmonary exercise test procedure for the baseline test as for the follow-up test. Exercise capacity estimated from the maximal work rate achieved was performed in eleven of the studies [28, 30, 32, 33, 38, 40, 41, 43–46], while direct measurement of V̇O_2_ was performed in nine studies [29, 31, 34–37, 39, 42, 47]. A ramp loading of gradual resistance was applied in six studies [28, 29, 35, 37, 41, 47], whereas two studies [34, 36] reported incremental loading. In 12 studies [30–33, 38–40, 42–46], the loading procedure was not specified. A treadmill was used in 12 studies [28, 32, 33, 35, 38, 40–46], and seven studies used a bicycle ergometer [29, 31, 34, 36, 37, 39, 47]. In one study, the test device was not clear [30]. Exercise capacity was reported as metabolic equivalents (METs), VO_2_peak (ml O_2_/kg per minute) or both. Follow-up was performed after the final CR session in all 20 studies. In four studies [32, 40, 42, 47], follow-up was also performed at 12 months from baseline. Additional file 5 presents the specific test methods. Two studies were excluded from the meta-analysis due to results only being presented graphically [29] and insufficient reporting of the test protocol [30].

### Comparison of changes in exercise capacity from the start to the end of the intervention

**Fig. 3:**
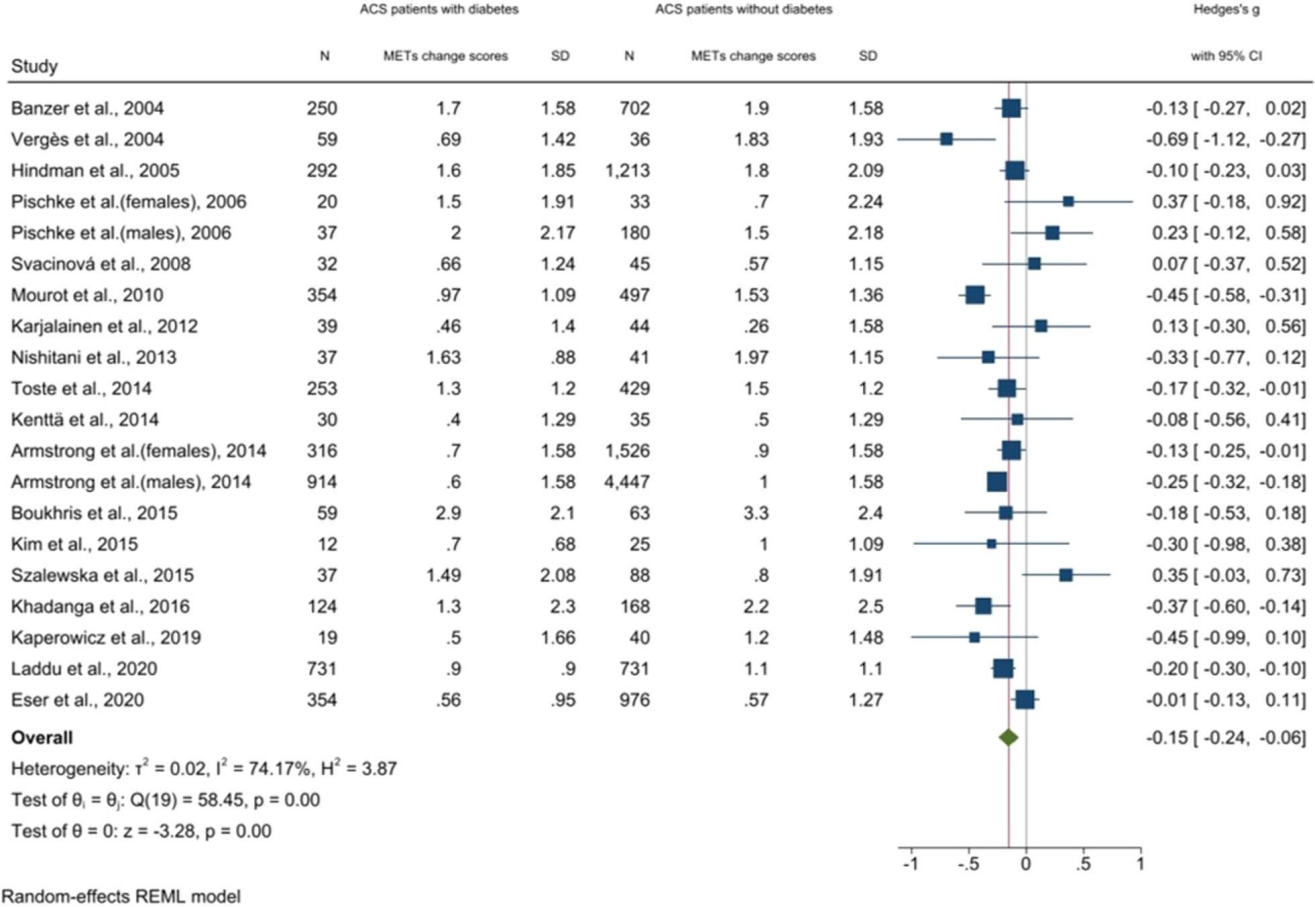
Forest plot: Meta-analysis of changes in exercise capacity (expressed in METs) from the start to the end of CR intervention in ACS patients with a co-diagnosis of diabetes compared to those without

After including n = 15,288 patients from 18 studies [28, 31–47], the comparison showed a significantly smaller change in exercise capacity (METs) in ACS patients with a co-diagnosis of diabetes than in those without (-0.15 (95% CI: -0.24; -0.06) I^2^ = 74%, p < 0.01) (Fig. 3). However, the effect size was considered small (SMD < 0.40) [26]. The sensitivity analysis to evaluate the impact of the SD imputation strategy did not give rise to concern regarding the primary imputation strategy (results not shown). Because only half of the studies used a cardiopulmonary exercise test with direct measures of VO_2,_ which is considered the gold standard for measuring exercise capacity [48], a post hoc sensitivity analysis on the exercise test (direct versus estimated test protocol) was applied and did not show a significant difference in the estimate (p = 0.34).

Narrative synthesis of the two studies excluded for meta-analysis reported comparable benefits of exercise capacity in ACS patients with a co-diagnosis of diabetes compared to those without in one study including n = 28 participants (estimates not reported) [29]. The study with an insufficient test protocol including n = 1,312 participants reported significantly less benefit in exercise capacity in ACS patients with a co-diagnosis of diabetes compared with those without (change in METs: 1.70 (95% CI: 1.50–1.90) vs. 2.50 (95% CI: 2.40–2.70) p < 0.05) [30].

### Comparison of long-term (> 12 months) changes in exercise capacity

**Fig. 4:**
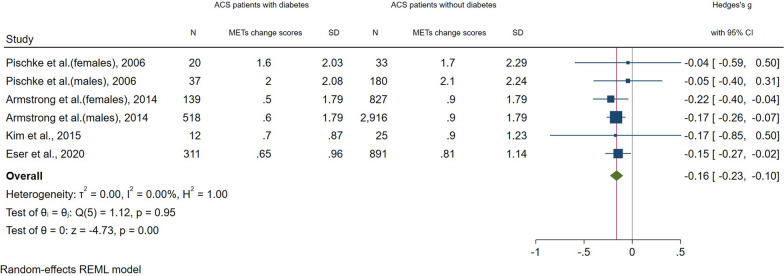
Forest plot: Meta-analysis of changes in exercise capacity (METs) from start of CR intervention to ≥ 12 months follow-up in ACS patients with a co-diagnosis of diabetes compared to those without

After including n = 5,909 patients from four studies [32, 40, 42, 47], the comparison showed a significantly smaller change in exercise capacity (METs) in ACS patients with a co-diagnosis of diabetes compared to those without (-0.16 (95% CI: -0.23; -0.10) I^2^ = 0%, p ≤ 0.01 (Fig. 4)). However, the effect size was considered small (SMD < 0.40) [26].

### Assessment of publication bias

No funnel plot asymmetry (Egger’s test (p = 0.39)) was present for studies reporting on exercise capacity at the end of intervention; hence, this is interpreted as the results not being affected by small study bias (see Additional file 6, Fig. 6.4).

### Subgroup analyses

In the protocolised univariate subgroup analyses, no statistical difference in METs change between groups were found on age (p = 0.39), BACPR score of CR interventions (p = 0.96), type of diabetes (p = 0.48), type of intervention (p = 0.35), and length of follow-up (p = 0.96) (Figures AD 1–3, Additional file 6). It was not possible to conduct subgroup analyses for study design, risk of bias and sex.

### Secondary outcome results

From eight studies, it was possible to conduct a meta-analysis on cardiac mortality, reinfarction, revascularisation, weight and BMI. The three studies [47, 49, 50] reporting on cardiac mortality showed an increased risk of cardiac mortality at the ≥ 12-month follow-up in ACS patients with a co-diagnosis of diabetes compared to those without (OR, 2.16 [95% CI: 1.49–3.13], I2 = 49% p < 0.01). Three studies [47, 49, 50] reporting on reinfarction and revascularisation events showed a comparable risk of reinfarction at the ≥ 12-month follow-up (reinfarction: OR, 0.94 95% CI [0.617, 1.445], I^2^ = 3%, p = 0.79, revascularisation: OR, 1.07 95% CI [0.86,1.45], I^2^ = 19%, p = 0.54). Four studies on weight [30–32, 44] and six studies on BMI [30, 31], 33, 38, 44, 46] showed comparable changes in ACS patients with a co-diagnosis of diabetes compared to those without at the end of CR (weight: 0.20 (95% CI: 0.04; 0.37) I^2^ = 48%, p = 0.10; BMI: 0.19 (95% CI: 0.13; 0.26) I^2^ = 10%, p = 0.27). Additional file 3 provides a narrative description of the secondary outcome results that could not be analysed using meta-analysis.

## Discussion

This systematic review aimed to compare the benefit of CR on exercise capacity and secondary outcomes between ACS patients with a co-diagnosis of diabetes and those without. From 18 observational studies, our findings suggest that compared to ACS patients without diabetes, those with a co-diagnosis of diabetes showed a reduction in benefit on exercise capacity. The magnitude of this difference is, however, considered small. As we found substantial heterogeneity and high levels of risk of bias among the included studies, the results should thus be interpreted with caution. For a more definite conclusion, consistency in methodologies are need with special attention to correct classification of diabetes diagnosis and confounding factors. Exploration of the subgroup analyses including clinical factors (age, type of intervention, type of diabetes), indicated that the observed heterogeneity on the primary outcome was more likely to be explained by methodological heterogeneity rather than clinical heterogeneity.

Our findings on secondary outcomes based on the results from 18 observational studies yielded diverse results; therefore, we cannot determine a definite conclusion as to whether there is evidence for differential benefits of CR on secondary outcomes for ACS patients with a co-diagnosis of diabetes in comparison to those without.

A clinically significant improvement in exercise capacity has been suggested at one MET (with each MET reducing mortality by 12%) [51]. The results from Fig. 3 show that in 11 of the 20 included study populations in the meta-analysis, improvements in exercise capacity reached or exceeded one MET at the end of the study in ACS patients with diabetes. This suggests that although we did identify a statistically significant difference in benefit after CR between patients with and without diabetes, clinically meaningful improvements can be reached for ACS patients with diabetes at the end of intervention. More studies are needed to draw conclusions on a long-term basis.

For the secondary outcomes, synthesising evidence was challenged due to variation, e.g., in choice of outcome, interventions and follow-up time across studies (Additional file 3). We found an increased risk of cardiac mortality for ACS patients with a comorbidity of diabetes compared to those without at the ≥ 12-month follow-up. Regarding reinfarction, revascularisation, weight and BMI changes seemed comparable between the ACS patients with and without diabetes. The results on blood glucose levels were not judged eligible for meta-analysis; however, improvements were not maintained in the long term for ACS patients with diabetes in one study [47]. Assessment of glycaemic control is recommended as a crucial element for optimised CR for ACS patients with diabetes and should be provided as an add-on to CR for these patients combined with strategies to improve long-term adherence to medication and healthy lifestyle to maintain decreases in blood glucose levels from a life-long perspective [12]. Future studies in ACS patients with a co-diagnosis of diabetes should strive to evaluate CR on comprehensive and standardised outcomes reflecting the biopsychosocial nature of CR.

The prognosis for ACS patients with diabetes is reported to be remarkably poor when compared to that for ACS patients without diabetes [6–8]. CR programmes have been reported to be underused, which is a plausible explanation for the insufficient management of ACS patients with diabetes [52]. This possibility is also supported by Jiménez-Navarro et al., who showed that although CR reduced mortality after percutaneous coronary intervention (PCI) for patients with diabetes, CR participation was paradoxically lower in patients with diabetes [53]. Furthermore, a recent study suggests that having diabetes is a strong factor affecting CR uptake [5]. Challenges regarding non-participation in CR for patients with diabetes should be a subject for future studies to identify risk factors for non-attendance to target uptake and intervention to ensure delivery of CR for ACS patients with diabetes.

### Strengths and limitations

This study presents the most comprehensive systematic overview of existing evidence on differences in exercise capacity and secondary outcomes in ACS patients with and without diabetes involved in CR. Several limitations including bias from study designs and diverse methodologies in included studies however, need to be addressed as this might contribute to the vast heterogeneity observed on the primary outcome. Most importantly, included studies failed to control for confounding elements such as differential patients characteristics at baseline. Demographic and clinical covariates such as age, sex, baseline exercise capacity and surgical intervention have been identified as predictors of suboptimal gain in exercise capacity and would be relevant parameters to take into account [54, 55]. In addition to controlling for confounding elements, retrospectively formed study populations made it difficult to assess bias for the selection of participants into the study. Criteria for these study populations were, e.g., exclusion of patients registered with no follow-up exercise test [31, 33, 38, 42] or exclusion of patients who were not able to complete the CR programme [31, 37, 38, 46]. Exclusion of these groups limits the generalisability of the results to ACS patients attending and completing CR. Furthermore, limited information on patients lost to follow-up made it difficult to assess the impact of missing outcomes [35, 36, 47]. In this regard, Pischke et al. [32] reported that patients with diabetes who were lost to follow-up were significantly older and less educated than those with complete follow-up. In this case, patients lost to follow-up might have affected the results of this review and potentially diminished the difference between patients with and without diabetes.

For a pooled effect estimate in the meta-analysis, VO_2_ were converted into METs in five studies [31, 34, 36, 37, 44]. This does not seem to bias the result to a better or worse result, but might give a higher variation in these studies and thus a potential limitation 56].

Several studies did not report systematically screening for diabetes at the beginning of CR [28, 31–33, 38, 40, 43, 45]. As the prevalence of diabetes has previously been found to be considerably underestimated among patients with coronary disease [4], it is likely that misclassification of diabetes diagnosis has occurred. Additionally, diagnostic criteria of diabetes varied across the included studies. This might have contributed to the observed heterogeneity in the results on the primary outcome.

Despite our research question addressing effectiveness, the global implementation of CR as standard care [57] makes it impossible to address this with an RCT design due to ethical issues. Hence, the question naturally calls for observational studies, as confirmed by the included observational studies. The general lack of control groups not receiving CR prevents us from comparing results to the natural disease progression in patients with ACS and diabetes. However, from Kenttä et al. [39], it is indicated that CR itself prevents loss of physical function in patients with diabetes, as a control group not receiving CR was found to have greater loss in physical function [39].

Regarding the risk of bias assessment, we did not find a suitable tool to evaluate the effect of an intervention among different subgroups (ACS patients with a co-diagnosis of diabetes versus those without). The applicability of the ROBINS-E tool for our research question was challenged, as the tool originally was developed for studies examining the effects of environmental exposures on health outcomes [58]. Additionally, ROBINS-E fails to discriminate between studies with a single risk of bias or multiple risks of bias. ROBINS-E is severely limited at determining whether confounders will bias study outcomes [58]. An alternative tool, such the checklist by Wells and colleagues [59], were considered, but the focus on intervention effects was not appropriate for the aim of this review. Nevertheless, we believe that the risk of bias assessment from ROBINS-E (Fig. 2) addressed relevant methodological issues. Until a more suitable risk of bias tool is available, we did not find it relevant to define the quality of evidence according to the Grading of Recommendation, Assessment, Development and Evaluation (GRADE) approach as described in the protocol [60].

### Implications for practice and further research

The findings from this systematic review highlight the need for further high-quality research into the content and effects of CR for patients with diabetes as well as participation over the course of CR for patients with diabetes. Most importantly, future studies should make efforts to eliminate potential confounding parameters such as demographic, behavioural and clinical factors that differ between ACS patients with diabetes and those without. Additionally, when a suitable checklist is available, a formal risk of bias assessment of secondary outcomes should be carried out, and clinical practice should continue to ensure the inclusion of ACS patients with diabetes in CR, as clinically meaningful benefits regarding exercise capacity seem to be reached.

## Conclusion

The benefit of CR on exercise capacity in ACS patients was lower in patients with a co-diagnosis of diabetes than in those without. Given the small magnitude of this difference in exercise capacity together with substantial heterogeneity in the results of the study, further research is needed. Future work should seek to eliminate bias in observational studies, evaluate CR on comprehensive outcomes and investigate participation in CR for patients with diabetes.

## Supplementary Information

**Additional file 1.**Structure of search strategy.

**Additional file 2.** BACPR Standards and Core Components.

**Additional file 3.**. Secondary outcomes.

**Additional file 4.** Exposure measurement methods, classification of diabetes status.

**Additional file 5.** Outcome measurement methods, exercise capacity.

**Additional file 6.** Subgroup analysis.

## Data Availability

The data used and/or analysed during the current study are available from the corresponding author on reasonable request.

## Abbreviations

CR: Cardiac rehabilitation
ACS: Acute coronary syndrome
METs: Metabolic equivalents
HRQoL: Health-related quality of life
SMD: Standardised mean difference
BACPR: British Association for cardiovascular prevention and rehabilitation
CRF: Cardiorespiratory fitness
OR: Odds ratio
CI: Confidence interval
SD: Standard deviation
