# Supplementary material for: Benefits of cardiac rehabilitation following acute coronary syndrome for patients with and without diabetes: a systematic review and meta-analysis
Source: BMC Cardiovasc Disord. 2022 Jun 27;22:295. doi: 10.1186/s12872-022-02723-5 (PMC9237976; doi:10.1186/s12872-022-02723-5)
Supplement: Supplementary file 1 — Additional file 1.Structure of search strategy. [file 12872_2022_2723_MOESM1_ESM.docx]

**Additional files**

*Benefits of cardiac rehabilitation following acute coronary syndrome for patients with and without diabetes: A systematic review and meta-analysis*

Content

[Additional file 1) Structure of search strategy 2](#_Toc95477206)

[Additional file 2) BACPR Standards and Core Components 11](#_Toc95477207)

[Additional file 3) Secondary outcomes 12](#_Toc95477208)

[1.1 Health Related Quality of Life (HRQoL) 12](#_Toc95477209)

[2.1 Cardiovascular related 14](#_Toc95477210)

[2.1.a. Mortality 14](#_Toc95477211)

[2.1.b. Myocardial infarction (MI) 17](#_Toc95477212)

[2.1.c. Revascularisations 19](#_Toc95477213)

[2.1.d. Hospital readmission 20](#_Toc95477214)

[3.1. Diabetes related 21](#_Toc95477215)

[3.1.a Blood glucose level 21](#_Toc95477216)

[3.1.b. Weight 23](#_Toc95477217)

[3.1.c. Body mass index (BMI) 27](#_Toc95477218)

[4.1 Lifestyle related 31](#_Toc95477219)

[4.1.a Smoking status 31](#_Toc95477220)

[4.1.b. Physical activity 33](#_Toc95477221)

[5.1 Well-being 35](#_Toc95477222)

[5.1. Psychological well-being 35](#_Toc95477223)

[6. Work related 36](#_Toc95477224)

[6.1.a Return to work 36](#_Toc95477225)

[References (Additional file 3) 37](#_Toc95477226)

[Additional file 4) Exposure measurement methods, classification of diabetes status 39](#_Toc95477227)

[Additional file 5) Outcome measurement methods, exercise capacity 41](#_Toc95477228)

[Additional file 6) Subgroup analysis 46](#_Toc95477229)

### Additional file 1) Structure of search strategy

| **Relevant aspects of the search** | | |
| --- | --- | --- |
| Population a: | Acute Coronary Syndrome | Blocks combined with AND |
| Population b: | Diabetes Mellitus |  |
| Intervention: | Cardiac Rehabilitation |  |
| Study design: | RCT and Observational studies |  |

| **PubMed searched 26.05.2021** | |
| --- | --- |
| Number of hits: 1719 | |
| **Population a** | |
| Myocardial ischemia[MeSH Terms]  Myocardial ischemia[Title/Abstract]  Myocardial Revascularization[MeSH Terms] Myocardial Revascularization[Title/Abstract]  Percutaneous Coronary Intervention[MeSH Terms]  Percutaneous Coronary Intervention[Title/Abstract]  Acute Coronary Syndrome[Title/Abstract] Coronary Disease[Title/Abstract]  Coronary Artery Disease[Title/Abstract] Coronary Stenosis[Title/Abstract]  Coronary Thrombosis[Title/Abstract]  Myocardial Infarction[Title/Abstract] | Combined with OR |
| **Population b** | |
| Diabetes mellitus[MeSH Terms]  diabetes[Title/Abstract] | Combined with OR |
| **Intervention** | |
| Rehabilitation[MeSH Terms]  cardiac rehabilitation[Title/Abstract]  Secondary Prevention[MeSH Terms]  Tertiary prevention[MeSH Terms]  Exercise[MeSH Terms]  Exercise[Title/Abstract]  physical exertion[MeSH Terms]  Health Promotion [Mesh]  Health Education[MeSH Terms]  Self care[Title/Abstract]  Self-management[Title/Abstract]  patient education[Title/Abstract]  Health Behavior [Mesh]  physical activity[Title/Abstract] | Combined with OR |
| **Study design** | |
| randomized controlled trial[pt]  controlled clinical trial[pt]  randomized[Title/Abstract]  placebo[Title/Abstract]  clinical trials as topic[mesh:noexp]  randomly[Title/Abstract]  non randomized[Title/Abstract]  nonrandomized[Title/Abstract]  follow-up[Title/Abstract]  observational[Title/Abstract]  longitudinal[Title/Abstract]  case control[Title/Abstract]  cohort[Title/Abstract]  prospective[Title/Abstract]  retrospective[Title/Abstract]  Observational Study"[Publication Type] | Combined with OR |
| Filters: Publication date from 2000/01/01 to 2020/05/26 NOT ((((animals[mh] NOT humans[mh])))) | |

| **Embase searched 26.05.2021** | |
| --- | --- |
| Number of hits: 2376 | |
| **Population a** | |
| 'percutaneous coronary intervention'/exp  'coronary artery surgery'/exp  'ischemic heart disease'/exp  'coronary artery disease':ab,ti  'coronary stenosis':ab,ti  'coronary thrombosis':ab,ti | Combined with OR |
| **Population b** | |
| 'diabetes mellitus'/exp  'diabetes':ab,ti | Combined with OR |
| **Intervention** | |
| 'rehabilitation'/exp  'cardiac rehabilitation':ab,ti  'health behavior'/exp  'exercise'/exp  'health education'/exp  'secondary prevention'/exp  'tertiary prevention'/exp  'kinesiotherapy'/exp  'self care'/exp | Combined with OR |
| **Study design** | |
| 'randomized controlled trial'/exp  random:ab,ti  factorial:ab,ti  'crossover':ab,ti  'cross over':ab,ti  'cross-over':ab,ti  placebo:ab,ti  doubl:ab,ti  allocat:ab,ti  assign:ab,ti  volunteer:ab,ti  'case control':ab,ti  observational':ab,ti  retrospect:ab,ti  prospecti:ab,ti  cohort:ab,ti  'pragmatic trial':ab,ti  'clinical trial':ab,ti  observational study'/exp | Combined with OR |
| **Limits:** | |
| Publication year 2000-2020: [2000-2020]/py  Article types: NOT ('article'/it OR 'article in press'/it OR 'review'/it) AND ('interview'/de OR 'practice guideline'/de) | |

| **CENTRAL** **last searched 26.05.2021** | |
| --- | --- |
| Number of hits: 627 | |
| **Population a** | |
| MeSH descriptor: [Myocardial Ischemia] explode all trees  MeSH descriptor: [Myocardial Revascularization] explode all trees  MeSH descriptor: [Percutaneous Coronary Intervention] explode all trees  "Coronary Artery Disease":ti,ab,kw  "Coronary Stenosis":ti,ab,kw  "Coronary Thrombosis":ti,ab,kw | Combined with OR |
| **Population b** | |
| MeSH descriptor: [Diabetes Mellitus] explode all trees  diabetes:ti,ab,kw | Combined with OR |
| **Intervention** | |
| MeSH descriptor: [Rehabilitation] explode all trees  ("patient education"):ti,ab,kw  ("physical activity"):ti,ab,kw  (exercise):ti,ab,kw  ("cardiac rehabilitation"):ti,ab,kw  MeSH descriptor: [Physical Exertion] explode all trees  MeSH descriptor: [Exercise] explode all trees  MeSH descriptor: [Health Education] explode all trees  MeSH descriptor: [Tertiary Prevention] explode all trees  MeSH descriptor: [Secondary Prevention] explode all trees | Combined with OR |
| **Study design** | |
| None | |
| **No limits** | |

| **CINAHL searched 26.05.2021** | |
| --- | --- |
| Number of hits: 714 | |
| **Population a** | |
| (MH "Myocardial Ischemia+")  (MH "Coronary Disease+")  (MH "Myocardial Infarction+")  (MH "Myocardial Revascularization+")  (MH "Percutaneous Coronary Intervention"  “TI ( Coronary Artery Disease OR Coronary Stenosis OR Coronary Thrombosis ) OR AB ( Coronary Artery Disease OR Coronary Stenosis OR Coronary Thrombosis )” | Combined with OR |
| **Population b** | |
| (MH "Diabetes Mellitus+")  ”TI diabetes OR AB diabetes” | Combined with OR |
| **Intervention** | |
| (MH "Rehabilitation+")  (MH "Self Care+")  (MH "Therapeutic Exercise+")  (MH "Tertiary Health Care") OR (MH "Secondary Health Care")  (MH "Health Education+")  AB "Health Promotion" OR TI "Health Promotion"  (MH "Exercise+")  AB "physical exertion" OR TI "physical exertion"  (MH "Health Behavior+")  TI "cardiac rehabilitation" OR AB "cardiac rehabilitation"  AB exercise OR TI exercise  AB "patient education" OR TI "patient education"  TI "physical activity" OR AB "physical activity" | Combined with OR |
| **Study design** | |
| (MH "Randomized Controlled Trials+")  (MH "Clinical Trials+")  (MH "Nonexperimental Studies+")  AB ( randomized OR placebo OR "clinical trial" OR randomly OR "non randomized" OR nonrandomized OR "follow-up" OR observational OR longitudinal OR "case control" OR cohort OR prospective OR retrospective ) OR TI ( randomized OR placebo OR "clinical trial" OR randomly OR "non randomized" OR nonrandomized OR "follow-up" OR observational OR longitudinal OR "case control" OR cohort OR prospective OR retrospective ) | Combined with OR |
| **Limits:** | |
| Published Date: 20000101-20200526 | |

| **Web of Science last searched 26.05.2021** | |
| --- | --- |
| Number of hits: 1275 | |
| **Population a** | |
| \|  \| TS=("Myocardial Ischemia") OR TS=("Myocardial Revascularization") OR TS=("Acute Coronary Syndrome" OR "Angina Pectoris" OR "Coronary Disease" OR "Myocardial Infarction") OR TS=("Percutaneous Coronary Intervention") OR TS=("Coronary Artery Disease" OR "Coronary Stenosis" OR "Coronary Thrombosis") \| \| --- \| --- \| | Combined with OR |
| **Population b** | |
| TOPIC: (diabetes)  TOPIC: ("Diabetes Mellitus") | Combined with OR |
| **Intervention** | |
| **TOPIC:** (Rehabilitation OR "Cardiac rehabilitation" OR "Self Care" OR "Self-management" OR "Exercise therapy " OR "Secondary Prevention" OR "tertiary prevention" OR "Health Education" OR "Patient Education" OR "Health Promotion" OR Exercise OR "physical exertion" OR "Health Behavior" OR "physical activity") | Combined with OR |
| **Study design** | |
| ("randomized controlled trial" OR "controlled clinical trial" OR randomized OR placebo OR "clinical trial" OR randomly OR "non randomized" OR nonrandomized OR "follow-up" OR observational OR longitudinal OR "case control" OR cohort OR prospective OR retrospective) | Combined with OR |
| **Limits:** | |
| (ARTICLE) | |

| **Clinicaltrials.gov**  **last searched 01.10.2019**  Search 1:  rehabilitation AND "heart disease" AND diabetes  Number of hits= 4  Search 2:  exercise AND "heart disease" AND diabetes  Number of hits=35 |
| --- |

### Additional file 2) BACPR Standards and Core Components

| **BACPR Standards and Core Components ^15^** | **Health behaviour change and education** | **Lifestyle risk factor** | **Psychosocial health** | **Medical risk management** | **Long-term strategies** | **Audit and evaluation** | **Score** |
| --- | --- | --- | --- | --- | --- | --- | --- |
| **Banzer et al, 2003, USA** | 1 | 1 | 1 | 1 | 0 | 1 | 5 |
| **Vergès et al 2003, France** | 1 | 1 | 0 | 1 | 0 | 1 | 4 |
| **Hindman et al., 2005, USA** | 1 | 1 | 1 | 1 | 0 | 1 | 5 |
| **Pischke et al 2006, USA** | 1 | 1 | 1 | 0 | 1 | 1 | 5 |
| **Svacinová et al 2008 Czech Republic** | 0 | 1 | 0 | 0 | 0 | 1 | 2 |
| **Mourot et al 2010, France** | 1 | 1 | 0 | 1 | 0 | 1 | 4 |
| **Karjalainen et al 2012, Finland** | 0 | 1 | 0 | 0 | 0 | 1 | 2 |
| **Nishitani et al 2013 Japan** | 1 | 1 | 0 | 0 | 0 | 1 | 3 |
| **Toste et al 2013, Portugal** | 1 | 1 | 1 | 0 | 0 | 1 | 4 |
| **Armstrong et, al, 2014, Canada** | 1 | 1 | 1 | 1 | 0 | 1 | 5 |
| **Kenttä et al 2014, Finland** | 0 | 1 | 0 | 0 | 0 | 1 | 2 |
| **Kim et al, 2015, Korea** | 1 | 1 | 0 | 1 | 1 | 1 | 5 |
| **Szalewska et al 2015 Poland** | 1 | 1 | 0 | 0 | 0 | 1 | 3 |
| **Boukhris et al., 2015, Italy** | 0 | 1 | 1 | 0 | 0 | 1 | 3 |
| **Khadanga et al, 2017 USA** | 1 | 1 | 1 | 0 | 1 | 1 | 5 |
| **Kasperowicz et al., 2019 , Poland** | 0 | 1 | 0 | 0 | 0 | 1 | 2 |
| **Laddu et al. 2020, Canada** | 1 | 1 | 0 | 1 | 0 | 1 | 4 |
| **Eser et al., 2020, Switzerland** | 1 | 1 | 0 | 0 | 0 | 1 | 3 |
| **total** | 13 | 18 | 7 | 7 | 3 | 18 |  |

### Additional file 3) Secondary outcomes

1.1 Health Related Quality of Life (HRQoL)

**Study characteristics and participants**

The review identified two studies reporting on HRQoL^1, 2^. Both studies were observational cohort studies.

The percentage of patients with diabetes ranged from 20.7%- 28.2 % in the study populations. St. Clair did not specify the type of diabetes ^1^, while Pischke et al. included patients with type 1 or type 2 diabetes (9.8 % reported with type 1 diabetes) ^2^.

In total HRQoL was analysed in total n=970 patients of these, n=461 had diabetes.

**Interventions**

CR programs were delivered as an outpatient service with patients being enrolled after discharge from surgical procedure. Both interventions were provided at hospital or medical center as a 12-week program with three sessions pr. week. Besides exercise sessions, health and nutrition education sessions were provided. In Pischke et al., stress management was facilitated and patients continued to meet in groups weekly for the next 40 weeks after the intervention for long term adherence ^2^.

**Outcome**

HRQoL were measured with two different self-reported questionnaires. St. Clair et.al. used the disease specific Ferrans and Powers Quality of Life Index Cardiac version IV questionnaire at baseline and at 12 weeks ^3^. Pischke et al. used the generic Medical Outcomes Study shortform health survey (MOS SF-36) at baseline, three months and 12 months and computed two aggregated scores on physical and mental components.

**Synthesis of results**

Due to heterogeneity in outcome measures (generic and disease specific questionnaires) pooling of the results was not judged eligible. Between group comparison showed comparable changes in HRQoL in both studies at 12-weeks follow-up ^1, 2^. In Pischke et al. ^2^ comparable changes in HRQoL remained at one-year follow-up.

| Table 1.1 Descriptive results on Health Related Quality of Life (HRQoL) | | | | | | | | |
| --- | --- | --- | --- | --- | --- | --- | --- | --- |
| **Study** | **Patients with diabetes** | | | | **Patients without diabetes** | | | **Between group comparison** |
| St. Clair et al. ^1^ | Mean change, 95 % CI: 1.9 (1.4-2.4)^[[1]](#footnote-1)^ | | | | Mean change, 95 % CI: 1.8 (1.5-2.1)^1^ | | | Comparable changes in HRQoL between groups at 12 weeks follow up (p value not significant) |
| Pischke et al. ^2^ | Physical health^[[2]](#footnote-2)^ | Baseline (SD) | 3 months (SD) | 12 months (SD) | Baseline (SD) | 3 months (SD) | 12 months (SD) | Comparable changes in HRQoL between groups at 12 weeks and one year follow up |
|  | Men | 46.5±12.1 | 50.3± 9.2 | 51.8±10.1 | 48.4±10.3 | 52.9±8.8 | 52.1± 9.7 |  |
|  | Women | 40.0±10.1 | 47.4±10.5 | 52.0±9.2 | 47.5±9.7 | 54.4±8.9 | 52.0±9.2 |  |
| Pischke et al. ^2^ | Mental health^2^ |  |  |  |  |  |  | Comparable changes in HRQoL between groups at 12 weeks and one year follow up |
|  | Men | 46.5±12.1 | 50.3±9.2b | 51.8±10.1 | 48.4±10.3 | 52.9±8.8 | 52.1±9.7 |  |
|  | Women | 40.0±10.1 | 47.4±10.5 | 46.2±12.5 | 47.5±9.7 | 54.4±8.9 | 52.0±9.2 |  |

### 2.1 Cardiovascular related

2.1.a. Mortality

The review identified five studies reporting on mortality. All studies were all observational cohort studies. The reported percentage of patients with diabetes ranged from 12.4-32.0% in the enrolled study populations. Two studies included patients with type 1 or type 2 diabetes ^4, 5^. The type of diabetes was not specified in three studies ^6-8^. In total, mortality was analysed in n= 5932 patients of these, n= 1100 had diabetes.

**Interventions**

CR programs were an outpatient service in two studies ^4, 7^. Reibis et al. provided CR as an inpatient program. In Yu et al. the intervention was delivered in three phases: inpatient, outpatient and homebased intervention with follow up visits to monitor progress ^8^. Eser et al. was a multicentre study provided CR as both in- and outpatient programme ^5^. Patients were in all studies enrolled after discharge from surgical procedure. Interventions were in all studies provided at hospital or a rehabilitation centre. CR was provided as a three-week program in Reibis et al. ^6^, a six-week program in Suresh et.al. ^7^, and a 12-months program in Yu et a l^8^ and Eser et al., provided CR as three weeks to three months programme ^5^. In Giallauria et al., the duration of the program was not reported ^4^.Two studies provided two sessions per week ^7, 9^. Eser et al., provided in 10-36 sessions in total ^5^ Two studies did not report the number of sessions ^4, 6^. Besides exercise sessions, all four studies provided educational sessions concerning nutrional counselling and risk factor control. Additionally, Yu et al. offered vocational guidance ^8^.

**Outcome**

Mortality was reported as over-all mortality and/or cardiac mortality. Follow up was performed at 14 days in Giallauria et al. ^4^, 12 months in Suresh et al. and Eser et al. ^5, 7^, 13.4 months in Reibis et al. ^6^, and 3.2 years in Yu et al. ^8^.

**Synthesis of results**

Due to heterogeneity in follow-up time and reporting on over-all mortality pooling of the results was only judged eligible in three studies measuring cardiac mortality at one year ^5-7^.

*Results from the meta-analysis (cardiac mortality)*

The meta-analysis based on the three studies ^5-7^ showed a statistically significant increased risk of cardiac mortality at one year in patients with diabetes (OR, 2.16 [95% CI: 1.49-3.13]; I^2^= 48.9 %, p=0.00). The may represent moderate heterogeneity. Test of funnel plot asymmetry was not performed due to inclusion of less than <10 studies in the meta-analysis ^10^.

*Results from studies not included in the meta-analysis (over-all mortality)*

At 14-days follow-up, one study found comparable mortality between groups ^4^. At one-year follow up, two studies found comparable mortality ^5, 6^ whereas one study found higher mortality at one-year among patients with diabetes ^7^. At three-years follow-up, one study found mortality higher in patients with diabetes ^8^.

| **Table 2.1.a. Descriptive results on cardiac- and over-all mortality** | | | |
| --- | --- | --- | --- |
| **Study** | Patients with diabetes | **Patients without diabetes** | **Between group comparison** |
| Giallauria et al. ^4^ | Overall mortality: 1% | Overall mortality: 0.5% | Over-all mortality was comparable between groups at 14 days follow up (p=0.23) |
| Reibis et al.^6^ | Cardiac mortality: 1.2 % | Cardiac mortality: 1.5 % | Over-all and cardiac mortality were comparable between groups at one year.  Overall mortality:  Adjusted odds ratio (95 % CI):  0.37 (0.07-2.02) (p=0.252)  Mortality due to cardiovascular events (p=0.874) |
| Suresh et al. ^7^ | Over-all mortality:15.7 %  Cardiac mortality: 13.4 % | Over-all mortality:5.6 %  Cardiac mortality: 5.4 % | Over-all and cardiac mortality was statistically significant higher in patients with diabetes at one year follow up.    Risk difference (95 % CI):  Over-all mortality: 0.10 (0.05-0.15)  Cardiac mortality: 0.08 (1.69-3.70) |
| Yu et al. ^8^ | Over-all mortality: 23.4 % | Over-all mortality: 5.5 % | Over-all mortality was statistical significantly higher in patients with diabetes at three year follow up  Relative Risk:  4.3, p=0.03 |
| Eser et al. ^5^ | Over-all mortality:2.3 %  Cardiac mortality: 2.3 % | Over-all mortality: 1.2 %  Cardiac mortality: 0.6 % | Overall mortality was comparable between groups at one year follow up (p=0.209) |
|  |  |  | Cardiac mortality was statistically significant higher in patients with diabetes at one year follow up (p=0.009) |

Figure 2.1.a. Meta-analysis on cardiac mortality

2.1.b. Myocardial infarction (MI)

The review identified three observational studies reporting on myocardical infarction (MI) ^5-7^. The reported percentage of patients with diabetes ranged from 12.4-26.4 % in the enrolled study populations. The type of diabetes was not specified in two of the studies ^6, 7^. Eser et al. included type 1 and type 2 diabetes. In total, MI was analysed in n= 4697 patients of these, n= 884 had diabetes.

**Interventions**

Reibis et al. provided CR as an inpatient program, whereas Suresh et al. was an outpatient program^6, 7^. Eser et al. was a multicentre study provided CR as both in- and outpatient programme ^5^. Patients were enrolled after discharge from surgical procedure. Interventions were provided at hospital or a rehabilitation centre. CR was provided as a three-week program in Reibis et al. ^6^, a six week program in Suresh et.al. ^7^ and Eser provided CR as three weeks to three months programme ^5^. Suresh et al. provided two sessions per week while Reibis et al. did not report the number of sessions per week ^6, 7^. Eser et al., provided in 10-36 sessions in total ^5^. Besides exercise sessions, all studies provided educational sessions concerning nutrional counselling and risk factor control.

**Outcome**

Follow up was performed at 12 months in Suresh et al. and Eser et.al. ^5, 7^ and 13.4 months in Reibis et al. ^6^.

**Synthesis of results**

*Results from the meta-analysis*

| **Table 2.1.b. Descriptive results on myocardial infarction (MI)** | | | |
| --- | --- | --- | --- |
| **Study** | **Patients with diabetes** | **Patients without diabetes** | **Comparison of patients with diabetes versus patients without diabetes** |
| Reibis et al.^6^ | 2.9 % | 2.4 % | Comparable risk of myocardial infarction between groups at one year follow up (p=0.646) |
| Suresh et al. ^7^ | 4.5% | 6.5 % | Comparable risk of reinfarction between groups at one year follow up  (risk difference: -0.02 95 % CI [-0.05-0.01]; p=0.24) |
| Eser et al. ^5^ | 2.3 % | 1.8 % | Comparable risk of acute myocardial infarction between groups at one year follow up (p=0.325) |

Pooling of results from the three studies showed comparable risk of myocardial infarction between groups at one-year follow-up (OR, 0.94 95% CI [0.62, 1.45]; I^2^= 2.8 % p= 0.792). Results may not be affected by heterogeneity. Test of funnel plot asymmetry was not performed due to inclusion of less than <10 studies in the meta-analysis ^10^.

Figure 2.1.b. Meta-analysis on myocardial infarction (MI)

2.1.c. Revascularisations

Same study characteristics and interventions as 2.1.c.

**Outcome**

Revascularisation was reported as coronary bypass artery grafting (CABG) or percutaneous coronary intervention (PCI/PTCA) and cardiac intervention (PCI or CABG).

Follow up was performed at 12 months in Suresh et al. and Eser et.al. ^5, 7^ and 13.4 months in Reibis et al. ^6^.

| **Table 2.1.c. Descriptive results on revascularisations** | | | |
| --- | --- | --- | --- |
| Study | Patients with diabetes | Patients without diabetes | Comparison of patients with diabetes versus patients without diabetes |
| Reibis et al.^6^ | PCI: 14.0 %,  CABG: 1.7% | PCI: 13.1 %,  CABG: 1.8 % | Comparable risk of PCI/CABG between groups at one year follow up  (PCI p=0.67)  (CABG p=0.646) |
| Suresh et al. ^7^ | PTCA: 0.9 %,  CABG: 12.6 % | PTCA: 2.5 %,  CABG: 12.7 % | Comparable risk of PCI/CABG between groups at one year follow up  (risk difference: -0.01 95 % CI [-0.06-0.03]; p=0.6) |
| Eser et al. ^5^ | PCI or CABG: 9.9 % | PCI or CABG: 8.1 % | Comparable risk of cardiac intervention between groups at one year follow up  (p=0.325) |

*Results from the meta-analysis*

Pooling of results from the three studies showed comparable risk of revascularisation between groups at one-year follow-up (OR, 1.07 95 % CI [0.86,1.45]; I^2^=18.6 % p=0.540). Results may not be affected by heterogeneity. Test of funnel plot asymmetry was not performed due to inclusion of less than <10 studies in the meta-analysis ^10^.

Figure 2.1.c. Meta-analysis on revascularisations

2.1.d. Hospital readmission

The review identified two studies reporting on hospital readmission ^6, 9^. Eser et al., reported hospitalisation for cardiac reason ^5^. All studies were all observational cohort studies. The reported percentage of patients with diabetes ranged from 16.6-32% in the enrolled study populations. Eser et al., included patients with type 1 and type 2 diabetes ^5^. The type of diabetes was not specified in two of the studies ^6, 9^.

In total, hospital readmission was analysed in n= 3496 patients of these, n= 795 had diabetes.

**Interventions**

Reibis et al. provided CR as an inpatient program, whereas Yu et al. was an combined in- and outpatient program with homebased follow up. Eser et al. was a multicentre study provided CR as both in- or outpatient programme ^5^. Patients were enrolled after discharge from surgical procedure. Interventions were provided at hospital or a rehabilitation centre. CR was provided as a three week program in Reibis et al. ^6^, a six months program with maintenance phone calls in Yu et.al. for up to a year ^9^ and Eser provided CR as three weeks to three months programme ^5^.Yu et al. provided two sessions per week ^8^. Eser et al., provided in 10-36 sessions in total ^5^. Reibis et al. did not report the number of sessions per week ^6^. Besides exercise sessions, all studies provided educational sessions concerning nutrional counselling and risk factor control.

**Outcome**

One study reported all cause readmission ^6^, whereas two studies reported readmission due to cardiac reasons ^5, 8^. Mean follow up was 13.4 months in Reibis et al. ^6^. Mean follow up in Yu et al. was 3.2±1.1 years ^9^. Follow up in Eser et al. was 12 months ^5^.

**Synthesis of results**

Due to heterogeneity in outcome and follow-up time, meta-analysis on readmission were not judged eligible. Comparable risk of readmission at one year were found in two studies ^5, 6^ whereas one study showed higher risk of readmission for patients with diabetes at three years follow-up ^9^

| Table 2.1.d. Descriptive results on hospital readmission | | | |
| --- | --- | --- | --- |
| Study | Patients with diabetes | Patients without diabetes | Between-group comparison |
| Reibis et al.^6^ | 21.1 % | 19.8 % | Comparable risk of all cause readmission between groups at one year follow up (p=0.428) |
| Yu et al. ^9^ | 32% | 23% | Patients with diabetes had a higher risk of cardiac related readmission compared to patients without diabetes at three years follow up  (Relative risk: 4.3 p=0.03) |
| Eser et al. ^5^ | 10.9% | 7.9 % | Comparable risk of cardiac related readmission between groups at one year follow up (p= 0.089) |

### 3.1. Diabetes related

3.1.a Blood glucose level

**Study characteristics and participants**

The review identified four observational ^1, 5, 11, 12^ and one controlled cohorte study reporting on blood glucose level ^13^.

The reported percentage of patients with diabetes ranged from 19.4 %- 28.2 % in the enrolled populations. In two studies, only patients with type 2 diabetes were included ^12, 13^. One study included type 1 and type 2 diabetes ^5^. The type of diabetes was not specified in two studies ^1, 11^. In total, blood glucose was analysed in n=4095 patients of these, n=1079 had diabetes.

**Interventions**

CR programs were delivered as an outpatient service with patients being enrolled after discharge from surgical procedure. Interventions were in three studies provided at hospital or a medical center ^1, 11, 12^. Eser et al. was a multicentre study provided CR as both in- and outpatient programme ^5^. In Karjalainen et al., the exercise sessions were structured home based sessions ^13^. CR was provided as a 12 week program with three sessions pr. week in two studies ^1, 11^. In Nishitani et al. and Karjalainen et al., interventions lasted six and nine months respectively. Eser et al., provided in 10-36 sessions in programmes lasting three weeks- three months ^5^ Besides exercise sessions, four studies provided educational sessions concerning nutrional and risk factor control ^1, 5, 11, 12^.

**Outcome measurement**

Blood glucose was measured by fasting blood glucose in three studies ^11-13^. Eser et al., were non-fasting ^5^. St. Clair et al. did not specify the measurement method ^1^. Three studies measured blood glucose at baseline and at 12 weeks follow up ^1, 11^. Nishitani et al. and Karjalainen et al measured at baseline, and at 6 months and 24 months respectively. Eser et al. measured at end of CR and at 12 months ^5^.

**Synthesis of results**

Due to heterogeneity in measurement and reporting meta-analysis was not performed. We limited the descriptive results to within group comparison in patients with diabetes. Two studies reported significantly decreases in blood glucose levels at 12 weeks/end of CR ^1, 5^, however an increase was found at one-year in Eser et al. ^5^. No significant changes were found in three studies at 12-weeks, six-months and two-year follow-up ^11-13^.

| **Table 3.1.a. Descriptive results on blood glucose level** | | | |
| --- | --- | --- | --- |
| **Study** | **Patients with diabetes** | **Patients without diabetes** | **Within-group comparison (patients with diabetes)** |
| St. Clair et al. ^1^ | Mean change (95 %CI):  1.2 mg/dl (−4.6 to 6.9)^[[3]](#footnote-3)^  Mean change (95% CI):  −0.5 (−0.8 to −0.3)^[[4]](#footnote-4)^ | Mean change (95% CI):  0.1 (−0.1 to 0.2)^3^  Mean change (95% CI):  −0.5 mg/dl (−2.1 to 1.2)^4^ | Patients with diabetes significantly decreased glycated hemoglobin at 12 weeks follow-up. |
| Karjalainen et al. ^13^ | Baseline: 6.6 ± 0.8^[[5]](#footnote-5)^  Follow up: 6.5 ± 1.0^5^ | Baseline: 5.9 ± 0.4^5^  Follow up: 5.7 ± 0.3^5^ | No significant decrease in blood glucose levels in patients with diabetes at two years follow-up. |
| Hindman et al. ^11^ | Baseline: 142.2 ±48.8^3^  Follow-up: 136.9± 46.1^3^  (3.7 % improvement) | Baseline: 101.4± 24.5^3^  Follow-up: 99.7±17.5^3^  (1.7 % improvement) | No significant decrease in blood glucose levels in patients with diabetes at 12 weeks follow-up (p=0.05) |
| Nishitani et al. ^12^ | Baseline:  143±57 ^3^,  7.0±1.3^5^  Follow-up:  167±68^3^,  7.2±1.4^5^ | Baseline:  103±14^3^,  5.1±0.4%^5^  Follow-up:  112±20^3^  5.2±0.5^5^ | No significant decrease in blood glucose levels in patients with diabetes at six-months weeks follow-up (p>0.05) |
| Eser et al. ^5^ | Median change (baseline to end of CR:  − 1.0^[[6]](#footnote-6)^  Mean change (end of CR to one year:  1.1 mmol/mol | Median change baseline-end of CR:  0.1 mmol/mol  Mean change baseline-one year:  Remained stable | Changes in blood glucose levels were significantly better in patients with diabetes at end of CR, however increased at one-year follow-up. |
|  |  |  |  |

### 3.1.b. Weight

**Study characteristics and participants**

The review identified seven studies reporting on weight ^1, 2, 12, 14-17^. All studies were all observational cohort studies.

The reported percentage of patients with diabetes ranged from 11.5-41.6 % in the enrolled study populations. In three studies, only patients with type 2 diabetes were included ^12, 16, 17^. Pischke et al. included patients with type 1 or type 2 diabetes (9.8 % reported with type 1 diabetes). The type of diabetes was not specified in three studies ^1, 14^. In total, weight was analysed in n=2549 patients of these, n=1376 had diabetes.

**Interventions**

CR programs were delivered as an outpatient service with patients being enrolled after discharge from surgical procedure. Interventions were in three studies provided at hospital or a medical center. CR was provided as a 10-12 week program with three sessions pr. week in six studies ^1, 2, 14-17^. In Nishitani et al. and Carroll et al., interventions lasted six and 15 months respectively. Besides exercise sessions, seven studies provided educational sessions concerning nutrional and risk factor control ^1, 2, 12, 14-16^. Additionally, Khadanga et. al. offered a behavioural weight loss programme for patients with BMI >30 ^16^. Svacinova et.al. offered exercise sessions only ^17^.

**Outcome measurement**

In Banzer et al. weight was only reported for patients with a BMI of ≥30. Weight was reported in pounds (lbs) in two studies ^1, 14^ and kilograms (kg) in five studies ^2, 12, 15-17^. All studies measured weight at baseline. Five studies measured weight at 10-12 weeks follow up ^1, 2, 14, 16, 17^. Nishitani et al. and Carroll et al. measured weight at six and 15 months follow up ^12, 15^

***Results***

*Meta-analysis on weight*

Based on four studies, the overall difference in change scores between groups was 0.20 (95 % CI: 0.04; 0.37) I^2^= 48.32 %, p= 0.10, indicating comparable weight loss in patients with diabetes compared to patients without diabetes. According to the I^2^ statistics, the result might be affected by moderate heterogeneity. Stratification on intervention type (Figure 3.1.b) showed statistical significant group difference (p=0.03) with exercise only resulting in bigger weight change in patients without diabetes. Test of funnel plot asymmetry was not performed due to inclusion of less than <10 studies in the meta-analysis ^10^.

*Descriptive results from studies excluded from meta-analysis*

Regarding the three remaining studies neither of the studies reported between group comparison and showed mixed results; one study reported significant weight loss in both groups ^15^, in another study no weight loss was seen in patients with diabetes, oppositely, weight loss was seen in patients without diabetes ^14^. In the third study, average body weight was very low and weight loss was not considered relevant ^12^.

| **Table 3.1.b. Descriptive results on weight** | | | |
| --- | --- | --- | --- |
| Author | **Patients with diabetes** | Patients without diabetes | **Between-group comparison** |
| Banzer et al.^14^ | Baseline, kg:  107.0  12-week follow-up, kg:  107.0 | Baseline, kg:  98.4  12-week follow-up, kg:  96.2 | Weight change was only reported in patients with BMI≥30 (n=184):  No weight change among patients with diabetes, however a significant weight change in patients without was observed after CR. No between group comparison.  Excluded in meta-analysis due to lack of reporting of n in groups. |
| St. Clair et al. ^1^ | Baseline, kg (±SD):  93.0 ±20.9  12-week, follow-up kg:  92.0 | Baseline, kg (±SD):  84.8 ±18.1  12-week follow-up, kg:  83.5 | Patients with diabetes reduced weight to a lesser extent when compared with patients without diabetes however not statistically significant. |
| Carroll et al. ^15^ | Baseline, kg (±SD)  83.4 ±11.3  15-month follow-up, kg (±SD):  81.7(±10.1) | Baseline, kg (±SD)  79.0 ±12.0  15-month follow-up, kg (±SD):  78.8 (±12.3) | Patient with and without diabetes both reduced weight significantly after 15 months. No between group comparison. Excluded in meta-analysis due to follow-up time. |
| Khadanga et al. ^16^ | Baseline, kg (±SD)  90.4 ±16.7  12 to 16-week follow-up, kg (±SD):  89.4 (±16.7) | Baseline, kg (±SD)  83.7 ±16.7  12 to 16-week follow-up, kg (±SD):  81.4 (±16.1)  (no insulin resistance group) | Weight loss was higher among patients without diabetes compared to patients with diabetes (p=0.04) |
| Nishitani et al.^12^ | Baseline, kg (±SD)  48.4±9.8  6-month follow-up, kg (±SD)  45.2 ±5.2 | Baseline, kg (±SD)  49.4±7.7  6-month follow-up, kg (±SD)  49.6 ±7.3 | Weight loss tended to be higher among patients with diabetes compared to patients without diabetes. No between group comparison.  Excluded in meta-analysis due to low average body weight (Asian population) |
| Pischke et al. ^2^  Men | Baseline, kg (±SD)  95.5±18  12-week follow-up, kg (±SD)  90.9±16.7  12-month follow-up, kg (±SD)  89.9±15.6 | Baseline, kg (±SD)  85.7±15.8  12-week follow-up, kg (±SD)  81.2 ±13.3  12-month follow-up, kg (±SD)  81.0±13.1 | Significant weight change over time in both groups (no between-group comparison) |
| Pischke et al. ^2^,  Women | Baseline, ±kg (±SD)  80.3±17.4  12-week follow-up, kg (±SD)  76.4±16.3  12-month follow-up, kg (±SD)  75.2±15.5 | Baseline, kg (±SD)  75.5±17.7  12-week follow-up, kg (±SD)  70.6±16.2  12-month follow-up, kg (±SD)  69.7±16.3 | Significant weight change over time in both groups (no between-group comparison) |
| Svacinová et al. ^17^, | Baseline, kg (±SD)  86.1±13.4  12-month follow-up, kg (±SD):  86.0±12.5 | Baseline, kg (±SD)  81.3±11.5  12-month follow-up, kg (±SD):  79.7±11.00 | Patients with diabetes did not reach a significant weight loss, patients without diabetes reached significant weight loss. No comparison between groups. |

Figure 3.1.b. Meta-analysis on weight end of Cardiac Rehabilitation (stratified on intervention type)

3.1.c. Body mass index (BMI)

**Study characteristics and participants**

The review identified ten studies reporting on BMI. All studies were all observational cohort studies. The reported percentage of patients with diabetes ranged from 11.5-41.6% in the enrolled study population. In five studies, only patients with type 2 diabetes were included ^12, 16-19^. Two studies included patients with type 1 or type 2 diabetes ^5, 15^. The type of diabetes was not specified in three studies ^1, 11, 20^. In total, BMI was analysed in n=7515 patients of these, n=2424 had diabetes.

**Interventions**

CR programs were an outpatient service except for Gondoni et al where the programme was delivered as an inpatient programme ^20^. Patients were in all studies enrolled after discharge from surgical procedure. Interventions were in all studies provided at hospital or a medical center. Eser et al. was a multicentre study provided CR as both in- and outpatient programme ^5^. CR was provided as an 8-12 week program with three sessions pr. week in five studies ^1, 11, 16, 17, 19, 21^. The inpatient program in Gondoni et al. lasted on average 23 days with six sessions per week. In Nishitani et al., the intervention lasted six months with 1-2 sessions per week ^12^. In Carroll et al., the intervention lasted 15 months with three sessions per week ^15^. Eser et al., provided in 10-36 sessions in programmes lasting three weeks- three months ^5^. Besides exercise sessions, seven studies provided educational sessions concerning nutrional counselling and risk factor control ^1, 5, 11, 12, 15, 16, 20, 21^. Additionally, Khadanga et. al. offered a behavioural weight loss programme for patients with BMI >30 ^16^.

**Outcome measurement**

BMI was in all nine studies calculated as kg/m^2^. All studies measured BMI at baseline. Seven studies measured BMI at 8-12 weeks follow up ^1, 5, 11, 16-18, 21^. Nishitani et al. measured BMI at six months follow up ^12^, Eser at 12 months follow up ^5^, and Carroll et al. at 15 months follow up ^15^. In Gondoni et al., follow up was not clearly reported ^20^

**Synthesis of results**

Due to missing information ^5, 20^, heterogeneity in follow-up time ^15^ and very low baseline weight ^12^, four studies were excluded from the meta-analysis.

*Results from the meta-analysis*

Based on six studies, the overall difference in change scores between groups was 0.19 (95 % CI: 0.13; 0.26) I2= 9.62 %, p= 0.27, indicating comparable reduction in BMI in patients with diabetes compared to patients without diabetes at end of CR. According to the I2 statistics, the result might not be affected by heterogeneity. Stratification on intervention type (Figure 3.1.c) showed statistical no significant group difference (p=0.18). Test of funnel plot asymmetry was not performed due to inclusion of less than <10 studies in the meta-analysis ^10^.

*Results from studies excluded from the meta-analysis*

One study reported a significantly smaller reduction in BMI among patients with diabetes compared to patients without diabetes, however follow-up time was unclear reported ^20^. At 15-months follow-up, one study reported comparable change in BMI ^15^. One study measured both at three- to six months follow-up and at 12-months follow-up and showed comparable changes in BMI.

Figure 3.1.c. Meta-analysis on Body mass index (BMI)

| **Table 3.1.c. Descriptive results on BMI** | | | |
| --- | --- | --- | --- |
| **Author** | **Patients with diabetes** | **Patients without diabetes** | **Between-group comparison** |
| St. Clair et al. ^1^ | Baseline  31±6  Change in BMI, 95% CI (12-week follow-up)  -0.4 (-0.6;-0.2) | Baseline  28±5  Change in BMI, 95% CI (12-week follow-up)  -0.5 (-0.6;-0.4) | Comparable changes in BMI in patients with and without diabetes at end of CR (12 weeks) |
| Gondoni et al.^20^ | Change in BMI (±SD)  -1.2 ± 0.6 | Change in BMI (±SD)  1.4 ± 0.7 | Patients with diabetes reduced BMI significantly less compared to patients with diabetes (unclear number of analysed patients and follow up) |
| Hindman et al. ^11^ | Baseline BMI (±SD)  32.0±6.6  12-week follow-up (±SD)  31.9±6.4 | Baseline BMI (±SD)  29.2±5.5  12-week follow-up (±SD)  28.8±5.2 | No change in BMI among patients with diabetes, BMI decreased significantly patients with diabetes (no comparison between group) |
| Khadanga et al.^16^ | Baseline BMI (±SD)  31.2 ± 5.2  12 to 16-week follow-up (±SD)  30.8 ± 5.1 | Baseline BMI (±SD)  28.1 ± 4.6  12 to 16-week follow-up (±SD)  27.4 ± 4.4 | Patients with diabetes achieved less improvement in BMI compared to patients without diabetes (no insulin resistance) at end of CR (3-4 months). |
| Nishitani et al. ^12^ | Baseline BMI (±SD)  23.3±2.7  6-month follow-up (±SD)  22.6±1.9 | Baseline BMI (±SD)  23.4±2.9  6-month follow-up (±SD)  23.7±2.5 | Comparable changes in BMI in patients with and without diabetes at end of CR (six months) (small sample size, remarkably low BMI) |
| Svacinová et al ^17^ | Baseline BMI (±SD)  29.2±5.1  12-week follow-up (±SD)  29.2±4.8 | Baseline BMI (±SD)  28.3±3.4  12-week follow-up (±SD)  27.8±3.4 | No significant changes in BMI among patients with diabetes, however significant change in BMI among patients without diabetes at end of CR (12 weeks) |
| Toste et al.^18^ | Baseline BMI (±SD)  27.8±3.7  12-week follow-up, change in BMI (±SD)  −0.7±1.0 | Baseline BMI (±SD)  26.3±3.9  12-week follow-up, change in BMI (±SD)  −0.9±1.2 | Decrease in BMI was significantly smaller in patients with diabetes compared to patients without at end of CR (3 months) |
| Carroll et al.^15^ | Baseline BMI (±SD)  28.7±3.3  15-month follow-up (±SD)  28.3±2.9 | Baseline BMI (±SD)  27.0±3.5  15-month follow-up (±SD)  26.8±3.5 | Comparable changes in BMI in patients with and without diabetes at 15 months follow up. |
| Laddu et al.^21^ | Baseline BMI (±SD)  29.1 ± 4.9  12-week follow-up (±SD)  29.1 ± 4.9 | Baseline BMI (±SD)  28.9 ± 4.8  12-week follow-up (±SD)  28.7 ± 4.9 | Comparable changes in BMI in patients with and without diabetes at end of CR (12 weeks). |
| Eser et al. ^5^ | Results only reported in figure | Results only reported in figure | Comparable changes in BMI in patients with and without diabetes at end of CR (3-6 months) and 12 months. |

### 4.1 Lifestyle related

4.1.a Smoking status

**Study characteristics and participants**

The review identified three studies reporting on smoking status ^7, 18, 22^. All studies were observational cohort studies. The reported percentages of patients with diabetes ranged from 12-37 % in the enrolled study population. In one study, only patients with type 2 diabetes were included ^18^. The type of diabetes was not specified in two studies ^7, 22^. In total, smoking status was analysed in n=50345 patients of these, n=7454 had diabetes.

**Interventions**

CR programs were an outpatient service in all three studies ^7, 18, 22^. Patients were in all studies enrolled after discharge from surgical procedure. Interventions were in all studies provided at hospital or a rehabilitation center. CR was provided as an 6-12 week program with three sessions pr. week in two studies ^7, 18^. Number of sessions per week was not provided in Wallert et al. ^22^. Besides exercise sessions, all studies provided educational sessions concerning nutrional counselling and risk factor control ^7, 18, 22^. Smoking cessation courses or consultations were specifically described in two studies ^7, 18^.

**Outcome measurement**

Smoking status was in Suresh et al., reported as percentage of patients stopped smoking at one year (among patients smoking at time of AMI) (including risk difference and 95% CI) ^7^. In Wallert et al., smoking status was reported as smoking absenteeism (including OR and 95 % CI) at one year. In Toste et al., smoking status was reported as smoking (yes/no) in percentages at 12-weeks.

**Synthesis of results**

Due to heterogeneity in follow-up time, meta-analysis was not judged eligible. At 12-weeks follow-up, one study reported comparable reduction of smoking patients ^18^. At one-year follow-up, two studies reported that fewer patients with diabetes had stopped smoking compared to patients without diabetes ^7, 22^.

| **Table 4.1.a. Descriptive results on smoking** | | | |
| --- | --- | --- | --- |
| **Author** | **Patients with diabetes** | **Patients without diabetes** | **Between-group comparison** |
| Suresh et al.^7^ | Stopped smoking at 12-month follow-up: n=51 (54.2%) | Stopped smoking at 12-month follow-up: n= 614 (69.1%) | Fewer patients with diabetes had stopped smoking at one year compared to patients without diabetes (RD: -0.15 (-0.17-0.07) p=0.003) |
| Wallert et al.^22^ | Baseline smokers: n= 1809 (26.8 %) | Baseline smokers: n= 13,069 (32.7%) | Fewer patients with diabetes stopped smoking at 12-week follow-up (OR: 0.90 95% CI (0.81, 0.99) p= 0.035) |
| Toste et al.^18^ | Baseline smokers: n= 55(21.7%)  Reduction in smokers at 12-week follow-up: 90.9% | Baseline smokers: n= n=159 (37.1%)  Reduction in smokers at 12-week follow-up: 83.6% | Comparable reduction in smokers at 12-week follow-up (p=0.266). |

4.1.b. Physical activity

**Study characteristics and participants**

The review identified three studies reporting on physical activity ^2, 18, 23^. All studies were observational cohort studies. The reported percentage of patients with diabetes ranged from 19-40 % in the enrolled study population. Pischke et al. included patients with type 1 or type 2 diabetes (9.8 % reported with type 1 diabetes) ^2^. In two studies, only patients with type 2 diabetes were included ^18, 23^.

**Interventions**

CR programs were an outpatient service in all three studies ^2, 18^. Patients were in all studies enrolled after discharge from surgical procedure. Interventions were in all studies provided at home, hospital or a rehabilitation centre. CR was provided as an 6-12 week program with 2-3 sessions pr. week in two studies ^2, 18, 23^. In Karjalainen, structured homebased exercise sessions were performed two-three times a week ^23^. Besides exercise sessions, two studies provided educational sessions concerning nutrional counselling and risk factor control ^2, 18^, whereas Karjalainen et al. was purely exercise based ^23^.

**Outcome measurement**

Physical activity was in two studies based on self-reported measures; in Pischke et al. patients reported weekly exercise hour ^2^ and in Toste et al., physical activity was measured by International Physical Activity Questionnaire (IPAQ score)^18^. In Karjalainen et al., physical activity was measured by a wristwatch accelerometer ^23^. Toste had follow up at 12 weeks ^18^, Pieschke et al. had follow up at three and 12 months ^2^ while Karjalainen followed up at six months ^23^.

**Synthesis of results**

Due to heterogeneity in follow-up time and measurement method, meta-analysis was not judged eligible. At both 12-weeks ^2, 18^, six-months ^23^ and 12-months follow-up ^2^ comparable changes was found in physical activity.

| **Table 4.1.b. Descriptive results on physical activity** | | | |
| --- | --- | --- | --- |
|  | **Patients with diabetes** | Patients without diabetes | **Between-group comparison** |
| Karjalainen et al. ^23^ | Daily activity at moderate intensity, hours ±SD:  Baseline: 2.40 ± 1.23  Six-month follow-up: 2.36 ±1.06 | Daily activity at moderate intensity, hours ±SD:  Baseline: 3.24 ± 1.17  Six-month follow-up: 3.43 ± 1.21 | Comparable changes in moderate and high intensity physical activity between patients with and without diabetes at six months follow up (p value not significant) |
| Pischke et al.^2^ Men | Exercise hr/wk  Baseline ±SD  1.8±1.7  12-week follow-up ±SD  4.0±3.3  12-month follow-up ±SD  3.8±2.5 | Exercise hr/wk  Baseline ±SD  2.4±2.0  12-week follow-up, ±SD  4.1±2.1  12-month follow-up, ±SD  3.6±2.1 | Comparable changes in physical activity between patients with and without diabetes 12 weeks and 12 months follow up |
| Pischke et al.^2^  *Women* | Exercise hr/wk  Baseline ±SD  1.1 ± 1.1  12-week follow-up ±SD  3.0±1.3  12-month follow-up ±SD  2.8±1.4 | Exercise hr/wk  Baseline, (±SD)  1.6±1.5  12-week follow-up, (±SD)  3.3±1.5  12-month follow-up, (±SD)  3.0±1.7 | Comparable changes in physical activity between patients with and without diabetes 12 weeks and 12 months follow up (female patients with diabetes exercised less) |
| Toste et al.^18^ | IPAQ,score, change from baseline, median: 1283 | IPAQ,score, change from baseline, median: 1380 | Comparable changes in physical activity between patients with and without diabetes at 12 weeks follow up (p=0.628). |

### 5.1 Well-being

5.1. Psychological well-being

No study was identified reporting on psychological well-being.

### 6. Work related

6.1.a Return to work

**Study characteristics and participants**

The review identified one observational study by Suresh et al. reporting on return to work ^7^.

The percentage of patients with diabetes was 12.4 % in the study population. The type of diabetes among diabetes patients was not specified.

The study included in total n=1804 of these, n=223 had diabetes. However, the number of analysed patients on return to work was not specified.

**Interventions**

The CR program was delivered as an outpatient service with patients being enrolled after discharge from surgical procedure. The intervention was provided at hospital as a six week program with three sessions pr. week. Besides exercise sessions, educational components regarding life style modification were provided ^7^.

**Outcome measurement**

Information on return to work was retrieved from a database and performed on patients who were employed at the event of acute myocardial infarction. Estimates were reported as percentages and risk difference with 95 % confidence intervals ^7^.

**Results**

| Table 6.1.a. Descriptive results on return to work | | | |
| --- | --- | --- | --- |
|  | **Patients with diabetes** | **Patients without diabetes** | **Between-group comparison** |
| Suresh et al., ^7^ | Returned to work n=33(47.8%) | n=353(52.7) | Similar return to work percentages in patients with diabetes compared to patients without diabetes at one year. RD: -0.05, 95% CI (-0.03,-0.04), p=0.44 |

### References (Additional file 3)

1. St. Clair M, Mehta H, Sacrinty M, Johnson D, Robinson K. Effects of Cardiac Rehabilitation in Diabetic Patients: Both Cardiac and Noncardiac Factors Determine Improvement in Exercise Capacity : Effects of CR in diabetic patients. Clinical cardiology 2014;**37**(4):233-238.

2. Pischke CR, Weidner G, Elliott-Eller M, Scherwitz L, Merritt-Worden TA, Marlin R, Lipsenthal L, Finkel R, Saunders D, McCormac P, Scheer JM, Collins RE, Guarneri EM, Ornish D. Comparison of Coronary Risk Factors and Quality of Life in Coronary Artery Disease Patients With Versus Without Diabetes Mellitus. The American Journal of Cardiology 2006;**97**(9):1267-1273.

3. Ferrans CE PM. Quality of Life Index. Chicago, IL: College

of Nursing, University of Illinois at Chicago; 1998–1984.

4. Giallauria F, Fattirolli F, Tramarin R, Ambrosetti M, Griffo R, Riccio C, De Feo S, Piepoli MF, Vigorito C. Clinical characteristics and course of patients with diabetes entering cardiac rehabilitation. Diabetes Research and Clinical Practice 2015;**107**(2):267-272.

5. Eser P, Marcin T, Prescott E, Prins LF, Kolkman E, Bruins W, van der Velde AE, Pena-Gil C, Iliou MC, Ardissino D, Zeymer U, Meindersma EP, Van'tHof AWJ, de Kluiver EP, Laimer M, Wilhelm M. Clinical outcomes after cardiac rehabilitation in elderly patients with and without diabetes mellitus: The EU-CaRE multicenter cohort study. Cardiovascular Diabetology 2020;**19**(1).

6. Reibis R, Treszl A, Bestehorn K, Karoff M, Schwaab B, Wirth A, von Horlacher J, Jannowitz C, Pittrow D, Wegscheider K, Völler H. Comparable short-term prognosis in diabetic and non-diabetic patients with acute coronary syndrome after cardiac rehabilitation. Eur J Prev Cardiol 2012;**19**(1):15-22.

7. Suresh V, Harrison RA, Houghton P, Naqvi N. Standard cardiac rehabilitation is less effective for diabetics. International journal of clinical practice 2001;**55**(7):445-448.

8. Yu CM, Lau CP, Cheung BMY, Fong YM, Ho YY, Lam KB, Li LSW. Clinical predictors of morbidity and mortality in patients with myocardial infarction or revascularization who underwent cardiac rehabilitation, and importance of diabetes mellitus and exercise capacity. American Journal of Cardiology 2000;**85**(3):344-349.

9. Yu CM, Li LSW, Lam MF, Siu DCW, Miu RKM, Lau CP. Effect of a cardiac rehabilitation program on left ventricular diastolic function and its relationship to exercise capacity in patients with coronary heart disease: Experience from a randomixed, controlled study. American Heart Journal 2004;**147**(5):e24-e24.

10. Page MJ HJ, Sterne JAC. Chapter 13: Assessing risk of bias due to missing results in a synthesis. In: Higgins JPT TJ, Chandler J, Cumpston M, Li T, Page MJ, Welch VA (editors). (ed). *Cochrane Handbook for Systematic Reviews of Interventions version 6.2*: Cochrane, 2021.

11. Hindman L, Falko JM, LaLonde M, Snow R, Caulin-Glaser T. Clinical profile and outcomes of diabetic and nondiabetic patients in cardiac rehabilitation. American Heart Journal 2005;**150**(5):1046-1051.

12. Nishitani M, Shimada K, Masaki M, Sunayama S, Kume A, Fukao K, Sai E, Onishi T, Shioya M, Sato H, Yamamoto T, Amano A, Daida H. Effect of cardiac rehabilitation on muscle mass, muscle strength, and exercise tolerance in diabetic patients after coronary artery bypass grafting. Journal of Cardiology 2013;**61**(3):216-221.

13. Karjalainen JJ, Kiviniemi AM, Hautala AJ, Piira OP, Lepojärvi ES, Perkiömäki JS, Junttila MJ, Huikuri HV, Tulppo MP. Effects of physical activity and exercise training on cardiovascular risk in coronary artery disease patients with and without type 2 diabetes. Diabetes Care 2015;**38**(4):706-15.

14. Banzer JA, Maguire TE, Kennedy CM, O'Malley CJ, Balady GJ. Results of cardiac rehabilitation in patients with diabetes mellitus. The American Journal of Cardiology 2004;**93**(1):81-84.

15. Carroll S, Tsakirides C, Hobkirk J, Moxon JWA, Moxon JWD, Dudfield M, Ingle L. Differential Improvements in Lipid Profiles and Framingham Recurrent Risk Score in Patients With and Without Diabetes Mellitus Undergoing Long-Term Cardiac Rehabilitation. Archives of Physical Medicine and Rehabilitation 2011;**92**(9):1382-1387.

16. Khadanga DS, Savage AP, Ades AP. Insulin Resistance and Diabetes Mellitus in Contemporary Cardiac Rehabilitation. Journal of Cardiopulmonary Rehabilitation and Prevention 2016;**36**(5):331-338.

17. Svacinova, aacute, Hana, Nov, aacute, kov, aacute, Marie, Placheta Z, Kohzuki M, Nagasaka M, Minami N, Dob, scaron, aacute, k P, Siegelov, aacute, Jarmila. Benefit of Combined Cardiac Rehabilitation on Exercise Capacity and Cardiovascular Parameters in Patients with Type 2 Diabetes. The Tohoku Journal of Experimental Medicine 2008;**215**(1):103-111.

18. Toste S, Viamonte S, Barreira A, Fernandes P, Gomes JL, Torres S. Cardiac rehabilitation in patients with type 2 diabetes mellitus and coronary disease: A comparative study. Revista Portuguesa De Cardiologia 2014;**33**(10):599-608.

19. Laddu D, Ozemek C, Lamb B, Hauer T, Aggarwal S, Stone JA, Arena R, Martin B-J. Factors Associated With Cardiorespiratory Fitness at Completion of Cardiac Rehabilitation: Identification of Specific Patient Features Requiring Attention. Canadian Journal of Cardiology 2018;**34**(7):925-932.

20. Gondoni LA, Titon AM, Nibbio F, Caetani G, Augello G, Mian O, Tuzzi C, Averna E, Parisio C, Liuzzi A. Short-term effects of a hypocaloric diet and a physical activity programme on weight loss and exercise capacity in obese subjects with chronic ischaemic heart disease: a study in everyday practice. Acta Cardiol 2008;**63**(2):153-9.

21. Laddu DR, Ozemek C, Hauer TL, Rouleau CR, Campbell TS, Wilton SB, Aggarwal S, Austford L, Arena R. Cardiometabolic responses to cardiac rehabilitation in people with and without diabetes. International Journal of Cardiology 2020;**301**:156-162.

22. Wallert J, Mitchell A, Held C, Hagstrom E, Leosdottir M, Olsson EG. Cardiac rehabilitation goal attainment after myocardial infarction with versus without diabetes: A nationwide registry study. International journal of cardiology 2019;**292**:19-24.

23. Karjalainen JJ, Kiviniemi AM, Hautala AJ, Niva J, Lepojarvi S, Makikallio TH, Piira O-P, Huikuri HV, Tulppo MP. Effects of exercise prescription on daily physical activity and maximal exercise capacity in coronary artery disease patients with and without type 2 diabetes. Clinical Physiology and Functional Imaging 2012;**32**(6):445-454.

### Additional file 4) Exposure measurement methods, classification of diabetes status

| Reference | Method of classification as reported in study | Measured exposure |
| --- | --- | --- |
| Banzer et al, 2003, USA^27^ | Self-reported history of diabetes or taking medication for glycemic control | Diabetes, type not specified (yes/no) |
| Vergès et al 2003, France ^33^ | At least two fasting plasma glucose levels >7 mmol/l. | Diabetes, type 2 only (yes/no) |
| Hindman et al., 2005, USA ^32^ | Self-reported history of diabetes or taking diabetic medication | Diabetes, type not specified (yes/no) |
| Pischke et al 2006, USA ^31^ | Limited information, diagnosed according to guidelines of the American Diabetes Association) | Diabetes, type 1 or type (yes/no) |
| Svacinová et al 2008 Czech Republic ^30^ | No information | Diabetes, type 2 diabetes only (yes/no) |
| Mourot et al 2010, France ^34^ | Fasting blood glucose concentration greater than 126 mg/dL on at least two occasions and/or antidiabetic treatment. | Diabetes, type 2 only (yes/no) |
| Karjalainen et al 2012, Finland ^35^ | Defined according to Worlds Health Organization criteria, 1999: increased fasting plasma glucose level (two-hour glucose tolerance test), or taking hypoglycemic medication. | Diabetes, type 2 only (yes/no) |
| Wu et al 2012, Taiwan ^28^ | (from Wu, 2008 paper (ref 8): Plasma glucose 6 200 mg/dl (11.1 mM), fasting plasma glucose 6 126 mg/dl (7.0 mM), or under oral hypoglycemic agents and/or insulin treatment. | Diabetes, type 1 or type 2 (yes/no) |
| St. Clair et al, 2013, USA ^29^ | Diagnosed using standard criteria by American Diabetes Association, (ref. 16)). Clinical data from patient records. | Diabetes, type not specified (yes/no) |
| Nishitani et al 2013 Japan ^36^ | Medical treatment, fasting plasma glucose ≥126 mg/dl or casual plasma glucose ≥, or HbA1 ≥ 6.1. Criteria according to Japan Diabetes Society | Diabetes, type not specified (yes/no) |
| Toste et al 2013, Portugal ^37^ | A history of diabetes from medical records or under antidiabetic therapy | Diabetes, type 2 only (yes/no) |
| Armstrong et, al, 2014, Canada ^39^ | Patient reported and hospital chart. Diagnosis confirmed based on hospital coded administrative databases. | Diabetes, type 1 or type 2 diabetes (yes/no) |
| Kenttä et al 2014, Finland ^38^ | No information | Diabetes, type 2 only (yes/no) |
| Kim et al, 2015, Korea^41^ | Fastening plasma glucose test or hemoglobin A1c (HbA1c). | Diabetes, type 2 only (yes/no) |
| Szalewska et al 2015 Poland ^42^ | No information | Diabetes, type 2 only (yes/no) |
| Boukhris et al., 2015, Italy ^40^ | All non-diabetic patients were screened for diabetes before enrollment using fasting serum glucose and glycosylated hemoglobin. | Diabetes, type 2 only (yes/no) |
| Khadanga et al, 2017 USA ^43^ | Fasting serum glucose of ≥126 mg/dL or HbA1c ≥ 6.5 (based on American Diabetes Association). | Diabetes, type 2 only (No insulin resistance, insulin resistance, Type 2 DM) |
| Kasperowicz ^44^ | No information | Diabetes, type 2 only (yes/no) |
| Laddu et.al. 2020, Canada ^45^ | History of type 2 diabetes diagnosed or treated by a physician, or diabetes indicated in hospital record. Diagnosis cross-checked in database and registers to verify. | Diabetes, type 2 only (yes/no) |
| Eser ^46^ | Previous diagnosis with diabetes, insulin or oral antidiabetics, or HbA1c at baseline of ≥ 48 mmol/mol. | Diabetes, type 1 or type 2 (yes/no) |

### Additional file 5) Outcome measurement methods, exercise capacity

|  |  | | | | | |
| --- | --- | --- | --- | --- | --- | --- |
| Outcome measurement method listed in the study | | | | | |  |
| Reference | Procedure | Mode of test; Loading of resistance | Estimated or directly cardiopulmonary exercise testing maximal | Measured outcome | Remarks on reported and analysed results |  |
| Banzer et al., 2003, USA^27^ | Exercise testing in a standard manner using symptom-limited individualized ramp treadmill protocols. Peak MET level was estimated from peak exercise workrate. | Treadmill; ramp | Estimated | METs |  |  |
| Vergès et al. 2003, France ^33^ | Initial workload: 10 W, Increments of 10 W at each 1-min. Cardiopulmonary data, including peak VO2 and VO2 at anaerobic threshold. The obtained data were duration of the test (min), peak workload (W), maximal heart rate, peak VO2 (ml/kg per min) and anaerobic threshold (ml/kg per min). | Bicycle ergometer; incremental | Directly | VO_2_peak | VO_2_ peak was converted into METs assuming that 1 MET equals 3.5 ml/min/kg resting oxygen. |  |
| Hindman et al., 2005, USA ^32^ | Graded symptom-limited maximal stress test according to a standard Bruce protocol at entry and upon program completion to calculate exercise capacity in metabolic equivalent time. | Treadmill; unknown | Estimated | METs |  |  |
| Pischke et al. 2006, USA ^31^ | Symptom-limited maximal graded exercise testing using the Bruce protocol. METs were automatically calculated by the testing device during exercise testing | Treadmill; unknown | Estimated | METs | Provided estimates are stratified on gender in paper. For the purpose of meta-analysis, gender stratified mean difference on exercise capacity have been treated as two separate study populations, referred to as Pischke, Male and Pischke, Female |  |
| Svacinová et al. 2008 Czech Republic ^30^ | Progressively increasing working rate to maximal tolerance. Test starting at workload 30 watts with progressively increasing working rate (20 W/2 min). Oxygen uptake and carbon dioxcide production were measured by analysis of blood gas samples taken breath by breath. | Bicycle ergometer; unknown | Directly | VO_2_peak | VO_2_ peak was converted into METs assuming that 1 MET equals 3.5 ml/min/kg resting oxygen. |  |
| Mourot et al. 2010, France ^34^ | The exercise stress test with a gas exchange measurement. The exercise stress test provided measurement of peak oxygen uptake (VO2max) and ventilatory threshold. | Treadmill; ramp | Directly | VO_2_ max | Results on METs were originally provided stratified on interventional procedure (CAGB/PTCA). Unified data were kindly provided upon request by corresponding author.  VO2 peak was converted into METs assuming that 1 MET equals 3.5 ml/min/kg resting oxygen.  Relatively low VO2 max values might be due to the use of a ramp protocol resulting in a more exhaustive test procedure. |  |
| Karjalainen et al. 2012, Finland ^35^ | Symptom-limited maximal exercise test. Test start: 30 W, increments at 15 W (men) and 10 W (women) each 1 minute, until voluntary exhaustion or ST depression > 0·2 mV in ECG. Gas exchange monitored continuously. The highest 1-min mean value of oxygen consumption = VO2peak. Maximal workload (W) and maximal metabolic equivalents (METs) were calculated as the average workload and METs during the last minute of the test. | Bicycle ergometer; incremental | Directly | VO_2_peak (ml/kg-1xmin-1) + METsmax | VO_2_ peak was converted into METs assuming that 1 MET equals 3.5 ml/min/kg resting oxygen. Lower exercise capacity values might be due to the use of bicycle ergometer. |  |
| Wu et al. 2012, Taiwan ^28^ | Test starting af 25 W, increments every 3 minutes, Pedaling rate of 50 to 60 rpm. Until volitional fatigue, heart rate at 90 % of the maximum, a respiratory exchange ratio >1.15, or signs of exercise intolerance. VO_2_ peak was measured by a metabolic Measurement System using breath by breath technique. | Bicycle ergometer; incremental | Directly | VO_2_ peak | The study was excluded for the meta-analysis due to lack of estimates provided in the paper. Corresponding author was contacted by email unsuccessfully. |  |
| St. Clair et al., 2013, USA ^29^ | Steady-state MET levels were recorded at each session and were obtained either automatically from exercise devices or using a standardized MET formula. | Unknown; Unknown | Estimated | METs | The study have been excluded for the meta-analysis due to sparse reporting of test procedure. Reported METs are notably lower than expected. |  |
| Nishitani et al. 2013 Japan ^36^ | Testing using an expiratory gas analysis machine.  Starting at 20 W, followed by loading (15 W/min) until exhaustion, progressive angina, ST-segment depression (≥2 mm), or sustained tachyarrhythmia. | Bicycle ergometer; ramp | Directly | Peak VO_2_ | Lower exercise capacity values might be explained by a high prevalence of comorbidity in the study population:  Ejection fraction: DM=59.7±16 No DM= 65.3±12. Off-pump CABG surgery: DM=97 % No DM= 100 %. |  |
| Toste et al. 2013, Portugal ^37^ | Maximal functional capacity was estimated, based on the metabolic equations of the American College of Sports Medicine for treadmill exercise. | Treadmill; unknown | Estimated | METs |  |  |
| Armstrong et, al, 2014, Canada ^39^ | Bruce or modified Bruce protocol. Peak estimated MET value calculated from speed and grade at final stage of the exercise protocol using an established equation. | Treadmill; unknown | Estimated | METs | Provided estimates are stratified on gender in paper. For the purpose of meta-analysis, gender stratified mean difference on exercise capacity have been treated as two separate study populations, referred to as Armstrong, Male and Armstrong, Female |  |
| Kenttä et al. 2014, Finland ^38^ | Symptom-limited test. Initial workload of 30 W with a gradual increase of load in steps of 10 (women) and 15 W/min (men). Testing was continued until voluntary exhaustion or ST-segment depression exceeding 0.2 mV. | Bicycle ergometer; unknown | Directly | METs | Low exercise capacity values might be due to the use of bicycle ergometer. |  |
| Kim et al., 2015, Korea^41^ | Modified symptom limited Bruce protocol. Respiratory gas analyzer, METs during the maximal exercise period and peak oxygen consumption (VO2peak). | Treadmill; unknown | Directly | VO_2_peak, (mL/kg/min) + METs | For the purpose of meta-analysis, provided METs in paper have been used. |  |
| Szalewska et al. 2015 Poland ^42^ | Symptom-limited exercise test according to the Bruce protocol. Monitored during test: maximal workload (METs), heart rate, blood pressure (BP, mm Hg) at rest and at maximal effort. | Treadmill; unknown | Estimated | METs |  |  |
| Boukhris et al., 2015, Italy ^40^ | Maximal exercise testing, using ramp protocol with maximal metabolic equivalents (METs) measurement. | Treadmill; ramp | Estimated | METs |  |  |
| Khadanga et al., 2017 USA ^43^ | Symptom limited exercise tolerance test. Peak metabolic equivalents were estimated based on treadmill speed and elevation. For 517 patients, expired gas was analyzed during the exercise protocol. Peak VO2 was considered to be the highest 30-second average during the test. | Treadmill; unknown | Estimated /Directly | Peak V̇O_2_ (mL O_2_kg/min) + METs | For the purpose of meta-analysis, VO_2_ peak was converted into METs assuming that 1 METs equals 3.5 ml/min/kg resting oxygen. |  |
| Kasperowicz et.al., 2019 ^44^ | Means of exercise ECG test according to Bruce protocol | Treadmill; unknown | Estimated | METs |  |  |
| Laddu et.al. 2020, Canada ^45^ | Symptom-limited maximal test to determine peak metabolic equivalents (METs). The peak MET value was calculated from speed and grade during the final stage of the exercise protocol using an established equation. | Treadmill; unknown | Estimated | METs |  |  |
| Eser et al., 2020 | Cardiopulmonary exercise test with ergometry, electrocardiogram registration. Initial workload: 5 WATT for 3 min. Individual RAMP protocol was applied for optimal duration of 8–12 min. | Bicycle, Treadmill*; ramp | Directly* | VO_2_ peak | *Data collected from baseline study ^62^: Treadmill was used in seven patients. Due to mask intolerance, signs of mask leakage or short test duration, VO_2_peak was calculated using the maximum watts in 102 patients ^62^. A smaller fraction of the study population (n >48 patients: 3,6 % was tested by 6 min walking test)^62^  VO_2_ peak was converted into METs assuming that 1 MET equals 3.5 ml/min/kg resting oxygen. |  |

### Additional file 6) Subgroup analysis

#### Figure AD1 Forest plot of subgroup analysis on type of diabetes


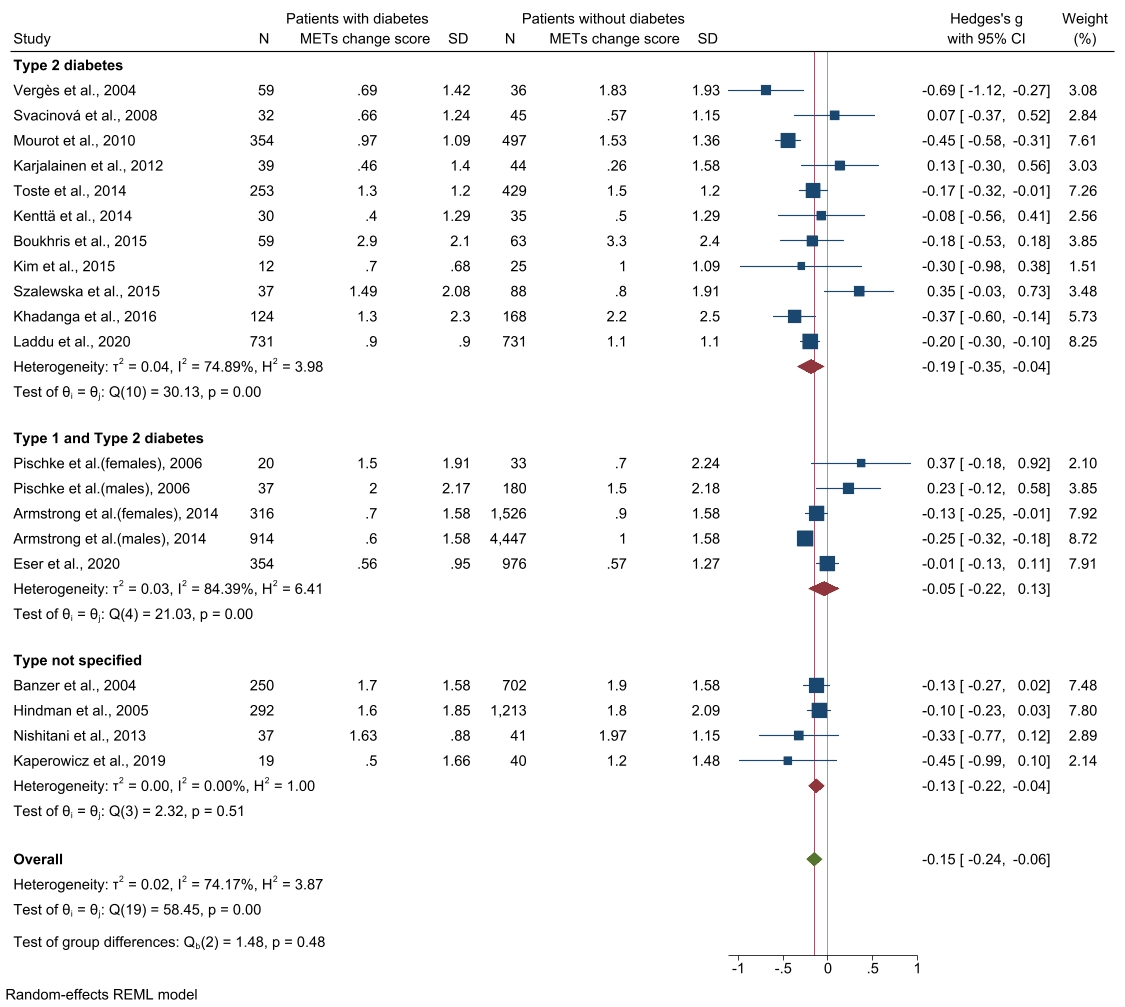


#### Figure AD2 Subgroup analysis on type of intervention


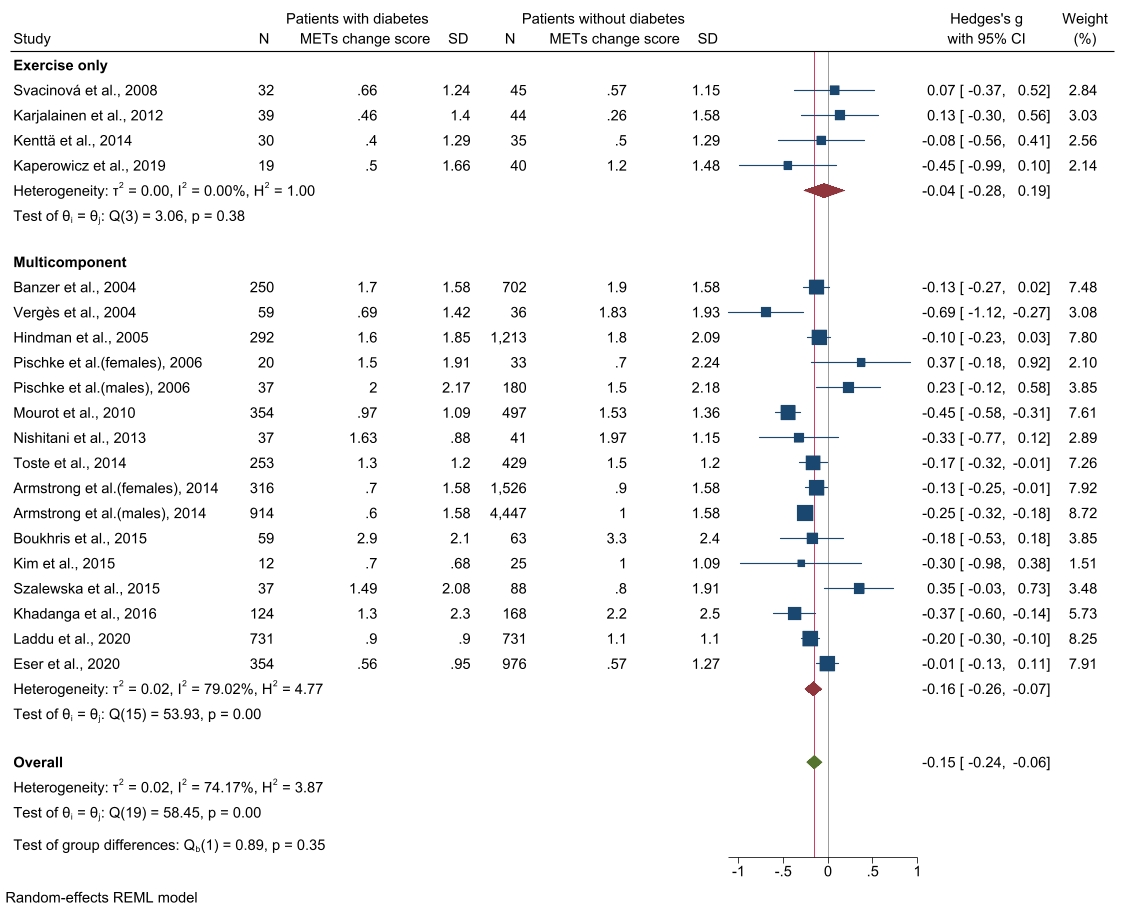


#### Figure AD3 Subgroup analysis on length of trial follow up

#### ****

#### Figure SM6.4 Funnel plot for assessing publication bias from start of CR to end of intervention

#### Figure AD6.5 Funnel plot for assessing publication.bias

1. Ferrans and Powers Quality of Life Index Cardiac version IV questionnaire at baseline and at 12 weeks [↑](#footnote-ref-1)
2. Medical Outcomes Study shortform health survey (MOS SF-36) at baseline, three months and 12 months [↑](#footnote-ref-2)
3. Glucose, mg/dL [↑](#footnote-ref-3)
4. HbA1c [↑](#footnote-ref-4)
5. HbA1c, % (sd) [↑](#footnote-ref-5)
6. HbA1c mmol/mol [↑](#footnote-ref-6)
