# Supplementary material for: Benefits of cardiac rehabilitation following acute coronary syndrome for patients with and without diabetes: a systematic review and meta-analysis
Source: BMC Cardiovasc Disord. 2022 Jun 27;22:295. doi: 10.1186/s12872-022-02723-5 (PMC9237976; doi:10.1186/s12872-022-02723-5)
Supplement: Supplementary file 2 — Additional file 2. BACPR Standards and Core Components. [file 12872_2022_2723_MOESM2_ESM.docx]

### Additional file 2) BACPR Standards and Core Components

| **BACPR Standards and Core Components ^15^** | **Health behaviour change and education** | **Lifestyle risk factor** | **Psychosocial health** | **Medical risk management** | **Long-term strategies** | **Audit and evaluation** | **Score** |
| --- | --- | --- | --- | --- | --- | --- | --- |
| **Banzer et al, 2003, USA** | 1 | 1 | 1 | 1 | 0 | 1 | 5 |
| **Vergès et al 2003, France** | 1 | 1 | 0 | 1 | 0 | 1 | 4 |
| **Hindman et al., 2005, USA** | 1 | 1 | 1 | 1 | 0 | 1 | 5 |
| **Pischke et al 2006, USA** | 1 | 1 | 1 | 0 | 1 | 1 | 5 |
| **Svacinová et al 2008 Czech Republic** | 0 | 1 | 0 | 0 | 0 | 1 | 2 |
| **Mourot et al 2010, France** | 1 | 1 | 0 | 1 | 0 | 1 | 4 |
| **Karjalainen et al 2012, Finland** | 0 | 1 | 0 | 0 | 0 | 1 | 2 |
| **Nishitani et al 2013 Japan** | 1 | 1 | 0 | 0 | 0 | 1 | 3 |
| **Toste et al 2013, Portugal** | 1 | 1 | 1 | 0 | 0 | 1 | 4 |
| **Armstrong et, al, 2014, Canada** | 1 | 1 | 1 | 1 | 0 | 1 | 5 |
| **Kenttä et al 2014, Finland** | 0 | 1 | 0 | 0 | 0 | 1 | 2 |
| **Kim et al, 2015, Korea** | 1 | 1 | 0 | 1 | 1 | 1 | 5 |
| **Szalewska et al 2015 Poland** | 1 | 1 | 0 | 0 | 0 | 1 | 3 |
| **Boukhris et al., 2015, Italy** | 0 | 1 | 1 | 0 | 0 | 1 | 3 |
| **Khadanga et al, 2017 USA** | 1 | 1 | 1 | 0 | 1 | 1 | 5 |
| **Kasperowicz et al., 2019 , Poland** | 0 | 1 | 0 | 0 | 0 | 1 | 2 |
| **Laddu et al. 2020, Canada** | 1 | 1 | 0 | 1 | 0 | 1 | 4 |
| **Eser et al., 2020, Switzerland** | 1 | 1 | 0 | 0 | 0 | 1 | 3 |
| **total** | 13 | 18 | 7 | 7 | 3 | 18 |  |

### Additional file 3) Secondary outcomes

1.1 Health Related Quality of Life (HRQoL)

**Study characteristics and participants**

The review identified two studies reporting on HRQoL^1, 2^. Both studies were observational cohort studies.

The percentage of patients with diabetes ranged from 20.7%- 28.2 % in the study populations. St. Clair did not specify the type of diabetes ^1^, while Pischke et al. included patients with type 1 or type 2 diabetes (9.8 % reported with type 1 diabetes) ^2^.

In total HRQoL was analysed in total n=970 patients of these, n=461 had diabetes.

**Interventions**

CR programs were delivered as an outpatient service with patients being enrolled after discharge from surgical procedure. Both interventions were provided at hospital or medical center as a 12-week program with three sessions pr. week. Besides exercise sessions, health and nutrition education sessions were provided. In Pischke et al., stress management was facilitated and patients continued to meet in groups weekly for the next 40 weeks after the intervention for long term adherence ^2^.

**Outcome**

HRQoL were measured with two different self-reported questionnaires. St. Clair et.al. used the disease specific Ferrans and Powers Quality of Life Index Cardiac version IV questionnaire at baseline and at 12 weeks ^3^. Pischke et al. used the generic Medical Outcomes Study shortform health survey (MOS SF-36) at baseline, three months and 12 months and computed two aggregated scores on physical and mental components.

**Synthesis of results**

Due to heterogeneity in outcome measures (generic and disease specific questionnaires) pooling of the results was not judged eligible. Between group comparison showed comparable changes in HRQoL in both studies at 12-weeks follow-up ^1, 2^. In Pischke et al. ^2^ comparable changes in HRQoL remained at one-year follow-up.

| Table 1.1 Descriptive results on Health Related Quality of Life (HRQoL) | | | | | | | | |
| --- | --- | --- | --- | --- | --- | --- | --- | --- |
| **Study** | **Patients with diabetes** | | | | **Patients without diabetes** | | | **Between group comparison** |
| St. Clair et al. ^1^ | Mean change, 95 % CI: 1.9 (1.4-2.4)^[[1]](#footnote-1)^ | | | | Mean change, 95 % CI: 1.8 (1.5-2.1)^1^ | | | Comparable changes in HRQoL between groups at 12 weeks follow up (p value not significant) |
| Pischke et al. ^2^ | Physical health^[[2]](#footnote-2)^ | Baseline (SD) | 3 months (SD) | 12 months (SD) | Baseline (SD) | 3 months (SD) | 12 months (SD) | Comparable changes in HRQoL between groups at 12 weeks and one year follow up |
|  | Men | 46.5±12.1 | 50.3± 9.2 | 51.8±10.1 | 48.4±10.3 | 52.9±8.8 | 52.1± 9.7 |  |
|  | Women | 40.0±10.1 | 47.4±10.5 | 52.0±9.2 | 47.5±9.7 | 54.4±8.9 | 52.0±9.2 |  |
| Pischke et al. ^2^ | Mental health^2^ |  |  |  |  |  |  | Comparable changes in HRQoL between groups at 12 weeks and one year follow up |
|  | Men | 46.5±12.1 | 50.3±9.2b | 51.8±10.1 | 48.4±10.3 | 52.9±8.8 | 52.1±9.7 |  |
|  | Women | 40.0±10.1 | 47.4±10.5 | 46.2±12.5 | 47.5±9.7 | 54.4±8.9 | 52.0±9.2 |  |

### 2.1 Cardiovascular related

2.1.a. Mortality

The review identified five studies reporting on mortality. All studies were all observational cohort studies. The reported percentage of patients with diabetes ranged from 12.4-32.0% in the enrolled study populations. Two studies included patients with type 1 or type 2 diabetes ^4, 5^. The type of diabetes was not specified in three studies ^6-8^. In total, mortality was analysed in n= 5932 patients of these, n= 1100 had diabetes.

**Interventions**

CR programs were an outpatient service in two studies ^4, 7^. Reibis et al. provided CR as an inpatient program. In Yu et al. the intervention was delivered in three phases: inpatient, outpatient and homebased intervention with follow up visits to monitor progress ^8^. Eser et al. was a multicentre study provided CR as both in- and outpatient programme ^5^. Patients were in all studies enrolled after discharge from surgical procedure. Interventions were in all studies provided at hospital or a rehabilitation centre. CR was provided as a three-week program in Reibis et al. ^6^, a six-week program in Suresh et.al. ^7^, and a 12-months program in Yu et a l^8^ and Eser et al., provided CR as three weeks to three months programme ^5^. In Giallauria et al., the duration of the program was not reported ^4^.Two studies provided two sessions per week ^7, 9^. Eser et al., provided in 10-36 sessions in total ^5^ Two studies did not report the number of sessions ^4, 6^. Besides exercise sessions, all four studies provided educational sessions concerning nutrional counselling and risk factor control. Additionally, Yu et al. offered vocational guidance ^8^.

**Outcome**

Mortality was reported as over-all mortality and/or cardiac mortality. Follow up was performed at 14 days in Giallauria et al. ^4^, 12 months in Suresh et al. and Eser et al. ^5, 7^, 13.4 months in Reibis et al. ^6^, and 3.2 years in Yu et al. ^8^.

**Synthesis of results**

Due to heterogeneity in follow-up time and reporting on over-all mortality pooling of the results was only judged eligible in three studies measuring cardiac mortality at one year ^5-7^.

*Results from the meta-analysis (cardiac mortality)*

The meta-analysis based on the three studies ^5-7^ showed a statistically significant increased risk of cardiac mortality at one year in patients with diabetes (OR, 2.16 [95% CI: 1.49-3.13]; I^2^= 48.9 %, p=0.00). The may represent moderate heterogeneity. Test of funnel plot asymmetry was not performed due to inclusion of less than <10 studies in the meta-analysis ^10^.

*Results from studies not included in the meta-analysis (over-all mortality)*

At 14-days follow-up, one study found comparable mortality between groups ^4^. At one-year follow up, two studies found comparable mortality ^5, 6^ whereas one study found higher mortality at one-year among patients with diabetes ^7^. At three-years follow-up, one study found mortality higher in patients with diabetes ^8^.

| **Table 2.1.a. Descriptive results on cardiac- and over-all mortality** | | | |
| --- | --- | --- | --- |
| **Study** | Patients with diabetes | **Patients without diabetes** | **Between group comparison** |
| Giallauria et al. ^4^ | Overall mortality: 1% | Overall mortality: 0.5% | Over-all mortality was comparable between groups at 14 days follow up (p=0.23) |
| Reibis et al.^6^ | Cardiac mortality: 1.2 % | Cardiac mortality: 1.5 % | Over-all and cardiac mortality were comparable between groups at one year.  Overall mortality:  Adjusted odds ratio (95 % CI):  0.37 (0.07-2.02) (p=0.252)  Mortality due to cardiovascular events (p=0.874) |
| Suresh et al. ^7^ | Over-all mortality:15.7 %  Cardiac mortality: 13.4 % | Over-all mortality:5.6 %  Cardiac mortality: 5.4 % | Over-all and cardiac mortality was statistically significant higher in patients with diabetes at one year follow up.    Risk difference (95 % CI):  Over-all mortality: 0.10 (0.05-0.15)  Cardiac mortality: 0.08 (1.69-3.70) |
| Yu et al. ^8^ | Over-all mortality: 23.4 % | Over-all mortality: 5.5 % | Over-all mortality was statistical significantly higher in patients with diabetes at three year follow up  Relative Risk:  4.3, p=0.03 |
| Eser et al. ^5^ | Over-all mortality:2.3 %  Cardiac mortality: 2.3 % | Over-all mortality: 1.2 %  Cardiac mortality: 0.6 % | Overall mortality was comparable between groups at one year follow up (p=0.209) |
|  |  |  | Cardiac mortality was statistically significant higher in patients with diabetes at one year follow up (p=0.009) |

Figure 2.1.a. Meta-analysis on cardiac mortality

2.1.b. Myocardial infarction (MI)

The review identified three observational studies reporting on myocardical infarction (MI) ^5-7^. The reported percentage of patients with diabetes ranged from 12.4-26.4 % in the enrolled study populations. The type of diabetes was not specified in two of the studies ^6, 7^. Eser et al. included type 1 and type 2 diabetes. In total, MI was analysed in n= 4697 patients of these, n= 884 had diabetes.

**Interventions**

Reibis et al. provided CR as an inpatient program, whereas Suresh et al. was an outpatient program^6, 7^. Eser et al. was a multicentre study provided CR as both in- and outpatient programme ^5^. Patients were enrolled after discharge from surgical procedure. Interventions were provided at hospital or a rehabilitation centre. CR was provided as a three-week program in Reibis et al. ^6^, a six week program in Suresh et.al. ^7^ and Eser provided CR as three weeks to three months programme ^5^. Suresh et al. provided two sessions per week while Reibis et al. did not report the number of sessions per week ^6, 7^. Eser et al., provided in 10-36 sessions in total ^5^. Besides exercise sessions, all studies provided educational sessions concerning nutrional counselling and risk factor control.

**Outcome**

Follow up was performed at 12 months in Suresh et al. and Eser et.al. ^5, 7^ and 13.4 months in Reibis et al. ^6^.

**Synthesis of results**

*Results from the meta-analysis*

| **Table 2.1.b. Descriptive results on myocardial infarction (MI)** | | | |
| --- | --- | --- | --- |
| **Study** | **Patients with diabetes** | **Patients without diabetes** | **Comparison of patients with diabetes versus patients without diabetes** |
| Reibis et al.^6^ | 2.9 % | 2.4 % | Comparable risk of myocardial infarction between groups at one year follow up (p=0.646) |
| Suresh et al. ^7^ | 4.5% | 6.5 % | Comparable risk of reinfarction between groups at one year follow up  (risk difference: -0.02 95 % CI [-0.05-0.01]; p=0.24) |
| Eser et al. ^5^ | 2.3 % | 1.8 % | Comparable risk of acute myocardial infarction between groups at one year follow up (p=0.325) |

Pooling of results from the three studies showed comparable risk of myocardial infarction between groups at one-year follow-up (OR, 0.94 95% CI [0.62, 1.45]; I^2^= 2.8 % p= 0.792). Results may not be affected by heterogeneity. Test of funnel plot asymmetry was not performed due to inclusion of less than <10 studies in the meta-analysis ^10^.

Figure 2.1.b. Meta-analysis on myocardial infarction (MI)

2.1.c. Revascularisations

Same study characteristics and interventions as 2.1.c.

**Outcome**

Revascularisation was reported as coronary bypass artery grafting (CABG) or percutaneous coronary intervention (PCI/PTCA) and cardiac intervention (PCI or CABG).

Follow up was performed at 12 months in Suresh et al. and Eser et.al. ^5, 7^ and 13.4 months in Reibis et al. ^6^.

| **Table 2.1.c. Descriptive results on revascularisations** | | | |
| --- | --- | --- | --- |
| Study | Patients with diabetes | Patients without diabetes | Comparison of patients with diabetes versus patients without diabetes |
| Reibis et al.^6^ | PCI: 14.0 %,  CABG: 1.7% | PCI: 13.1 %,  CABG: 1.8 % | Comparable risk of PCI/CABG between groups at one year follow up  (PCI p=0.67)  (CABG p=0.646) |
| Suresh et al. ^7^ | PTCA: 0.9 %,  CABG: 12.6 % | PTCA: 2.5 %,  CABG: 12.7 % | Comparable risk of PCI/CABG between groups at one year follow up  (risk difference: -0.01 95 % CI [-0.06-0.03]; p=0.6) |
| Eser et al. ^5^ | PCI or CABG: 9.9 % | PCI or CABG: 8.1 % | Comparable risk of cardiac intervention between groups at one year follow up  (p=0.325) |

*Results from the meta-analysis*

Pooling of results from the three studies showed comparable risk of revascularisation between groups at one-year follow-up (OR, 1.07 95 % CI [0.86,1.45]; I^2^=18.6 % p=0.540). Results may not be affected by heterogeneity. Test of funnel plot asymmetry was not performed due to inclusion of less than <10 studies in the meta-analysis ^10^.

Figure 2.1.c. Meta-analysis on revascularisations

2.1.d. Hospital readmission

The review identified two studies reporting on hospital readmission ^6, 9^. Eser et al., reported hospitalisation for cardiac reason ^5^. All studies were all observational cohort studies. The reported percentage of patients with diabetes ranged from 16.6-32% in the enrolled study populations. Eser et al., included patients with type 1 and type 2 diabetes ^5^. The type of diabetes was not specified in two of the studies ^6, 9^.

In total, hospital readmission was analysed in n= 3496 patients of these, n= 795 had diabetes.

**Interventions**

Reibis et al. provided CR as an inpatient program, whereas Yu et al. was an combined in- and outpatient program with homebased follow up. Eser et al. was a multicentre study provided CR as both in- or outpatient programme ^5^. Patients were enrolled after discharge from surgical procedure. Interventions were provided at hospital or a rehabilitation centre. CR was provided as a three week program in Reibis et al. ^6^, a six months program with maintenance phone calls in Yu et.al. for up to a year ^9^ and Eser provided CR as three weeks to three months programme ^5^.Yu et al. provided two sessions per week ^8^. Eser et al., provided in 10-36 sessions in total ^5^. Reibis et al. did not report the number of sessions per week ^6^. Besides exercise sessions, all studies provided educational sessions concerning nutrional counselling and risk factor control.

**Outcome**

One study reported all cause readmission ^6^, whereas two studies reported readmission due to cardiac reasons ^5, 8^. Mean follow up was 13.4 months in Reibis et al. ^6^. Mean follow up in Yu et al. was 3.2±1.1 years ^9^. Follow up in Eser et al. was 12 months ^5^.

**Synthesis of results**

Due to heterogeneity in outcome and follow-up time, meta-analysis on readmission were not judged eligible. Comparable risk of readmission at one year were found in two studies ^5, 6^ whereas one study showed higher risk of readmission for patients with diabetes at three years follow-up ^9^

| Table 2.1.d. Descriptive results on hospital readmission | | | |
| --- | --- | --- | --- |
| Study | Patients with diabetes | Patients without diabetes | Between-group comparison |
| Reibis et al.^6^ | 21.1 % | 19.8 % | Comparable risk of all cause readmission between groups at one year follow up (p=0.428) |
| Yu et al. ^9^ | 32% | 23% | Patients with diabetes had a higher risk of cardiac related readmission compared to patients without diabetes at three years follow up  (Relative risk: 4.3 p=0.03) |
| Eser et al. ^5^ | 10.9% | 7.9 % | Comparable risk of cardiac related readmission between groups at one year follow up (p= 0.089) |

### 3.1. Diabetes related

3.1.a Blood glucose level

**Study characteristics and participants**

The review identified four observational ^1, 5, 11, 12^ and one controlled cohorte study reporting on blood glucose level ^13^.

The reported percentage of patients with diabetes ranged from 19.4 %- 28.2 % in the enrolled populations. In two studies, only patients with type 2 diabetes were included ^12, 13^. One study included type 1 and type 2 diabetes ^5^. The type of diabetes was not specified in two studies ^1, 11^. In total, blood glucose was analysed in n=4095 patients of these, n=1079 had diabetes.

**Interventions**

CR programs were delivered as an outpatient service with patients being enrolled after discharge from surgical procedure. Interventions were in three studies provided at hospital or a medical center ^1, 11, 12^. Eser et al. was a multicentre study provided CR as both in- and outpatient programme ^5^. In Karjalainen et al., the exercise sessions were structured home based sessions ^13^. CR was provided as a 12 week program with three sessions pr. week in two studies ^1, 11^. In Nishitani et al. and Karjalainen et al., interventions lasted six and nine months respectively. Eser et al., provided in 10-36 sessions in programmes lasting three weeks- three months ^5^ Besides exercise sessions, four studies provided educational sessions concerning nutrional and risk factor control ^1, 5, 11, 12^.

**Outcome measurement**

Blood glucose was measured by fasting blood glucose in three studies ^11-13^. Eser et al., were non-fasting ^5^. St. Clair et al. did not specify the measurement method ^1^. Three studies measured blood glucose at baseline and at 12 weeks follow up ^1, 11^. Nishitani et al. and Karjalainen et al measured at baseline, and at 6 months and 24 months respectively. Eser et al. measured at end of CR and at 12 months ^5^.

**Synthesis of results**

Due to heterogeneity in measurement and reporting meta-analysis was not performed. We limited the descriptive results to within group comparison in patients with diabetes. Two studies reported significantly decreases in blood glucose levels at 12 weeks/end of CR ^1, 5^, however an increase was found at one-year in Eser et al. ^5^. No significant changes were found in three studies at 12-weeks, six-months and two-year follow-up ^11-13^.

| **Table 3.1.a. Descriptive results on blood glucose level** | | | |
| --- | --- | --- | --- |
| **Study** | **Patients with diabetes** | **Patients without diabetes** | **Within-group comparison (patients with diabetes)** |
| St. Clair et al. ^1^ | Mean change (95 %CI):  1.2 mg/dl (−4.6 to 6.9)^[[3]](#footnote-3)^  Mean change (95% CI):  −0.5 (−0.8 to −0.3)^[[4]](#footnote-4)^ | Mean change (95% CI):  0.1 (−0.1 to 0.2)^3^  Mean change (95% CI):  −0.5 mg/dl (−2.1 to 1.2)^4^ | Patients with diabetes significantly decreased glycated hemoglobin at 12 weeks follow-up. |
| Karjalainen et al. ^13^ | Baseline: 6.6 ± 0.8^[[5]](#footnote-5)^  Follow up: 6.5 ± 1.0^5^ | Baseline: 5.9 ± 0.4^5^  Follow up: 5.7 ± 0.3^5^ | No significant decrease in blood glucose levels in patients with diabetes at two years follow-up. |
| Hindman et al. ^11^ | Baseline: 142.2 ±48.8^3^  Follow-up: 136.9± 46.1^3^  (3.7 % improvement) | Baseline: 101.4± 24.5^3^  Follow-up: 99.7±17.5^3^  (1.7 % improvement) | No significant decrease in blood glucose levels in patients with diabetes at 12 weeks follow-up (p=0.05) |
| Nishitani et al. ^12^ | Baseline:  143±57 ^3^,  7.0±1.3^5^  Follow-up:  167±68^3^,  7.2±1.4^5^ | Baseline:  103±14^3^,  5.1±0.4%^5^  Follow-up:  112±20^3^  5.2±0.5^5^ | No significant decrease in blood glucose levels in patients with diabetes at six-months weeks follow-up (p>0.05) |
| Eser et al. ^5^ | Median change (baseline to end of CR:  − 1.0^[[6]](#footnote-6)^  Mean change (end of CR to one year:  1.1 mmol/mol | Median change baseline-end of CR:  0.1 mmol/mol  Mean change baseline-one year:  Remained stable | Changes in blood glucose levels were significantly better in patients with diabetes at end of CR, however increased at one-year follow-up. |
|  |  |  |  |

### 3.1.b. Weight

**Study characteristics and participants**

The review identified seven studies reporting on weight ^1, 2, 12, 14-17^. All studies were all observational cohort studies.

The reported percentage of patients with diabetes ranged from 11.5-41.6 % in the enrolled study populations. In three studies, only patients with type 2 diabetes were included ^12, 16, 17^. Pischke et al. included patients with type 1 or type 2 diabetes (9.8 % reported with type 1 diabetes). The type of diabetes was not specified in three studies ^1, 14^. In total, weight was analysed in n=2549 patients of these, n=1376 had diabetes.

**Interventions**

CR programs were delivered as an outpatient service with patients being enrolled after discharge from surgical procedure. Interventions were in three studies provided at hospital or a medical center. CR was provided as a 10-12 week program with three sessions pr. week in six studies ^1, 2, 14-17^. In Nishitani et al. and Carroll et al., interventions lasted six and 15 months respectively. Besides exercise sessions, seven studies provided educational sessions concerning nutrional and risk factor control ^1, 2, 12, 14-16^. Additionally, Khadanga et. al. offered a behavioural weight loss programme for patients with BMI >30 ^16^. Svacinova et.al. offered exercise sessions only ^17^.

**Outcome measurement**

In Banzer et al. weight was only reported for patients with a BMI of ≥30. Weight was reported in pounds (lbs) in two studies ^1, 14^ and kilograms (kg) in five studies ^2, 12, 15-17^. All studies measured weight at baseline. Five studies measured weight at 10-12 weeks follow up ^1, 2, 14, 16, 17^. Nishitani et al. and Carroll et al. measured weight at six and 15 months follow up ^12, 15^

***Results***

*Meta-analysis on weight*

Based on four studies, the overall difference in change scores between groups was 0.20 (95 % CI: 0.04; 0.37) I^2^= 48.32 %, p= 0.10, indicating comparable weight loss in patients with diabetes compared to patients without diabetes. According to the I^2^ statistics, the result might be affected by moderate heterogeneity. Stratification on intervention type (Figure 3.1.b) showed statistical significant group difference (p=0.03) with exercise only resulting in bigger weight change in patients without diabetes. Test of funnel plot asymmetry was not performed due to inclusion of less than <10 studies in the meta-analysis ^10^.

*Descriptive results from studies excluded from meta-analysis*

Regarding the three remaining studies neither of the studies reported between group comparison and showed mixed results; one study reported significant weight loss in both groups ^15^, in another study no weight loss was seen in patients with diabetes, oppositely, weight loss was seen in patients without diabetes ^14^. In the third study, average body weight was very low and weight loss was not considered relevant ^12^.

| **Table 3.1.b. Descriptive results on weight** | | | |
| --- | --- | --- | --- |
| Author | **Patients with diabetes** | Patients without diabetes | **Between-group comparison** |
| Banzer et al.^14^ | Baseline, kg:  107.0  12-week follow-up, kg:  107.0 | Baseline, kg:  98.4  12-week follow-up, kg:  96.2 | Weight change was only reported in patients with BMI≥30 (n=184):  No weight change among patients with diabetes, however a significant weight change in patients without was observed after CR. No between group comparison.  Excluded in meta-analysis due to lack of reporting of n in groups. |
| St. Clair et al. ^1^ | Baseline, kg (±SD):  93.0 ±20.9  12-week, follow-up kg:  92.0 | Baseline, kg (±SD):  84.8 ±18.1  12-week follow-up, kg:  83.5 | Patients with diabetes reduced weight to a lesser extent when compared with patients without diabetes however not statistically significant. |
| Carroll et al. ^15^ | Baseline, kg (±SD)  83.4 ±11.3  15-month follow-up, kg (±SD):  81.7(±10.1) | Baseline, kg (±SD)  79.0 ±12.0  15-month follow-up, kg (±SD):  78.8 (±12.3) | Patient with and without diabetes both reduced weight significantly after 15 months. No between group comparison. Excluded in meta-analysis due to follow-up time. |
| Khadanga et al. ^16^ | Baseline, kg (±SD)  90.4 ±16.7  12 to 16-week follow-up, kg (±SD):  89.4 (±16.7) | Baseline, kg (±SD)  83.7 ±16.7  12 to 16-week follow-up, kg (±SD):  81.4 (±16.1)  (no insulin resistance group) | Weight loss was higher among patients without diabetes compared to patients with diabetes (p=0.04) |
| Nishitani et al.^12^ | Baseline, kg (±SD)  48.4±9.8  6-month follow-up, kg (±SD)  45.2 ±5.2 | Baseline, kg (±SD)  49.4±7.7  6-month follow-up, kg (±SD)  49.6 ±7.3 | Weight loss tended to be higher among patients with diabetes compared to patients without diabetes. No between group comparison.  Excluded in meta-analysis due to low average body weight (Asian population) |
| Pischke et al. ^2^  Men | Baseline, kg (±SD)  95.5±18  12-week follow-up, kg (±SD)  90.9±16.7  12-month follow-up, kg (±SD)  89.9±15.6 | Baseline, kg (±SD)  85.7±15.8  12-week follow-up, kg (±SD)  81.2 ±13.3  12-month follow-up, kg (±SD)  81.0±13.1 | Significant weight change over time in both groups (no between-group comparison) |
| Pischke et al. ^2^,  Women | Baseline, ±kg (±SD)  80.3±17.4  12-week follow-up, kg (±SD)  76.4±16.3  12-month follow-up, kg (±SD)  75.2±15.5 | Baseline, kg (±SD)  75.5±17.7  12-week follow-up, kg (±SD)  70.6±16.2  12-month follow-up, kg (±SD)  69.7±16.3 | Significant weight change over time in both groups (no between-group comparison) |
| Svacinová et al. ^17^, | Baseline, kg (±SD)  86.1±13.4  12-month follow-up, kg (±SD):  86.0±12.5 | Baseline, kg (±SD)  81.3±11.5  12-month follow-up, kg (±SD):  79.7±11.00 | Patients with diabetes did not reach a significant weight loss, patients without diabetes reached significant weight loss. No comparison between groups. |

Figure 3.1.b. Meta-analysis on weight end of Cardiac Rehabilitation (stratified on intervention type)

3.1.c. Body mass index (BMI)

**Study characteristics and participants**

The review identified ten studies reporting on BMI. All studies were all observational cohort studies. The reported percentage of patients with diabetes ranged from 11.5-41.6% in the enrolled study population. In five studies, only patients with type 2 diabetes were included ^12, 16-19^. Two studies included patients with type 1 or type 2 diabetes ^5, 15^. The type of diabetes was not specified in three studies ^1, 11, 20^. In total, BMI was analysed in n=7515 patients of these, n=2424 had diabetes.

**Interventions**

CR programs were an outpatient service except for Gondoni et al where the programme was delivered as an inpatient programme ^20^. Patients were in all studies enrolled after discharge from surgical procedure. Interventions were in all studies provided at hospital or a medical center. Eser et al. was a multicentre study provided CR as both in- and outpatient programme ^5^. CR was provided as an 8-12 week program with three sessions pr. week in five studies ^1, 11, 16, 17, 19, 21^. The inpatient program in Gondoni et al. lasted on average 23 days with six sessions per week. In Nishitani et al., the intervention lasted six months with 1-2 sessions per week ^12^. In Carroll et al., the intervention lasted 15 months with three sessions per week ^15^. Eser et al., provided in 10-36 sessions in programmes lasting three weeks- three months ^5^. Besides exercise sessions, seven studies provided educational sessions concerning nutrional counselling and risk factor control ^1, 5, 11, 12, 15, 16, 20, 21^. Additionally, Khadanga et. al. offered a behavioural weight loss programme for patients with BMI >30 ^16^.

**Outcome measurement**

BMI was in all nine studies calculated as kg/m^2^. All studies measured BMI at baseline. Seven studies measured BMI at 8-12 weeks follow up ^1, 5, 11, 16-18, 21^. Nishitani et al. measured BMI at six months follow up ^12^, Eser at 12 months follow up ^5^, and Carroll et al. at 15 months follow up ^15^. In Gondoni et al., follow up was not clearly reported ^20^

**Synthesis of results**

Due to missing information ^5, 20^, heterogeneity in follow-up time ^15^ and very low baseline weight ^12^, four studies were excluded from the meta-analysis.

*Results from the meta-analysis*

Based on six studies, the overall difference in change scores between groups was 0.19 (95 % CI: 0.13; 0.26) I2= 9.62 %, p= 0.27, indicating comparable reduction in BMI in patients with diabetes compared to patients without diabetes at end of CR. According to the I2 statistics, the result might not be affected by heterogeneity. Stratification on intervention type (Figure 3.1.c) showed statistical no significant group difference (p=0.18). Test of funnel plot asymmetry was not performed due to inclusion of less than <10 studies in the meta-analysis ^10^.

*Results from studies excluded from the meta-analysis*

One study reported a significantly smaller reduction in BMI among patients with diabetes compared to patients without diabetes, however follow-up time was unclear reported ^20^. At 15-months follow-up, one study reported comparable change in BMI ^15^. One study measured both at three- to six months follow-up and at 12-months follow-up and showed comparable changes in BMI.

Figure 3.1.c. Meta-analysis on Body mass index (BMI)

| **Table 3.1.c. Descriptive results on BMI** | | | |
| --- | --- | --- | --- |
| **Author** | **Patients with diabetes** | **Patients without diabetes** | **Between-group comparison** |
| St. Clair et al. ^1^ | Baseline  31±6  Change in BMI, 95% CI (12-week follow-up)  -0.4 (-0.6;-0.2) | Baseline  28±5  Change in BMI, 95% CI (12-week follow-up)  -0.5 (-0.6;-0.4) | Comparable changes in BMI in patients with and without diabetes at end of CR (12 weeks) |
| Gondoni et al.^20^ | Change in BMI (±SD)  -1.2 ± 0.6 | Change in BMI (±SD)  1.4 ± 0.7 | Patients with diabetes reduced BMI significantly less compared to patients with diabetes (unclear number of analysed patients and follow up) |
| Hindman et al. ^11^ | Baseline BMI (±SD)  32.0±6.6  12-week follow-up (±SD)  31.9±6.4 | Baseline BMI (±SD)  29.2±5.5  12-week follow-up (±SD)  28.8±5.2 | No change in BMI among patients with diabetes, BMI decreased significantly patients with diabetes (no comparison between group) |
| Khadanga et al.^16^ | Baseline BMI (±SD)  31.2 ± 5.2  12 to 16-week follow-up (±SD)  30.8 ± 5.1 | Baseline BMI (±SD)  28.1 ± 4.6  12 to 16-week follow-up (±SD)  27.4 ± 4.4 | Patients with diabetes achieved less improvement in BMI compared to patients without diabetes (no insulin resistance) at end of CR (3-4 months). |
| Nishitani et al. ^12^ | Baseline BMI (±SD)  23.3±2.7  6-month follow-up (±SD)  22.6±1.9 | Baseline BMI (±SD)  23.4±2.9  6-month follow-up (±SD)  23.7±2.5 | Comparable changes in BMI in patients with and without diabetes at end of CR (six months) (small sample size, remarkably low BMI) |
| Svacinová et al ^17^ | Baseline BMI (±SD)  29.2±5.1  12-week follow-up (±SD)  29.2±4.8 | Baseline BMI (±SD)  28.3±3.4  12-week follow-up (±SD)  27.8±3.4 | No significant changes in BMI among patients with diabetes, however significant change in BMI among patients without diabetes at end of CR (12 weeks) |
| Toste et al.^18^ | Baseline BMI (±SD)  27.8±3.7  12-week follow-up, change in BMI (±SD)  −0.7±1.0 | Baseline BMI (±SD)  26.3±3.9  12-week follow-up, change in BMI (±SD)  −0.9±1.2 | Decrease in BMI was significantly smaller in patients with diabetes compared to patients without at end of CR (3 months) |
| Carroll et al.^15^ | Baseline BMI (±SD)  28.7±3.3  15-month follow-up (±SD)  28.3±2.9 | Baseline BMI (±SD)  27.0±3.5  15-month follow-up (±SD)  26.8±3.5 | Comparable changes in BMI in patients with and without diabetes at 15 months follow up. |
| Laddu et al.^21^ | Baseline BMI (±SD)  29.1 ± 4.9  12-week follow-up (±SD)  29.1 ± 4.9 | Baseline BMI (±SD)  28.9 ± 4.8  12-week follow-up (±SD)  28.7 ± 4.9 | Comparable changes in BMI in patients with and without diabetes at end of CR (12 weeks). |
| Eser et al. ^5^ | Results only reported in figure | Results only reported in figure | Comparable changes in BMI in patients with and without diabetes at end of CR (3-6 months) and 12 months. |

### 4.1 Lifestyle related

4.1.a Smoking status

**Study characteristics and participants**

The review identified three studies reporting on smoking status ^7, 18, 22^. All studies were observational cohort studies. The reported percentages of patients with diabetes ranged from 12-37 % in the enrolled study population. In one study, only patients with type 2 diabetes were included ^18^. The type of diabetes was not specified in two studies ^7, 22^. In total, smoking status was analysed in n=50345 patients of these, n=7454 had diabetes.

**Interventions**

CR programs were an outpatient service in all three studies ^7, 18, 22^. Patients were in all studies enrolled after discharge from surgical procedure. Interventions were in all studies provided at hospital or a rehabilitation center. CR was provided as an 6-12 week program with three sessions pr. week in two studies ^7, 18^. Number of sessions per week was not provided in Wallert et al. ^22^. Besides exercise sessions, all studies provided educational sessions concerning nutrional counselling and risk factor control ^7, 18, 22^. Smoking cessation courses or consultations were specifically described in two studies ^7, 18^.

**Outcome measurement**

Smoking status was in Suresh et al., reported as percentage of patients stopped smoking at one year (among patients smoking at time of AMI) (including risk difference and 95% CI) ^7^. In Wallert et al., smoking status was reported as smoking absenteeism (including OR and 95 % CI) at one year. In Toste et al., smoking status was reported as smoking (yes/no) in percentages at 12-weeks.

**Synthesis of results**

Due to heterogeneity in follow-up time, meta-analysis was not judged eligible. At 12-weeks follow-up, one study reported comparable reduction of smoking patients ^18^. At one-year follow-up, two studies reported that fewer patients with diabetes had stopped smoking compared to patients without diabetes ^7, 22^.

| **Table 4.1.a. Descriptive results on smoking** | | | |
| --- | --- | --- | --- |
| **Author** | **Patients with diabetes** | **Patients without diabetes** | **Between-group comparison** |
| Suresh et al.^7^ | Stopped smoking at 12-month follow-up: n=51 (54.2%) | Stopped smoking at 12-month follow-up: n= 614 (69.1%) | Fewer patients with diabetes had stopped smoking at one year compared to patients without diabetes (RD: -0.15 (-0.17-0.07) p=0.003) |
| Wallert et al.^22^ | Baseline smokers: n= 1809 (26.8 %) | Baseline smokers: n= 13,069 (32.7%) | Fewer patients with diabetes stopped smoking at 12-week follow-up (OR: 0.90 95% CI (0.81, 0.99) p= 0.035) |
| Toste et al.^18^ | Baseline smokers: n= 55(21.7%)  Reduction in smokers at 12-week follow-up: 90.9% | Baseline smokers: n= n=159 (37.1%)  Reduction in smokers at 12-week follow-up: 83.6% | Comparable reduction in smokers at 12-week follow-up (p=0.266). |

4.1.b. Physical activity

**Study characteristics and participants**

The review identified three studies reporting on physical activity ^2, 18, 23^. All studies were observational cohort studies. The reported percentage of patients with diabetes ranged from 19-40 % in the enrolled study population. Pischke et al. included patients with type 1 or type 2 diabetes (9.8 % reported with type 1 diabetes) ^2^. In two studies, only patients with type 2 diabetes were included ^18, 23^.

**Interventions**

CR programs were an outpatient service in all three studies ^2, 18^. Patients were in all studies enrolled after discharge from surgical procedure. Interventions were in all studies provided at home, hospital or a rehabilitation centre. CR was provided as an 6-12 week program with 2-3 sessions pr. week in two studies ^2, 18, 23^. In Karjalainen, structured homebased exercise sessions were performed two-three times a week ^23^. Besides exercise sessions, two studies provided educational sessions concerning nutrional counselling and risk factor control ^2, 18^, whereas Karjalainen et al. was purely exercise based ^23^.

**Outcome measurement**

Physical activity was in two studies based on self-reported measures; in Pischke et al. patients reported weekly exercise hour ^2^ and in Toste et al., physical activity was measured by International Physical Activity Questionnaire (IPAQ score)^18^. In Karjalainen et al., physical activity was measured by a wristwatch accelerometer ^23^. Toste had follow up at 12 weeks ^18^, Pieschke et al. had follow up at three and 12 months ^2^ while Karjalainen followed up at six months ^23^.

**Synthesis of results**

Due to heterogeneity in follow-up time and measurement method, meta-analysis was not judged eligible. At both 12-weeks ^2, 18^, six-months ^23^ and 12-months follow-up ^2^ comparable changes was found in physical activity.

| **Table 4.1.b. Descriptive results on physical activity** | | | |
| --- | --- | --- | --- |
|  | **Patients with diabetes** | Patients without diabetes | **Between-group comparison** |
| Karjalainen et al. ^23^ | Daily activity at moderate intensity, hours ±SD:  Baseline: 2.40 ± 1.23  Six-month follow-up: 2.36 ±1.06 | Daily activity at moderate intensity, hours ±SD:  Baseline: 3.24 ± 1.17  Six-month follow-up: 3.43 ± 1.21 | Comparable changes in moderate and high intensity physical activity between patients with and without diabetes at six months follow up (p value not significant) |
| Pischke et al.^2^ Men | Exercise hr/wk  Baseline ±SD  1.8±1.7  12-week follow-up ±SD  4.0±3.3  12-month follow-up ±SD  3.8±2.5 | Exercise hr/wk  Baseline ±SD  2.4±2.0  12-week follow-up, ±SD  4.1±2.1  12-month follow-up, ±SD  3.6±2.1 | Comparable changes in physical activity between patients with and without diabetes 12 weeks and 12 months follow up |
| Pischke et al.^2^  *Women* | Exercise hr/wk  Baseline ±SD  1.1 ± 1.1  12-week follow-up ±SD  3.0±1.3  12-month follow-up ±SD  2.8±1.4 | Exercise hr/wk  Baseline, (±SD)  1.6±1.5  12-week follow-up, (±SD)  3.3±1.5  12-month follow-up, (±SD)  3.0±1.7 | Comparable changes in physical activity between patients with and without diabetes 12 weeks and 12 months follow up (female patients with diabetes exercised less) |
| Toste et al.^18^ | IPAQ,score, change from baseline, median: 1283 | IPAQ,score, change from baseline, median: 1380 | Comparable changes in physical activity between patients with and without diabetes at 12 weeks follow up (p=0.628). |

### 5.1 Well-being

5.1. Psychological well-being

No study was identified reporting on psychological well-being.

### 6. Work related

6.1.a Return to work

**Study characteristics and participants**

The review identified one observational study by Suresh et al. reporting on return to work ^7^.

The percentage of patients with diabetes was 12.4 % in the study population. The type of diabetes among diabetes patients was not specified.

The study included in total n=1804 of these, n=223 had diabetes. However, the number of analysed patients on return to work was not specified.

**Interventions**

The CR program was delivered as an outpatient service with patients being enrolled after discharge from surgical procedure. The intervention was provided at hospital as a six week program with three sessions pr. week. Besides exercise sessions, educational components regarding life style modification were provided ^7^.

**Outcome measurement**

Information on return to work was retrieved from a database and performed on patients who were employed at the event of acute myocardial infarction. Estimates were reported as percentages and risk difference with 95 % confidence intervals ^7^.

**Results**

| Table 6.1.a. Descriptive results on return to work | | | |
| --- | --- | --- | --- |
|  | **Patients with diabetes** | **Patients without diabetes** | **Between-group comparison** |
| Suresh et al., ^7^ | Returned to work n=33(47.8%) | n=353(52.7) | Similar return to work percentages in patients with diabetes compared to patients without diabetes at one year. RD: -0.05, 95% CI (-0.03,-0.04), p=0.44 |

1. Ferrans and Powers Quality of Life Index Cardiac version IV questionnaire at baseline and at 12 weeks [↑](#footnote-ref-1)
2. Medical Outcomes Study shortform health survey (MOS SF-36) at baseline, three months and 12 months [↑](#footnote-ref-2)
3. Glucose, mg/dL [↑](#footnote-ref-3)
4. HbA1c [↑](#footnote-ref-4)
5. HbA1c, % (sd) [↑](#footnote-ref-5)
6. HbA1c mmol/mol [↑](#footnote-ref-6)
