# Supplementary material for: Benefits of cardiac rehabilitation following acute coronary syndrome for patients with and without diabetes: a systematic review and meta-analysis
Source: BMC Cardiovasc Disord. 2022 Jun 27;22:295. doi: 10.1186/s12872-022-02723-5 (PMC9237976; doi:10.1186/s12872-022-02723-5)
Supplement: Supplementary file 3 — Additional file 3.. Secondary outcomes. [file 12872_2022_2723_MOESM3_ESM.docx]

### References (Additional file 3)

1. St. Clair M, Mehta H, Sacrinty M, Johnson D, Robinson K. Effects of Cardiac Rehabilitation in Diabetic Patients: Both Cardiac and Noncardiac Factors Determine Improvement in Exercise Capacity : Effects of CR in diabetic patients. Clinical cardiology 2014;**37**(4):233-238.

2. Pischke CR, Weidner G, Elliott-Eller M, Scherwitz L, Merritt-Worden TA, Marlin R, Lipsenthal L, Finkel R, Saunders D, McCormac P, Scheer JM, Collins RE, Guarneri EM, Ornish D. Comparison of Coronary Risk Factors and Quality of Life in Coronary Artery Disease Patients With Versus Without Diabetes Mellitus. The American Journal of Cardiology 2006;**97**(9):1267-1273.

3. Ferrans CE PM. Quality of Life Index. Chicago, IL: College

of Nursing, University of Illinois at Chicago; 1998–1984.

4. Giallauria F, Fattirolli F, Tramarin R, Ambrosetti M, Griffo R, Riccio C, De Feo S, Piepoli MF, Vigorito C. Clinical characteristics and course of patients with diabetes entering cardiac rehabilitation. Diabetes Research and Clinical Practice 2015;**107**(2):267-272.

5. Eser P, Marcin T, Prescott E, Prins LF, Kolkman E, Bruins W, van der Velde AE, Pena-Gil C, Iliou MC, Ardissino D, Zeymer U, Meindersma EP, Van'tHof AWJ, de Kluiver EP, Laimer M, Wilhelm M. Clinical outcomes after cardiac rehabilitation in elderly patients with and without diabetes mellitus: The EU-CaRE multicenter cohort study. Cardiovascular Diabetology 2020;**19**(1).

6. Reibis R, Treszl A, Bestehorn K, Karoff M, Schwaab B, Wirth A, von Horlacher J, Jannowitz C, Pittrow D, Wegscheider K, Völler H. Comparable short-term prognosis in diabetic and non-diabetic patients with acute coronary syndrome after cardiac rehabilitation. Eur J Prev Cardiol 2012;**19**(1):15-22.

7. Suresh V, Harrison RA, Houghton P, Naqvi N. Standard cardiac rehabilitation is less effective for diabetics. International journal of clinical practice 2001;**55**(7):445-448.

8. Yu CM, Lau CP, Cheung BMY, Fong YM, Ho YY, Lam KB, Li LSW. Clinical predictors of morbidity and mortality in patients with myocardial infarction or revascularization who underwent cardiac rehabilitation, and importance of diabetes mellitus and exercise capacity. American Journal of Cardiology 2000;**85**(3):344-349.

9. Yu CM, Li LSW, Lam MF, Siu DCW, Miu RKM, Lau CP. Effect of a cardiac rehabilitation program on left ventricular diastolic function and its relationship to exercise capacity in patients with coronary heart disease: Experience from a randomixed, controlled study. American Heart Journal 2004;**147**(5):e24-e24.

10. Page MJ HJ, Sterne JAC. Chapter 13: Assessing risk of bias due to missing results in a synthesis. In: Higgins JPT TJ, Chandler J, Cumpston M, Li T, Page MJ, Welch VA (editors). (ed). *Cochrane Handbook for Systematic Reviews of Interventions version 6.2*: Cochrane, 2021.

11. Hindman L, Falko JM, LaLonde M, Snow R, Caulin-Glaser T. Clinical profile and outcomes of diabetic and nondiabetic patients in cardiac rehabilitation. American Heart Journal 2005;**150**(5):1046-1051.

12. Nishitani M, Shimada K, Masaki M, Sunayama S, Kume A, Fukao K, Sai E, Onishi T, Shioya M, Sato H, Yamamoto T, Amano A, Daida H. Effect of cardiac rehabilitation on muscle mass, muscle strength, and exercise tolerance in diabetic patients after coronary artery bypass grafting. Journal of Cardiology 2013;**61**(3):216-221.

13. Karjalainen JJ, Kiviniemi AM, Hautala AJ, Piira OP, Lepojärvi ES, Perkiömäki JS, Junttila MJ, Huikuri HV, Tulppo MP. Effects of physical activity and exercise training on cardiovascular risk in coronary artery disease patients with and without type 2 diabetes. Diabetes Care 2015;**38**(4):706-15.

14. Banzer JA, Maguire TE, Kennedy CM, O'Malley CJ, Balady GJ. Results of cardiac rehabilitation in patients with diabetes mellitus. The American Journal of Cardiology 2004;**93**(1):81-84.

15. Carroll S, Tsakirides C, Hobkirk J, Moxon JWA, Moxon JWD, Dudfield M, Ingle L. Differential Improvements in Lipid Profiles and Framingham Recurrent Risk Score in Patients With and Without Diabetes Mellitus Undergoing Long-Term Cardiac Rehabilitation. Archives of Physical Medicine and Rehabilitation 2011;**92**(9):1382-1387.

16. Khadanga DS, Savage AP, Ades AP. Insulin Resistance and Diabetes Mellitus in Contemporary Cardiac Rehabilitation. Journal of Cardiopulmonary Rehabilitation and Prevention 2016;**36**(5):331-338.

17. Svacinova, aacute, Hana, Nov, aacute, kov, aacute, Marie, Placheta Z, Kohzuki M, Nagasaka M, Minami N, Dob, scaron, aacute, k P, Siegelov, aacute, Jarmila. Benefit of Combined Cardiac Rehabilitation on Exercise Capacity and Cardiovascular Parameters in Patients with Type 2 Diabetes. The Tohoku Journal of Experimental Medicine 2008;**215**(1):103-111.

18. Toste S, Viamonte S, Barreira A, Fernandes P, Gomes JL, Torres S. Cardiac rehabilitation in patients with type 2 diabetes mellitus and coronary disease: A comparative study. Revista Portuguesa De Cardiologia 2014;**33**(10):599-608.

19. Laddu D, Ozemek C, Lamb B, Hauer T, Aggarwal S, Stone JA, Arena R, Martin B-J. Factors Associated With Cardiorespiratory Fitness at Completion of Cardiac Rehabilitation: Identification of Specific Patient Features Requiring Attention. Canadian Journal of Cardiology 2018;**34**(7):925-932.

20. Gondoni LA, Titon AM, Nibbio F, Caetani G, Augello G, Mian O, Tuzzi C, Averna E, Parisio C, Liuzzi A. Short-term effects of a hypocaloric diet and a physical activity programme on weight loss and exercise capacity in obese subjects with chronic ischaemic heart disease: a study in everyday practice. Acta Cardiol 2008;**63**(2):153-9.

21. Laddu DR, Ozemek C, Hauer TL, Rouleau CR, Campbell TS, Wilton SB, Aggarwal S, Austford L, Arena R. Cardiometabolic responses to cardiac rehabilitation in people with and without diabetes. International Journal of Cardiology 2020;**301**:156-162.

22. Wallert J, Mitchell A, Held C, Hagstrom E, Leosdottir M, Olsson EG. Cardiac rehabilitation goal attainment after myocardial infarction with versus without diabetes: A nationwide registry study. International journal of cardiology 2019;**292**:19-24.

23. Karjalainen JJ, Kiviniemi AM, Hautala AJ, Niva J, Lepojarvi S, Makikallio TH, Piira O-P, Huikuri HV, Tulppo MP. Effects of exercise prescription on daily physical activity and maximal exercise capacity in coronary artery disease patients with and without type 2 diabetes. Clinical Physiology and Functional Imaging 2012;**32**(6):445-454.
