# Supplementary material for: Benefits of cardiac rehabilitation following acute coronary syndrome for patients with and without diabetes: a systematic review and meta-analysis
Source: BMC Cardiovasc Disord. 2022 Jun 27;22:295. doi: 10.1186/s12872-022-02723-5 (PMC9237976; doi:10.1186/s12872-022-02723-5)
Supplement: Supplementary file 4 — Additional file 4. Exposure measurement methods, classification of diabetes status. [file 12872_2022_2723_MOESM4_ESM.docx]

### Additional file 4) Exposure measurement methods, classification of diabetes status

| Reference | Method of classification as reported in study | Measured exposure |
| --- | --- | --- |
| Banzer et al, 2003, USA^27^ | Self-reported history of diabetes or taking medication for glycemic control | Diabetes, type not specified (yes/no) |
| Vergès et al 2003, France ^33^ | At least two fasting plasma glucose levels >7 mmol/l. | Diabetes, type 2 only (yes/no) |
| Hindman et al., 2005, USA ^32^ | Self-reported history of diabetes or taking diabetic medication | Diabetes, type not specified (yes/no) |
| Pischke et al 2006, USA ^31^ | Limited information, diagnosed according to guidelines of the American Diabetes Association) | Diabetes, type 1 or type (yes/no) |
| Svacinová et al 2008 Czech Republic ^30^ | No information | Diabetes, type 2 diabetes only (yes/no) |
| Mourot et al 2010, France ^34^ | Fasting blood glucose concentration greater than 126 mg/dL on at least two occasions and/or antidiabetic treatment. | Diabetes, type 2 only (yes/no) |
| Karjalainen et al 2012, Finland ^35^ | Defined according to Worlds Health Organization criteria, 1999: increased fasting plasma glucose level (two-hour glucose tolerance test), or taking hypoglycemic medication. | Diabetes, type 2 only (yes/no) |
| Wu et al 2012, Taiwan ^28^ | (from Wu, 2008 paper (ref 8): Plasma glucose 6 200 mg/dl (11.1 mM), fasting plasma glucose 6 126 mg/dl (7.0 mM), or under oral hypoglycemic agents and/or insulin treatment. | Diabetes, type 1 or type 2 (yes/no) |
| St. Clair et al, 2013, USA ^29^ | Diagnosed using standard criteria by American Diabetes Association, (ref. 16)). Clinical data from patient records. | Diabetes, type not specified (yes/no) |
| Nishitani et al 2013 Japan ^36^ | Medical treatment, fasting plasma glucose ≥126 mg/dl or casual plasma glucose ≥, or HbA1 ≥ 6.1. Criteria according to Japan Diabetes Society | Diabetes, type not specified (yes/no) |
| Toste et al 2013, Portugal ^37^ | A history of diabetes from medical records or under antidiabetic therapy | Diabetes, type 2 only (yes/no) |
| Armstrong et, al, 2014, Canada ^39^ | Patient reported and hospital chart. Diagnosis confirmed based on hospital coded administrative databases. | Diabetes, type 1 or type 2 diabetes (yes/no) |
| Kenttä et al 2014, Finland ^38^ | No information | Diabetes, type 2 only (yes/no) |
| Kim et al, 2015, Korea^41^ | Fastening plasma glucose test or hemoglobin A1c (HbA1c). | Diabetes, type 2 only (yes/no) |
| Szalewska et al 2015 Poland ^42^ | No information | Diabetes, type 2 only (yes/no) |
| Boukhris et al., 2015, Italy ^40^ | All non-diabetic patients were screened for diabetes before enrollment using fasting serum glucose and glycosylated hemoglobin. | Diabetes, type 2 only (yes/no) |
| Khadanga et al, 2017 USA ^43^ | Fasting serum glucose of ≥126 mg/dL or HbA1c ≥ 6.5 (based on American Diabetes Association). | Diabetes, type 2 only (No insulin resistance, insulin resistance, Type 2 DM) |
| Kasperowicz ^44^ | No information | Diabetes, type 2 only (yes/no) |
| Laddu et.al. 2020, Canada ^45^ | History of type 2 diabetes diagnosed or treated by a physician, or diabetes indicated in hospital record. Diagnosis cross-checked in database and registers to verify. | Diabetes, type 2 only (yes/no) |
| Eser ^46^ | Previous diagnosis with diabetes, insulin or oral antidiabetics, or HbA1c at baseline of ≥ 48 mmol/mol. | Diabetes, type 1 or type 2 (yes/no) |
