# Supplementary material for: Benefits of cardiac rehabilitation following acute coronary syndrome for patients with and without diabetes: a systematic review and meta-analysis
Source: BMC Cardiovasc Disord. 2022 Jun 27;22:295. doi: 10.1186/s12872-022-02723-5 (PMC9237976; doi:10.1186/s12872-022-02723-5)
Supplement: Supplementary file 5 — Additional file 5. Outcome measurement methods, exercise capacity. [file 12872_2022_2723_MOESM5_ESM.docx]

### Additional file 5) Outcome measurement methods, exercise capacity

|  |  | | | | | |
| --- | --- | --- | --- | --- | --- | --- |
| Outcome measurement method listed in the study | | | | | |  |
| Reference | Procedure | Mode of test; Loading of resistance | Estimated or directly cardiopulmonary exercise testing maximal | Measured outcome | Remarks on reported and analysed results |  |
| Banzer et al., 2003, USA^27^ | Exercise testing in a standard manner using symptom-limited individualized ramp treadmill protocols. Peak MET level was estimated from peak exercise workrate. | Treadmill; ramp | Estimated | METs |  |  |
| Vergès et al. 2003, France ^33^ | Initial workload: 10 W, Increments of 10 W at each 1-min. Cardiopulmonary data, including peak VO2 and VO2 at anaerobic threshold. The obtained data were duration of the test (min), peak workload (W), maximal heart rate, peak VO2 (ml/kg per min) and anaerobic threshold (ml/kg per min). | Bicycle ergometer; incremental | Directly | VO_2_peak | VO_2_ peak was converted into METs assuming that 1 MET equals 3.5 ml/min/kg resting oxygen. |  |
| Hindman et al., 2005, USA ^32^ | Graded symptom-limited maximal stress test according to a standard Bruce protocol at entry and upon program completion to calculate exercise capacity in metabolic equivalent time. | Treadmill; unknown | Estimated | METs |  |  |
| Pischke et al. 2006, USA ^31^ | Symptom-limited maximal graded exercise testing using the Bruce protocol. METs were automatically calculated by the testing device during exercise testing | Treadmill; unknown | Estimated | METs | Provided estimates are stratified on gender in paper. For the purpose of meta-analysis, gender stratified mean difference on exercise capacity have been treated as two separate study populations, referred to as Pischke, Male and Pischke, Female |  |
| Svacinová et al. 2008 Czech Republic ^30^ | Progressively increasing working rate to maximal tolerance. Test starting at workload 30 watts with progressively increasing working rate (20 W/2 min). Oxygen uptake and carbon dioxcide production were measured by analysis of blood gas samples taken breath by breath. | Bicycle ergometer; unknown | Directly | VO_2_peak | VO_2_ peak was converted into METs assuming that 1 MET equals 3.5 ml/min/kg resting oxygen. |  |
| Mourot et al. 2010, France ^34^ | The exercise stress test with a gas exchange measurement. The exercise stress test provided measurement of peak oxygen uptake (VO2max) and ventilatory threshold. | Treadmill; ramp | Directly | VO_2_ max | Results on METs were originally provided stratified on interventional procedure (CAGB/PTCA). Unified data were kindly provided upon request by corresponding author.  VO2 peak was converted into METs assuming that 1 MET equals 3.5 ml/min/kg resting oxygen.  Relatively low VO2 max values might be due to the use of a ramp protocol resulting in a more exhaustive test procedure. |  |
| Karjalainen et al. 2012, Finland ^35^ | Symptom-limited maximal exercise test. Test start: 30 W, increments at 15 W (men) and 10 W (women) each 1 minute, until voluntary exhaustion or ST depression > 0·2 mV in ECG. Gas exchange monitored continuously. The highest 1-min mean value of oxygen consumption = VO2peak. Maximal workload (W) and maximal metabolic equivalents (METs) were calculated as the average workload and METs during the last minute of the test. | Bicycle ergometer; incremental | Directly | VO_2_peak (ml/kg-1xmin-1) + METsmax | VO_2_ peak was converted into METs assuming that 1 MET equals 3.5 ml/min/kg resting oxygen. Lower exercise capacity values might be due to the use of bicycle ergometer. |  |
| Wu et al. 2012, Taiwan ^28^ | Test starting af 25 W, increments every 3 minutes, Pedaling rate of 50 to 60 rpm. Until volitional fatigue, heart rate at 90 % of the maximum, a respiratory exchange ratio >1.15, or signs of exercise intolerance. VO_2_ peak was measured by a metabolic Measurement System using breath by breath technique. | Bicycle ergometer; incremental | Directly | VO_2_ peak | The study was excluded for the meta-analysis due to lack of estimates provided in the paper. Corresponding author was contacted by email unsuccessfully. |  |
| St. Clair et al., 2013, USA ^29^ | Steady-state MET levels were recorded at each session and were obtained either automatically from exercise devices or using a standardized MET formula. | Unknown; Unknown | Estimated | METs | The study have been excluded for the meta-analysis due to sparse reporting of test procedure. Reported METs are notably lower than expected. |  |
| Nishitani et al. 2013 Japan ^36^ | Testing using an expiratory gas analysis machine.  Starting at 20 W, followed by loading (15 W/min) until exhaustion, progressive angina, ST-segment depression (≥2 mm), or sustained tachyarrhythmia. | Bicycle ergometer; ramp | Directly | Peak VO_2_ | Lower exercise capacity values might be explained by a high prevalence of comorbidity in the study population:  Ejection fraction: DM=59.7±16 No DM= 65.3±12. Off-pump CABG surgery: DM=97 % No DM= 100 %. |  |
| Toste et al. 2013, Portugal ^37^ | Maximal functional capacity was estimated, based on the metabolic equations of the American College of Sports Medicine for treadmill exercise. | Treadmill; unknown | Estimated | METs |  |  |
| Armstrong et, al, 2014, Canada ^39^ | Bruce or modified Bruce protocol. Peak estimated MET value calculated from speed and grade at final stage of the exercise protocol using an established equation. | Treadmill; unknown | Estimated | METs | Provided estimates are stratified on gender in paper. For the purpose of meta-analysis, gender stratified mean difference on exercise capacity have been treated as two separate study populations, referred to as Armstrong, Male and Armstrong, Female |  |
| Kenttä et al. 2014, Finland ^38^ | Symptom-limited test. Initial workload of 30 W with a gradual increase of load in steps of 10 (women) and 15 W/min (men). Testing was continued until voluntary exhaustion or ST-segment depression exceeding 0.2 mV. | Bicycle ergometer; unknown | Directly | METs | Low exercise capacity values might be due to the use of bicycle ergometer. |  |
| Kim et al., 2015, Korea^41^ | Modified symptom limited Bruce protocol. Respiratory gas analyzer, METs during the maximal exercise period and peak oxygen consumption (VO2peak). | Treadmill; unknown | Directly | VO_2_peak, (mL/kg/min) + METs | For the purpose of meta-analysis, provided METs in paper have been used. |  |
| Szalewska et al. 2015 Poland ^42^ | Symptom-limited exercise test according to the Bruce protocol. Monitored during test: maximal workload (METs), heart rate, blood pressure (BP, mm Hg) at rest and at maximal effort. | Treadmill; unknown | Estimated | METs |  |  |
| Boukhris et al., 2015, Italy ^40^ | Maximal exercise testing, using ramp protocol with maximal metabolic equivalents (METs) measurement. | Treadmill; ramp | Estimated | METs |  |  |
| Khadanga et al., 2017 USA ^43^ | Symptom limited exercise tolerance test. Peak metabolic equivalents were estimated based on treadmill speed and elevation. For 517 patients, expired gas was analyzed during the exercise protocol. Peak VO2 was considered to be the highest 30-second average during the test. | Treadmill; unknown | Estimated /Directly | Peak V̇O_2_ (mL O_2_kg/min) + METs | For the purpose of meta-analysis, VO_2_ peak was converted into METs assuming that 1 METs equals 3.5 ml/min/kg resting oxygen. |  |
| Kasperowicz et.al., 2019 ^44^ | Means of exercise ECG test according to Bruce protocol | Treadmill; unknown | Estimated | METs |  |  |
| Laddu et.al. 2020, Canada ^45^ | Symptom-limited maximal test to determine peak metabolic equivalents (METs). The peak MET value was calculated from speed and grade during the final stage of the exercise protocol using an established equation. | Treadmill; unknown | Estimated | METs |  |  |
| Eser et al., 2020 | Cardiopulmonary exercise test with ergometry, electrocardiogram registration. Initial workload: 5 WATT for 3 min. Individual RAMP protocol was applied for optimal duration of 8–12 min. | Bicycle, Treadmill*; ramp | Directly* | VO_2_ peak | *Data collected from baseline study ^62^: Treadmill was used in seven patients. Due to mask intolerance, signs of mask leakage or short test duration, VO_2_peak was calculated using the maximum watts in 102 patients ^62^. A smaller fraction of the study population (n >48 patients: 3,6 % was tested by 6 min walking test)^62^  VO_2_ peak was converted into METs assuming that 1 MET equals 3.5 ml/min/kg resting oxygen. |  |
