# Supplementary material for: Benefits of cardiac rehabilitation following acute coronary syndrome for patients with and without diabetes: a systematic review and meta-analysis
Source: BMC Cardiovasc Disord. 2022 Jun 27;22:295. doi: 10.1186/s12872-022-02723-5 (PMC9237976; doi:10.1186/s12872-022-02723-5)
Supplement: Supplementary file 6 — Additional file 6. Subgroup analysis. [file 12872_2022_2723_MOESM6_ESM.docx]

### Additional file 6) Subgroup analysis

#### Figure AD1 Forest plot of subgroup analysis on type of diabetes


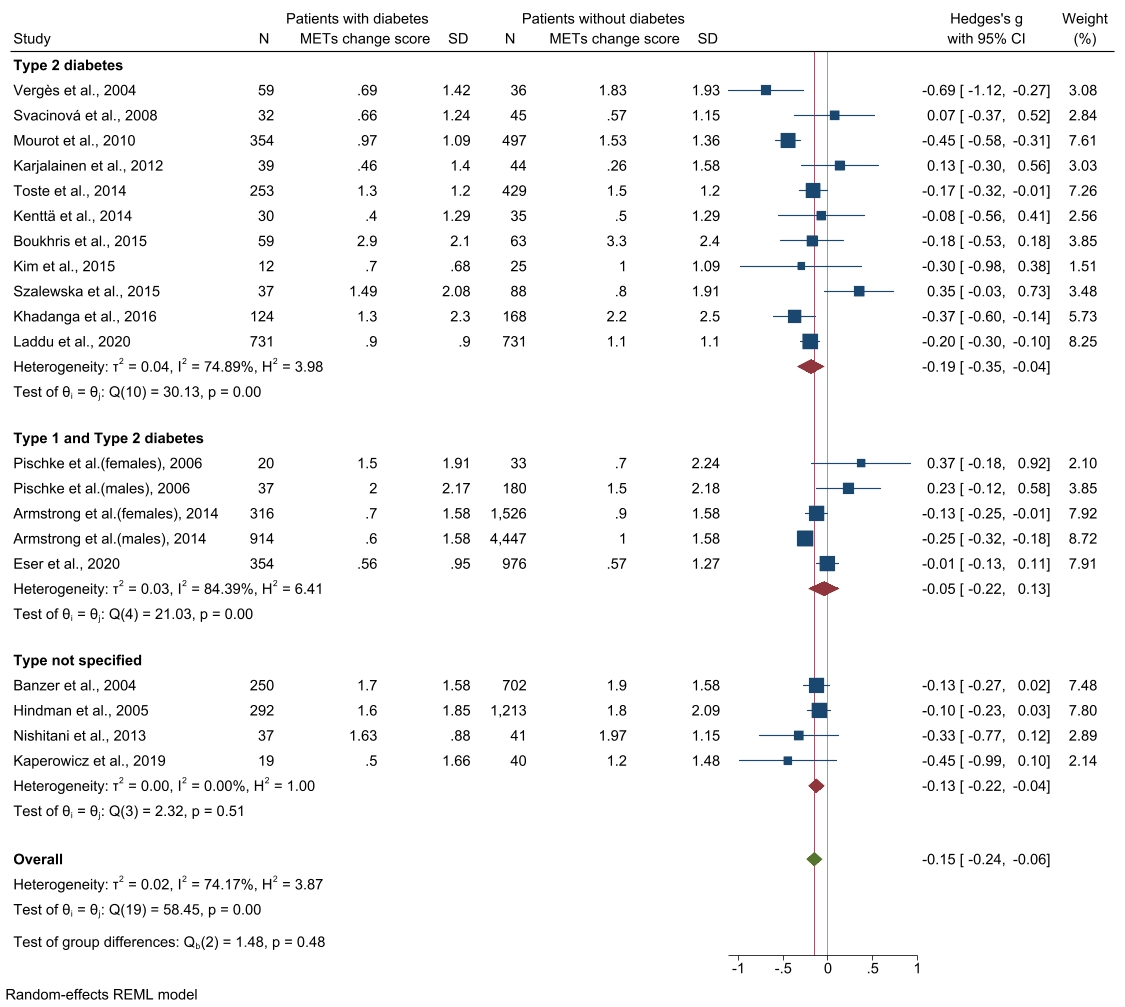


#### Figure AD2 Subgroup analysis on type of intervention


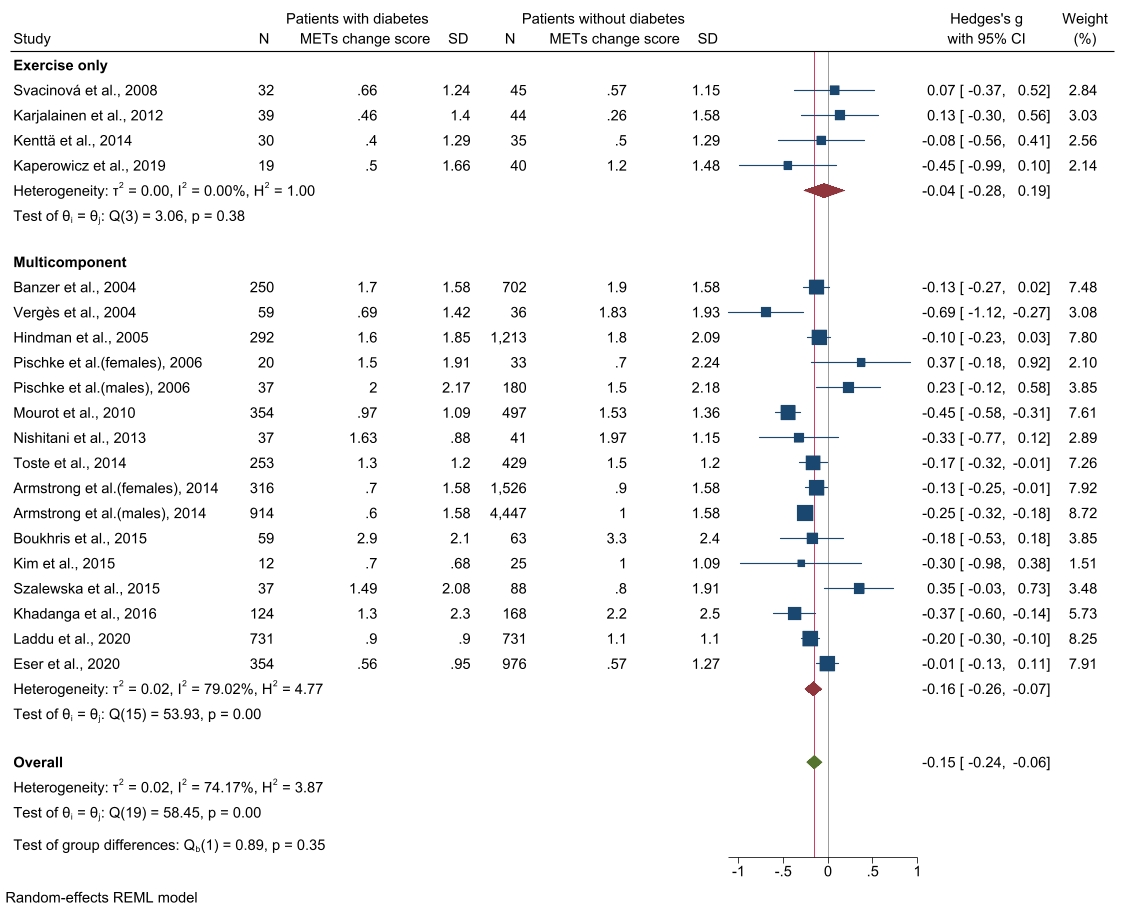


#### Figure AD3 Subgroup analysis on length of trial follow up

#### ****

#### Figure SM6.4 Funnel plot for assessing publication bias from start of CR to end of intervention

#### Figure AD6.5 Funnel plot for assessing publication.bias
